# Mysterious sphingolipids: metabolic interrelationships at the center of pathophysiology

**DOI:** 10.3389/fphys.2023.1229108

**Published:** 2024-01-03

**Authors:** Rama Jamjoum, Saurav Majumder, Batoul Issleny, Johnny Stiban

**Affiliations:** ^1^ Department of Pharmacy, Birzeit University, West Bank, Palestine; ^2^ National Institute of Allergy and Infectious Diseases (NIAID), National Institutes of Health, Rockville, MD, United States; ^3^ Department of Biology and Biochemistry, Birzeit University, West Bank, Palestine

**Keywords:** ceramide, sphingolipids, metabolism, inhibitors, physiology, pathophysiology, metabolic interrelationships

## Abstract

Metabolic pathways are complex and intertwined. Deficiencies in one or more enzymes in a given pathway are directly linked with genetic diseases, most of them having devastating manifestations. The metabolic pathways undertaken by sphingolipids are diverse and elaborate with ceramide species serving as the hubs of sphingolipid intermediary metabolism and function. Sphingolipids are bioactive lipids that serve a multitude of cellular functions. Being pleiotropic in function, deficiency or overproduction of certain sphingolipids is associated with many genetic and chronic diseases. In this up-to-date review article, we strive to gather recent scientific evidence about sphingolipid metabolism, its enzymes, and regulation. We shed light on the importance of sphingolipid metabolism in a variety of genetic diseases and in nervous and immune system ailments. This is a comprehensive review of the state of the field of sphingolipid biochemistry.

## 1 Introduction

**FIGURE 1:**
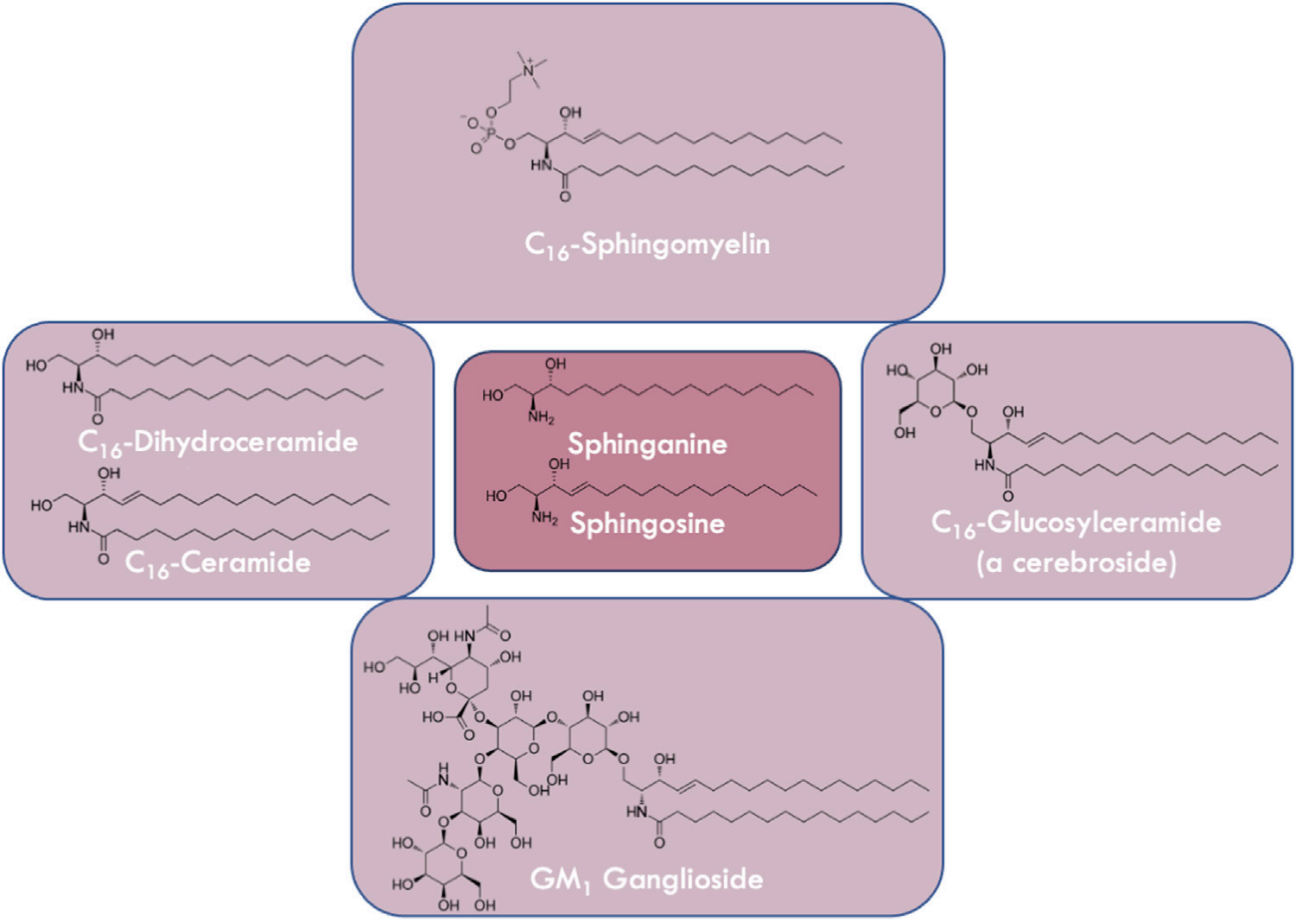
Variations in the structures of sphingolipids. Sphingolipids are derivatives of the long chain bases sphingosine or sphinganine (center panel). Ceramides and dihydroceramides (left panel) which are *N*-acylated sphingosines or sphinganines, respectively, vary in fatty acyl chain length. Glucosylceramide is a cerebroside (right panel) containing a monosaccharide headgroup on ceramide, whereas gangliosides (e.g., GM_1_ (bottom panel)) contain oligosaccharides including sialic acid residues. Sphingomyelin (top panel) is a phospholipid that contains a zwitterionic phosphocholine headgroup attached to ceramide.

Sphingolipids represent a group of structurally diverse lipid molecules (Futerman and Hannun, 2004) (Figure 1). These amphipathic lipids are one of the major components of almost all eukaryotic membranes (Quinville et al., 2021). In addition to their structural roles, some sphingolipid metabolic intermediates act as critical signaling molecules involved in a wide range of biological processes (Abou-Ghali and Stiban, 2015; Abed Rabbo et al., 2021). Of particular interest are the sphingolipids sphingosine 1-phosphate (S1P), ceramide (Cer), and ceramide 1-phosphate (C1P) which are labeled as bioactive and signaling hubs (Issleny et al., 2023). These molecules have been directly and indirectly implicated in cell-cell interactions, cellular migration, senescence, and cell death (Spiegel and Milstien, 2003; Mendelson et al., 2014), to name a few critical processes. The term “sphingolipid” was coined by J. L. W. Thudichum because of the enigmatic nature of these lipids (Stiban, 2019). Unlike glycerophospholipids which contain a glycerol backbone, sphingolipids built on a unique amino-alcohol sphingoid base (Pruett et al., 2008). The sphingoid backbone is capable of rendering additional structural diversity to these lipids (Breslow and Weissman, 2010). Almost a century after their discovery, scientists started to uncover additional roles of sphingolipid in a wide range of cellular functions and their involvement in maintaining normal physiology (Hannun and Bell, 1989). Recently, the advent of cutting-edge technologies such as genome/proteome sequencing, mass-spectrometry, and super-resolution microscopy coupled with genetic or functional screens in human or other higher eukaryotic systems using CRISPR/Cas9, started a new era of identifying and assigning novel functions to sphingolipids.

Sphingolipids are found in almost all eukaryotes (Heung et al., 2006) ranging from unicellular yeast to higher eukaryotes such as humans and plants. They have even been identified in some prokaryotes (*Bacteroidetes*) (Rappocciolo and Stiban, 2019; Johnson et al., 2020), suggesting that enzymes required for their metabolism are also highly conserved. In fact, homologs of these enzymes can be found in almost all eukaryotes. Different eukaryotes exhibit distinct sphingolipid profiles. In addition, complex eukaryotic systems such as humans or mice show distinct tissue-specific representations of different sphingolipid species (Laviad et al., 2008), suggesting that each species may have unique properties required for specialized functions. This is consistent with a higher degree of complexity in subunit compositions of several key sphingolipid metabolic enzymes and their regulating partners.

Sphingolipid metabolic pathways are complex and interconnected, with multiple metabolites feeding in and out of these pathways (Santos et al., 2022). This interconnectivity makes it difficult to pinpoint the significance of any one metabolite species. Intracellular pools of different sphingolipid species exist as a dynamic equilibrium between the *de novo* biosynthetic pathway and the salvage pathway. Despite all the challenges, in the last few decades, our understanding of their regulation and physiological roles made great advances. Several new regulatory subunits of key sphingolipid metabolic enzymes were identified, along with novel regulatory mechanisms. In complex higher eukaryotic model systems (such as in plants, flies, worms, or mice) chemical or genetic manipulation of different metabolic enzymes helped us better understand the importance of sphingolipids in maintaining normal physiology. Genome-wide association studies have revealed mutations in sphingolipid metabolic genes can be correlated to diseases such as Alzheimer’s, Parkinson’s, or Amyotrophic Lateral Sclerosis which were previously not linked.

In this article, we aim to provide an in-depth review of sphingolipid metabolism, the enzymes involved, and how different mutations in the subunits of these enzymes impact sphingolipid metabolism resulting in pathophysiological conditions. A detailed understanding of the basic architecture of the sphingolipid metabolic pathway is critical to gain important insights and address important questions.

## 2 Sphingolipid metabolism

**FIGURE 2:**
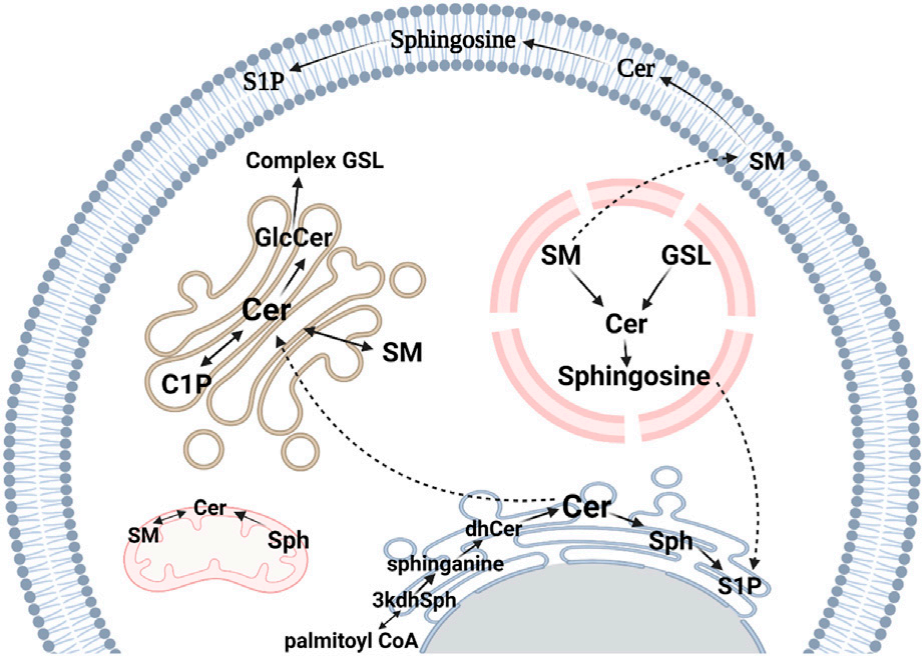
Sphingolipid metabolic pathways in eukaryotes. The *de novo* synthesis pathway occurs on the cytosolic surface of the ER. It starts with the condensation of _L_-serine and palmitoyl-CoA to produce 3-ketosphinganine (3kdhSph) which is reduced by 3-ketosphinganine reductase to form sphinganine. Sphinganine is then *N*-acylated to dihydroceramide (dhCer) by CerS and in the final step, ceramide (Cer) is synthesized by the action of dihydroceramide desaturase, which is responsible for converting the reduced sphingoid backbone of sphinganine into a monounsaturated sphingosine (Sph) backbone. Sphingosine kinases catalyze the phosphorylation of Sph to sphingosine 1-phosphate (S1P), a paracrine signaling molecule. Cer is transported to the Golgi apparatus by Cer transport protein where it becomes a substrate for sphingomyelin (SM) synthesis by SM synthase. Alternatively, Cer can be a substrate for glucosylceramide (GlcCer) generation, a precursor for other glycosphingolipids (GSLs). Cer can be phosphorylated to Cer 1-phosphate (C1P) by Cer kinase. GSLs and SM can be transported to the endosomal system and metabolized by lysosomal degradation. This figure was created with Biorender.com using a paid student subscription.

In mammals, as well as in other eukaryotic cells, sphingolipids are turned over via three distinct pathways (Figure 2). A series of membrane-embedded enzymes coordinate, control, and regulate the different processes of sphingolipid metabolism, with Cer serving as the precursor molecule of other sphingolipids and their metabolic hub (Breslow and Weissman, 2010; Gault et al., 2010; Pralhada Rao et al., 2013). Once Cer is synthesized, it can be used as a precursor for the synthesis of other sphingolipids, such as sphingomyelin (SM), glucosylceramide (GlcCer), and gangliosides (Bleicher and Cabot, 2002; Taniguchi and Okazaki, 2021; DAngelo et al., 2018). SM is synthesized by the transfer of a phosphocholine head group from phosphatidylcholine to Cer, while GlcCer is synthesized by the addition of a glucosyl group to the hydroxyl headgroup of Cer. Gangliosides are synthesized by the stepwise addition of different sugar moieties to Cer (Hernández-Bello et al., 2023).

Complex sphingolipids are catabolized to Cer which can then be further metabolized to sphingosine, which can be phosphorylated to S1P. S1P can be ultimately cleaved into phosphoethanolamine and hexadecenal which has been shown to interfere and form adducts with several biological molecules (Kumar et al., 2011; Upadhyaya et al., 2012; Schumacher et al., 2017). This process is carried out by a group of sphingolipid hydrolases, including sphingomyelinases (SMases), glucocerebrosidases, gangliosidases, and S1P lyases.

### 2.1 *De novo* synthetic pathway

The pathway of *de novo* synthesis initiates with the combination of _L_-serine, an amino acid, and palmitoyl-CoA, resulting in the generation of 3-ketosphinganine, which subsequently undergoes reduction at its ketone group, leading to the formation of sphinganine (Gault et al., 2010). Sphinganine then undergoes *N*-acylation by Cer synthases (CerSs) (Lahiri and Futerman, 2005; Stiban et al., 2010; Zelnik et al., 2019; Kim et al., 2021), resulting in the production of dhCer, which is oxidized in the final step through the introduction of a 4,5-*trans* double bond in the fatty acid portion of the molecule, leading to the creation of Cer (Kraveka et al., 2007; Rodriguez-Cuenca et al., 2015; Harrison et al., 2018; Alsanafi et al., 2021).

#### 2.1.1 Serine palmitoyltransferase

Serine palmitoyltransferase (SPT) is a resident enzyme of the endoplasmic reticulum (ER) (Aaltonen et al., 2022) which catalyzes the rate-limiting step in the *de novo* pathway (Wells and Lester, 1983; Hanada, 2003; Breslow and Weissman, 2010). SPT belongs to the α-oxoamine synthase family, enzymes that are responsible for the condensation of carboxylic acid CoA thioesters with amino acids (Gault et al., 2010). In this step, sphingoid or long-chain bases are formed by the decarboxylation of the amino acid serine and its condensation with palmitoyl-CoA. Sphingoid bases are the backbone of every complex sphingolipid. Although complex sphingolipids represent a diverse family of lipids, the diversity among sphingoid bases is quite restrictive. Broadly they can be grouped into three with decreasing abundance in mammalian cells: sphingosine, sphinganine, and phytosphingosine, which is commonly found in plants, yeasts, and some specialized tissues such as skin in humans (Chung et al., 2001; Mashima et al., 2019; Nădăban et al., 2022).

SPT is widely conserved across eukaryotes (Han et al., 2009), and certain prokaryotes contain this enzyme (Ikushiro et al., 2003). Bacterial SPT exists as a homodimer (Yard et al., 2007), while eukaryotic SPT is a heterodimer composed of two major subunits, SPT long-chain subunits 1 and 2 (SPTLC1 and SPTLC2). SPTLC1 is highly conserved throughout eukaryotes and exhibits significant sequence similarity to the bacterial SPT subunit. Conversely, SPTLC2 displays a relatively higher degree of sequence diversity and exists in two isoforms, namely SPTLC2 and SPTLC3. Either of these subunits can interact with SPTLC1 to form functional SPT enzymes with catalytic activity (Wang et al., 2021a). The SPTLC2 subunit, containing a binding site for pyridoxal 5-phosphate, is regarded as the catalytic subunit within the SPT enzyme (Hornemann et al., 2006). Recent findings have established that eukaryotic heterodimeric SPT relies on additional regulatory subunits such as the small subunit of SPT (ssSPT) (Han et al., 2009) and Orm/ORMDLs to achieve optimal activity and regulation (Davis et al., 2018).

Multiple isoforms of ssSPTs are present in eukaryotes. In humans, ssSPT exists in two isoforms ssSPTa and ssSPTb. Each of these isoforms can bind to the SPT heterodimer and boost its catalytic activity several hundred folds and confer acyl-CoA substrate chain length specificity introducing early diversity in sphingoid base chain lengths. It has been reported in mice and in plants (*A. thaliana*) that ssSPTa knockout mutants are embryonically lethal suggesting their importance in development (Kimberlin et al., 2013).

Orm proteins were first identified in yeast as negative regulators of *de novo* sphingolipid biosynthesis (Breslow et al., 2010). This family of proteins is highly conserved and homologs of yeast Orm proteins were identified in higher eukaryotes. In humans, these proteins are known as ORMDL and they exist in different isoforms ORMDL1, ORMDL2, and ORMDL3 and share around 80% sequence homology among themselves (Clarke et al., 2019). Any of these ORMDL isoforms can interact with SPT (Clarke et al., 2019; Davis et al., 2019; Wang et al., 2021a; Green et al., 2021). Thus, the SPT holoenzyme (Wattenberg, 2021) may consist of heterodimeric SPT (SPTLC1-2) along with ssSPT and ORMDL proteins. In short, the holoenzyme, known as the SPOTs complex, is a complex isoform capable of responding to sphingolipid levels in the intracellular milieu (Davis et al., 2019).

Since SPT is the rate-limiting step of sphingolipid biosynthesis, it is a major target for regulation. These regulatory subunits are crucial for mediating such regulation. SPT can exist in multiple isoforms (SPTLC1-SPTLC2, SPTLC1-SPTLC3 along with different combinations which include two ssSPT and three ORMDL isoforms, each with distinct enzymatic activity and acyl-CoA substrate preference. Recent determination of the molecular structure of SPTLC1-SPTLC2-ssSPTa isoform and SPTLC1-SPTLC2-ssSPTa-ORMDL3 complex shed light into the structural organization of SPT, substrate recognition, and mechanisms by which regulatory subunits of SPT (ssSPTs and ORMDLs) influence enzyme activity (Li et al., 2021a; Wang et al., 2021a). The phosphorylation status of different SPT subunits modulates the holoenzyme activity. Mutational studies with phosphomimetic and phosphodeficient mutants of SPTLC2 (at serine 384) demonstrated altered enzymatic activity in humans (Ernst et al., 2015). Phosphorylation of tyrosine 164 in SPTLC1 influences enzyme activity (Taouji et al., 2013). In plants (*Arabidopsis*), the phosphorylation of its long-chain base subunit stimulated both SPT activity and sphingolipid generation through the *de novo* pathway (Li et al., 2023). S1P, a metabolic intermediate, and its interaction with its receptor (S1PR) was reported to allow the degradation of ORMDLs, thus releasing the inhibitory effect on enzyme activity (Sasset et al., 2023). It was also shown that ER stress causes the upregulation of SPTLC2 (Kim et al., 2022a).

Loss/gain of function mutations or overexpression of these regulatory subunits can lead to pathophysiological conditions including diverse growth and developmental defects. It has been reported that functional deletion of ORMDL genes results in major myelinating defects in mice (Clarke et al., 2019). Consistent with ORMDL deletion, overexpression of catalytically super active isoform of SPT phenocopied myelination defects in mice. Additionally in mice, a mutation in ssSPTb with an increased affinity towards C_18_-fatty acyl-CoA was reported to cause neurodegeneration in the brain and in the eye (Zhao et al., 2015). Pathogenic ssSPTa variants have been reported to be involved in hereditary spastic paraplegia (Srivastava et al., 2023). SPT holoenzyme harboring these mutants can no longer be regulated by ORMDL-dependent inhibition, resulting in excessive sphingolipid production. Pathogenic SPTLC1 variants have been reported to cause childhood onset of amyotrophic lateral sclerosis, a debilitating progressive neurodegenerative disease (Mohassel et al., 2021; Lone et al., 2022). Another neurogenerative disease known as hereditary sensory neuropathy type 1 has been mapped to be in the SPTLC1 gene (Bejaoui et al., 2001). These observations suggest that SPT subunits are crucial for proper growth and development as well as in maintaining normal physiology.

Neurons (especially myelin sheath) are highly enriched in sphingolipids, and sphingolipid requirement is high during their development. Therefore, dysregulation in the enzyme which catalyzes the first and committed step in the *de novo* sphingolipid synthesis will have a great impact. This is in accordance with the fact that mutations in these subunits can be correlated with physiological and developmental defects. However, it will be misleading to interpret that mutations in SPT subunits can only cause neurological defects. Indeed, genetic and pharmacological inhibition of SPT resulted in profound changes in multiple organs in model organisms and humans. For example, SPT in mice was implicated in insulin sensitivity and adipose morphology (Chaurasia et al., 2016; Alexaki et al., 2017). Recently, SPTLC1 has also been reported to be critical for vascular development in mice (Kuo et al., 2022). Knockout studies in mice showed that SPTLC2 is required for blood pressure homeostasis (Cantalupo et al., 2020). A pathogenic variant of SPTLC1 has been linked to hyperkeratosis lenticularis perstans, a skin disorder (Jagle et al., 2023). Mutations in different SPT subunits have been implicated in clear cell renal cell carcinoma where the expression of SPTLC1 was found to be lower compared to normal kidney tissues (Zhu et al., 2020). ssSPTb-mediated modulation of SPT activity has been implicated in poor prognosis in prostate cancers (Costa-Pinheiro et al., 2020). Moreover, genome-wide association studies implicated ORMDL3 in asthma in humans (Moffatt et al., 2007). Multiple organs and systems in mammals are hence dependent on the proper functioning of SPT.

#### 2.1.2 3-Ketosphinganine reductase

The ER enzyme 3-ketosphinganine reductase (KDSR) forms sphinganine via a reduction of the ketone group of 3-ketosphinganine. KDSR is classified within a larger group of reductase enzymes referred to as short-chain dehydrogenase/reductase (Kihara and Igarashi, 2004). Initially discovered in yeast, KDSR was formerly recognized as TSC10 (Gupta et al., 2009). Homologs of TSC10 in mammals are called follicular variant translocation protein 1 (FVT1). It has been documented that the expression of human FVT1 in yeast *tsc10* knockout mutants can restore their functionality (Kihara and Igarashi, 2004).

Several mutations in KDSR have been reported to have a wide array of pathologic outcomes. A missense mutation in FVT1 has been linked to bovine spinal muscular atrophy, a recessive neurodegenerative disease (Krebs et al., 2007). Other mutations in this gene have been implicated in normal skin development (Boyden et al., 2017), thrombocytopenia (Bariana et al., 2019; Liu et al., 2020), and progressive keratodermia (Wu et al., 2022) in human patients. Additional compound heterozygous mutations in KDSR with a corresponding presence of unusual keto-type sphingoid base have been reported in patients born with harlequin ichthyosis (Pilz et al., 2022). Loss of function missense mutation in KDSR resulted in the progression of hepatomegaly and steatosis in zebrafish (Park et al., 2019). This finding is interesting because KDSR in zebrafish shows a high degree of homology with the human enzyme.

#### 2.1.3 Ceramide synthase

The addition of an acyl group to sphinganine is catalyzed by a family of 6 mammalian isozymes, the Cer synthase (CerS) family. In this process of *N*-acylation, fatty acids of various lengths are condensed with sphinganine, ultimately resulting in the production of dhCer of varying chain lengths (Lahiri and Futerman, 2005; Pewzner-Jung et al., 2006; Stiban et al., 2010; Zelnik et al., 2019). These CerS enzymes are located within the cytosolic layer of the ER (Mesika et al., 2007). It is noteworthy that almost all eukaryotes possess these enzymes, although their amino acid sequences exhibit significant variability (Levy and Futerman, 2010). The initial discovery of CerS occurred in the yeast *S. cerevisiae* during a functional screening aimed at identifying genes associated with aging (Schorling et al., 2001). Notably, the deletion of these genes led to an extended lifespan, leading to their initial classification as longevity-assurance genes (DMello N et al., 1994). Additionally, other CerS orthologs in higher eukaryotes were identified through homology searches and screening of respective yeast libraries. In more recent times, a comprehensive Cer synthetic pathway, including bacterial CerS, has been revealed in bacteria through the utilization of genomic and biochemical approaches (Stankeviciute et al., 2022). In humans, CerS enzymes exist in 6 different isoforms (CerS1-6). Each isoform displays a distinct but overlapping acyl-CoA chain length specificity and distinct tissue-specific expressions (Table 1) (Laviad et al., 2008; Levy and Futerman, 2010; Stiban et al., 2010).

**TABLE 1:** Different CerSs, their preferred substrates, their tissue distribution, and the key biological processes impacted by the ceramide product.

Isozyme	Preferred acyl chain-length	Highest tissue distribution	Key biological function(s) of the ceramide product
CerS1	C_18_	Brain, small intestine, testis, placenta, smooth muscle	Apoptosis, mitophagy, decreasing mitochondrial respiration
CerS2	C_22-26_	Brain, endocrine tissues, respiratory system, proximal digestive tract, liver & gallbladder, kidney, muscle	Skin, regulation of apoptosis
CerS3	C_22-26_ (and longer)	Proximal digestive tract, male reproductive tissues, skin	Skin barrier, membrane rigidness
CerS4	C_18-20_	Brain, endocrine tissues, respiratory system, gastrointestinal tract, liver, pancreas, male tissues, female tissues, skin	Apoptosis, membrane dynamics
CerS5	C_16_	Brain, endocrine tissues, proximal digestive tract, gastrointestinal tract, kidney, male tissue, skin, bone marrow & lymphoid tissues	Apoptosis, mitophagy, decreasing mitochondrial respiration
CerS6	C_14-16_	Brain, endocrine tissues, gastrointestinal tract, female tissues, muscle tissues, bone marrow and lymphoid tissues	Apoptosis, mitophagy

Over the years, a growing body of evidence showed that Cer levels are highly and tightly regulated. How individual CerS enzymes are regulated is yet to be fully understood. It appears that these genes are regulated at different levels, including transcriptional regulation (Pani et al., 2021), epigenetic modifications (Meyers-Needham et al., 2012), changes in enzymatic activity, posttranslational modification such as proteolytic degradation of CerS1 (Sridevi et al., 2009; Sridevi et al., 2010), and phosphorylation (Muir et al., 2014; Fresques et al., 2015; Sassa et al., 2016; Zhang et al., 2021a). Phosphorylation of several CerS isoforms (CerS2, CerS4, CerS5, and CerS6) by casein kinase 2 (CK2) was shown to enhance enzymatic activities (Sassa et al., 2016). Moreover, modulation of CerS enzymatic activities have shown to be dependent on their dimerization status specifically in CerS2, CerS5, and CerS6 (Laviad et al., 2012). CerS2 which typically produces C_24_ sphingolipids was shown to be co-regulated with Elovl1 (an isoform of the elongase family of enzymes that produces very long chain fatty acids) (Ohno et al., 2010). This is in accordance with the fact that in mouse liver CerS2 was shown to interact with fatty acid transporter protein 2 (Kim et al., 2022b). Additionally, a poorly characterized protein termed FAM57B was shown to stimulate CerS activity in human neurons and zebrafish brains (Tomasello et al., 2022). Recently, it was demonstrated that CerS1 is specifically inhibited by the small heat shock protein Hsp27 (Boyd et al., 2023).

In yeast, CerS isozymes Lag1 and Lac1 require an additional small 17 kDa protein known as Lip for optimal activity since downregulating Lip through promoter substitution led to a remarkable decrease in sphingolipid biosynthesis (Ishino et al., 2022). Surprisingly, no mammalian homologs of Lip1 were found to be required for CerS activity (Vallee and Riezman, 2005). Optimal CerS activity in yeast is dependent on CK2-mediated phosphorylation of Lag1 and Lac1 (Fresques et al., 2015). Sphingolipid metabolism in yeast is sensed through TOR complex 2 through the detection of complex sphingolipid levels at the plasma membrane. Activation of TOR complex 2, results in CerS phosphorylation and enhanced activity in yeast by regulating kinases Ypk1 and Ypk2 (Aronova et al., 2008). Lag1 was hyperphosphorylated via TOR complex 2 and Ypk1 in Lip-deficient yeast (Ishino et al., 2022), indicating the presence of an intricate, finely-tuned mechanism of CerS regulation. Such regulation of yeast Lag1 and Lac1 by CK2 and TOR complex 2 is reminiscent of CK2-dependent phosphorylation of mammalian CerSs. These findings indicate the possible existence of an evolutionary conserved regulatory mechanism among eukaryotes.

Other stressors such as DNA damage, p53 upregulation result in increased Cer levels which can be inhibited by the specific CerS inhibitor fumonisin B1 suggesting upregulation of CerS (Panjarian et al., 2008), however, it is unclear whether such increases in Cer are due to increased expression of the biosynthetic enzyme or enhanced enzymatic activity. Treatment of “Compound C” an inhibitor of AMP-activated protein kinase, can upregulate both CerS5 expression and its enzymatic activity (Jin et al., 2009). Leptin signaling has also been implicated in altering transcript levels of CerS2 and CerS4 in addition to SPT (Bonzón-Kulichenko et al., 2009). These interactions indicate that close physical proximity of different proteins with different CerSs is specific and may even be required for optimal activity, substrate availability, and regulation. Each of these interactions can be a result or a target of yet-to-be-known broader regulatory mechanisms. Moreover, alternative splicing of CerS2, which excludes exon-8 containing a putative catalytic domain, showed reduced very long-chain Cer. Such reduction in Cer levels leads to increased proliferation and migration in certain breast cancer cells (Pani et al., 2021). A decrease in C_18_-Cer due to the repression of CerS1 expression has been linked to resistance to chemotherapy and metastasis. In head and neck squamous cell carcinoma a splice variant of CerS1 has been reported to be enriched. This splice variant lacks the region targeted by miR-574-5p for the micro-RNA-dependent degradation (Meyers-Needham et al., 2012). Characterization of CerS2 knockout mice showed a robust depletion in very long acyl chain Cer (C_22-24_). Nevertheless, C_16_-Cer levels were elevated suggesting the existence of a homeostatic compensatory regulatory mechanism (Pewzner-Jung et al., 2010).

Altered Cer levels are detected in several pathophysiological conditions including cancer (Brachtendorf et al., 2019), diabetes (Hammad et al., 2022; Hammad and Lopes-Virella, 2023), obesity (Raichur et al., 2019), Parkinson’s disease (Abbott et al., 2014), and other neurological disorders (Tomasello et al., 2022). Sphingolipids are also implicated in insulin resistance in the brain which is linked to metabolic and neurodegenerative diseases (Mei et al., 2023). *Postmortem* brain tissues of the anterior cingulate cortex in patients with Parkinson’s disease showed elevated levels of shorter chain-length sphingolipid species and corresponding elevation of CerS1 transcript (Abbott et al., 2014). The brain of homozygous CerS1 knockout mice showed aberrant cerebellum size and neuronal apoptosis (Ginkel et al., 2012). Mutation in Hsp27 has been implicated in a variant of Charcot-Marie-Tooth disease, an inherited neurological disorder. It has been shown that Hsp27 knockout mice have decreased Cer levels in peripheral neurons and that the protein colocalizes with CerS1. Loss of CerS1 localization due to Hsp27 S135F mutation shows a profound effect on mitochondrial morphology and respiratory function indicating a possible role of Hsp27 on the regulation of CerS1 (Schwartz et al., 2018). Homozygous deletion of CerS1 or CerS5 showed their requirement in maintaining skeletal muscle health (Tosetti et al., 2020).

Several CerS knockout mice have been generated by different groups to help study the biology of these enzymes. CerS2 knockout mice showed several phenotypic characteristics including aberrant membrane morphology, membrane order, and higher membrane fluidity (Pewzner-Jung et al., 2010; Silva et al., 2012). Another study reported a positive correlation between T cell activation and expression of CerS2 (Shin et al., 2021). In the liver, haploinsufficiency of CerS2 led to diet-induced steatohepatitis and insulin resistance (Raichur et al., 2014). Changes in the sphingolipid profile due to the deletion of CerS2 inhibit the packaging of human immunodeficiency virus envelop protein into virus particles (Barklis et al., 2021). Very long-chain-containing sphingolipids are required during spermatogenesis. CerS3 has been shown to be involved in this process where its expression is upregulated several hundred folds (Rabionet et al., 2008). CerS4 knockout mice were shown to be highly sensitive to azoxymethane/dextran sodium sulfate-induced colitis model (El-Hindi et al., 2022). Similarly, the ablation of CerS2 exacerbates dextran sodium sulfate-induced colitis (Kim et al., 2017). Interestingly, CerS6 knockout mice showed impaired neuromotor functions (Ebel et al., 2013).

Chemical and biological downregulation of certain CerS contributes to the progression or reversal of diseases. Metastasis-prone sublines of SKOV3 ovarian cancer cells had decreased CerS2 expression and a concomitant reduction in Cer concentrations. Downregulation of CerS2 induced metastasis in ovarian cancer whereas knock-in of CerS2 suppressed the formation of lamellipodia required for cancer cell motility (Zhang et al., 2021b). CerS6 knockdown by antisense RNA was shown to induce the progression of breast and pancreatic cancers (Gao et al., 2022; Pan et al., 2022). On the other hand, graft-versus-host-disease was prevented and reversed by genetic or pharmacologic inhibition of CerS6 (Sofi et al., 2022). The synthesis of epidermal Cer species via CerS2 and CerS3 improves the barrier function of some skin diseases, such as atopic dermatitis, and ichthyosis (Shin et al., 2020). Interestingly, in another study, CerS4 was shown to be required for epidermal barrier function, as its deficiency results in impaired epidermal lipid metabolism and adult epidermal barrier function (Peters et al., 2020). Both mRNA and CerS3 protein levels were elevated in hepatocellular carcinoma compared with adjacent tissues in human patients. Higher CerS3 expression correlated with shorter patient survival. *In vivo*, Hep3B overexpressing CerS3 demonstrated increased cell viability and proliferation, as well as enhanced migration and invasion (Cai et al., 2022). These results highlight the role of CerS3 in cancer progression, possibly through the production of very long chain Cer species. A possible mechanism is due to the fact that Cer of different chain lengths interfere with other Cer ability to permeabilize mitochondrial membranes to initiate apoptosis (Stiban and Perera, 2015).

#### 2.1.4 Dihydroceramide desaturase

The fourth and final step in the *de novo* biosynthetic pathway involves the oxidation of dhCer at the C4 position of its sphingoid backbone resulting in a 4,5-trans double bond in the molecule. This is catalyzed by the ER enzyme dhCer desaturase (DEGS/DES) and it results in the production of Cer (Geeraert et al., 1997; Michel et al., 1997). DEGS introduces. Two distinct isoforms of DEGS exist in humans, DEGS1 and DEGS2 (Ternes et al., 2002). These isoforms differ not only in their catalytic functions but also in their tissue-specific expressions (Endo et al., 1996; Ternes et al., 2002; Omae et al., 2004; Siddique et al., 2012). DEGS1 primarily demonstrates Δ4-desaturase activity, whereas DEGS2 exhibits both Δ4-desaturase and C4 monooxygenase activities (Ternes et al., 2002; Rodriguez-Cuenca et al., 2015).

An increasing body of reports suggests that DEGS is regulated transcriptionally as well as by other regulators (Rodriguez-Cuenca et al., 2015). Oxidative stress and hypoxia have been reported to inhibit DEGS activity (Idkowiak-Baldys et al., 2010). Transcription factors such as NFATC, Hand2 were reported to be directly involved in low transcript levels of DEGS1 in chronic hypoxia of cardiomyocyte (Azzam et al., 2013). Other factors such as fatty acids and cytokines are also known to regulate dhCer levels by modulating the expression of DEGS (Dogra et al., 2007; Dybkaer et al., 2007). Homozygous deletion of DEGS1 results in several physiological defects including scaly skin formation, smaller size, and tremors, and confers insulin sensitivity in mice (Holland et al., 2007). Mouse embryonic fibroblasts lacking DEGS1 alleles were shown to confer protection from apoptosis induced by the chemotherapeutic agent etoposide (Siddique et al., 2012). Until recently, human mutations in DEGS were not reported. In 2019, a report identified a DEGS1 variant causes a complex neurological disorder in four consanguineous individuals. The patients suffered mild to severe intellectual disability, spastic quadriplegia, scoliosis, and epilepsy (Dolgin et al., 2019). This neuronal dysfunction and neurodegeneration caused by mutations in DEGS1 highlights the importance of this enzyme in maintaining neuronal function. DEGS1 was also shown to regulate the compartmentalization of Rac1. Rac1 is a small GTPase that regulates oxidative stress and plays a crucial role in the pathogenesis of various neurological diseases. DEGS1 deficiency causes dhCer buildup and an ensuing Rac1 mislocalization in the cell, which in turn enhances oxidative stress and causes neurodegeneration (Tzou et al., 2021).

Recent studies have shown that DEGS dysregulation is implicated in the pathogenesis of several diseases, including metabolic diseases, endothelial impairment, neurological disorders, and Alzheimer’s disease. DEGS drives lipotoxicity in metabolic diseases (Blitzer et al., 2020). Lipotoxicity refers to the accumulation of lipids in non-adipose tissues, which leads to cellular dysfunction, insulin resistance, and inflammation. It was recently demonstrated that DEGS1 inhibition can alleviate endothelial impairment induced by indoxyl sulfate, a uremic toxin that accumulates in chronic kidney disease. Indoxyl sulfate disrupts endothelial function and contributes to the development of cardiovascular disease. DEGS1 inhibition can restore endothelial function and reduce oxidative stress in endothelial cells (Savira et al., 2021). Moreover, DEGS1 inhibition can reduce amyloid-β levels in primary neurons obtained from an Alzheimer’s disease transgenic model (Ordóñez-Gutiérrez et al., 2018).

### 2.2 Catabolism of complex sphingolipids

Degradation of complex sphingolipids occurs in the lysosome, leading to the generation of Cer (Liu et al., 1997). This process is comprised of a series of enzymes that are aided by activator protein chaperones (Linke et al., 2001). Multiple enzymes are involved in the lysosomal breakdown of sphingolipids, and the deficiency of any of these enzymes can give rise to severe genetic disorders known as lysosomal storage diseases (LSDs) (Platt, 2014). Sphingolipidoses are a subset of LSDs primarily associated with sphingolipid metabolism (Abed Rabbo et al., 2021).

#### 2.2.1 Sphingomyelin hydrolysis

Sphingomyelinases (SMases) hydrolyze SM producing Cer and phosphocholine (Chatterjee, 1999). They can be categorized based on their pH preferences. Acidic and neutral SMases (aSMase and nSMase) are primarily found in lysosomes and cell membranes, while alkaline SMase (alk-SMase) is predominantly present in the gastrointestinal tract (Duan, 2006; Insausti-Urkia et al., 2020). These enzymes play roles in apoptosis, inflammation, and immune response, and their dysregulation has been linked to diseases such as Niemann-Pick disease and neurodegenerative disorders like Alzheimer’s disease (Yatsu, 1971; Schulze and Sandhoff, 2011; Bienias et al., 2016; Moll et al., 2021).

##### 2.2.1.1 Acid sphingomyelinase

Acid SMase (aSMase) is a lysosomal metalloenzyme that exhibits optimal activity at pH 4.5-5. The enzyme contains several Zn^2+^-binding motifs, which makes Zn2+ essential for its activity (Breiden and Sandhoff, 2021). Numerous *in vitro* studies have demonstrated that aSMase is activated by dimerization following direct oxidation (Henry et al., 2013). However, it is still unknown if aSMase is oxidatively activated *in vivo*, as several *in vivo* studies have reported that free oxygen radicals do not have an activating effect on aSMases (Gulbins and Petrache, 2013). aSMases are divided into two types, lysosomal SMase and secretory SMase (Jenkins et al., 2009). Lysosomal SMase is primarily considered an enzyme that catalyzes SM hydrolysis in lysosomes. Moreover, aSMase is found on the plasma membrane because the lysosomal membrane is constantly transported to the cell surface through the endomembrane system. At the plasma membrane, aSMase produces surface Cer and functions as a signaling protein (Kornhuber et al., 2022). The specific roles of lysosomal SMase are still not well understood, but it is thought to be associated with Cer-signaling pathways such as apoptosis. However, deficiency in this enzyme leads to defects in cellular processes (Jenkins et al., 2009). On the other hand, secretory aSMase is secreted extracellularly by the Golgi apparatus. Secretory aSMase has a putative role in many physiological and pathophysiological processes, such as atherosclerosis, where it may be involved in the aggregation of lipoproteins (Tabas, 1999). The significance of aSMase makes its mutation a cause of many LSDs. aSMase deficiency results in various degrees of SM buildup. Lipid storage causes foam cell infiltration in tissues, as well as clinical symptoms such as hepatosplenomegaly, pulmonary failure, and, in some cases, central nervous system involvement (McGovern et al., 2017; Pinto et al., 2021). The SMPD1 gene encodes aSMase. Recessive mutations in this gene lead to aSMase deficiency, resulting in the accumulation of SM and causing LSDs known as Niemann-Pick disease type A and B (Andrews, 2019).

##### 2.2.1.2 Neutral sphingomyelinase

nSMase, with an optimal pH of 7.4, is an enzyme that can be activated by a range of structurally diverse compounds that are physiologically relevant, including tumor necrosis factor (TNFα) (Marchesini and Hannun, 2004; Shamseddine et al., 2015). This enzyme was discovered in tissues from Niemann-Pick disease patients. There are four distinct nSMases found in mammals, including nSMase1-3 and mitochondria-associated nSMase (Sindhu et al., 2021). All isoforms require a neutral pH and divalent cations (Mg^2+^/Mn^2+^) for activity (Sindhu et al., 2021).

nSMase1 is a membrane protein with two putative transmembrane domains at its C-terminus. *In vitro* nSMase activity analysis demonstrated Mg^2+^-dependence. Various research has shown that nSMase1 is critical for Cer production in response to stress, and the Cer produced may be a key component of T cell receptor signaling (Tonnetti et al., 1999).

nSMase2, which has been shown to be palmitoylated in two cysteine clusters, has optimal activity at neutral pH, is magnesium-dependent, and is stimulated by unsaturated fatty acids and anionic phospholipids, particularly cardiolipin and phosphatidylserine (Tani and Hannun, 2007). It is a key player in stress-induced Cer formation, and several anti-cancer drugs have been shown to inhibit nSMase2 (Clarke and Hannun, 2006; Shamseddine et al., 2015).

nSMase3 is a C-tail-anchored integral membrane protein with an ER signal in the C-terminus and a proline-rich domain at the N-terminus that may be involved in protein-protein interactions. Research on its functional roles is limited compared to the other nSMases since it was only recently cloned (Corcoran et al., 2008; Shamseddine et al., 2015).

Studies have demonstrated that defects in the nSMase gene, the primary regulator of Cer biosynthesis via the hydrolysis pathway, are responsible for progressive bone defects (Kim et al., 2008a). Additionally, nSMase2 activation and Cer inhibit the insulin signaling pathway, preventing glucose transporter 4 translocation to the plasma membrane (Pralhada Rao et al., 2013; Sindhu et al., 2021). Furthermore, in obesity, chronic low-grade inflammation drives the production of proinflammatory cytokines, which may increase nSMase2 expression and activity, leading to Cer formation, mitochondrial dysfunction, and reactive oxygen species generation (Samad et al., 2006). Finally, nSMase is suggested to be involved in the pathogenesis of several other diseases, including atherosclerosis, inflammation, and heart failure (Adamy et al., 2007; Rajagopalan et al., 2015; Deng et al., 2020; Sindhu et al., 2021).

##### 2.2.1.3 Alkaline sphingomyelinase

Initially identified as an enzyme that catalyzes the breakdown of sphingomyelin (SM) via phospholipase C activity, alkaline SMase (alk-SMase) has a different sequence than acid and neutral SMases. Gene cloning revealed that alk-SMase shares similarities with members of the nucleotide pyrophosphatase/phosphodiesterase (NPP) family (Duan, 2006; Duan, 2018) and is therefore also known as NPP7. The enzymes of the NPP family are classified as ectoenzyme because they cross the plasma membrane through either the N or C terminal transmembrane domain, with the remainder of the enzyme located outside the cells (Duan, 2006; Gorelik et al., 2017). alk-SMase is expressed in the intestinal mucosa, as well as in the human liver and bile in many species (Cheng et al., 2007). Studies have shown that intestinal alk-SMase can suppress colonic carcinogenesis and inflammation, hydrolyze dietary SM, and increase cholesterol absorption. There are three mechanisms thought to be involved in alk-SMase anticancer actions. Firstly, it hydrolyzes SM to Cer, which is a well-known antiproliferative and apoptotic molecule. Secondly, it degrades the phosphocholine moiety of the platelet-activating factor, rendering it inactive. Platelet-activating factor is abundantly produced in inflammatory tissues, causing inflammation and carcinogenesis. Finally, NPP7 converts lyso-phosphatidylcholine to monoacylglycerol, limiting the generation of lysophosphatidic acid, which otherwise can be formed by NPP2, and can lead to cell lysis (Chen et al., 2015; Duan, 2018; Alyamani et al., 2023).

#### 2.2.2 Hydrolysis of glycosphingolipids

Glycosphingolipids (GSLs) have been associated with many neurological diseases and important cellular processes (Hakomori, 2003; Lopez and Schnaar, 2009; Lingwood, 2011; Schengrund, 2015; Breimer et al., 2017; Zhang et al., 2019; Komatsuya et al., 2020). Catabolism of GSLs is considered the second mode of generation of Cer via hydrolysis. GSLs get endocytosed and transported to the luminal face of intra-lysosomal vesicles, where they face consecutive removal of sugar residues using glycosyl hydrolases (glycosidases) (Kolter and Sandhoff, 2005). This catabolic process generates monosaccharides and Cer which is recycled and reinserted into the lysosomal membrane (Ryckman et al., 2020). GSLs with short sugar chains are not easily susceptible to water-soluble hydrolyzing enzymes and require the assistance of auxiliary lysosomal lipid binding proteins. GM2 activator protein (GM2AP) and four saposins are among these proteins (Kolter and Sandhoff, 2010; Quinville et al., 2021). Saposins disrupt the interaction of the glycolipid substrate with the local membrane environment and allow the glycans to be accessed by water-soluble hydrolytic enzymes. Consistent with the importance of activator proteins in GSLs catabolism, mutations in these activator proteins are strongly linked to pathological buildup of GSLs in certain LSDs despite the presence of the hydrolase responsible for degradation (Ryckman et al., 2020; Abed Rabbo et al., 2021).

*N*-acetyl-hexosaminidases (Hex A and Hex B) are glycosidases that cleave *N*-acetyl-glucosamine or *N*-acetyl-galactosamine residues from glycoconjugates in ganglioside catabolism. Hex A is capable of degrading negatively charged and uncharged substrates, while Hex B primarily cleaves off *N*-acetylgalactosamine residues from the uncharged GA2 and globotetraosylceramide (Lemieux et al., 2006). Hex A and GM2AP aid in the hydrolysis of GM2. GM2AP transports GM2 from the membranous environment to the soluble Hex A for hydrolysis (Zarghooni et al., 2004). The *N*-terminal and central hydrophobic domains of GM2AP bind to the negatively charged lysosomal membrane surface through multiple lysine residues, disrupting membrane structure and carrying GM2 out of the membrane into Hex A for hydrolysis (Anheuser et al., 2015). Hex A removes the terminal *N*-acetylgalactosamine from the GM2 ganglioside reducing it to GM3 (Lemieux et al., 2006; Tancini et al., 2012; Abed Rabbo et al., 2021; Quinville et al., 2021). GM3 is hydrolyzed by the removal of terminal sialic acid residues through the collaborative action of sialidase (α-*N*-acetyl neuraminidase) and saposin B, producing lactosylceramide (LacCer) (Khan and Sergi, 2018). LacCer is further hydrolyzed to GlcCer by GM1-β-galactosidase with saposin B or by galactosyl-ceramide-β-galactosidase with saposin C (Kolter and Sandhoff, 2005). GlcCer then produces Cer via the action of glucosylceramidase (Quinville et al., 2021).

##### 2.2.2.1 Glucosylceramidase

Glucosylceramidase is an enzyme that plays a pivotal role in the degradation of sphingolipids by hydrolyzing GlcCer, a key component of these complex lipids, to glucose and Cer. This enzymatic breakdown process is essential for maintaining cellular homeostasis and ensuring the proper functioning of various physiological processes. Through the action of glucosylceramidase, the recycling of sphingolipid constituents is facilitated, enabling the utilization of their building blocks for other cellular processes.

Disturbances in glucosylceramidase activity have been implicated in the pathogenesis of Gaucher disease, an LSD characterized by the accumulation of GlcCer and other sphingolipids in multiple tissues and organs (Futerman and Platt, 2017). Gaucher disease is primarily caused by reduced or absent glucosylceramidase activity, resulting in the impaired breakdown and clearance of GlcCer. This accumulation of sphingolipids has detrimental effects, particularly in the central nervous system, leading to severe neurological manifestations observed in affected individuals (Vitner and Futerman, 2013; Vitner et al., 2015).

##### 2.2.2.2 N-acetyl-hexosaminidase

Hex enzymes break down certain complex carbohydrates off GSLs. There are two types of Hex enzymes, Hex A and Hex B, which are encoded by the *HEXA* and *HEXB* genes, respectively (Hultberg et al., 1996). Hex A is a heterodimeric enzyme composed of an alpha subunit (encoded by *HEXA*) and a beta subunit (encoded by *HEXB*). This enzyme is mainly found in the lysosomes of nerve cells and is responsible for breaking down GM2 ganglioside (Lemieux et al., 2006). Deficiencies in Hex A activity can lead to the accumulation of GM2 ganglioside in the lysosomes of nerve cells, which can cause a group of inherited disorders known as GM2 gangliosidosis, including Tay-Sachs disease (Dersh et al., 2016). Hex B, on the other hand, is a homodimeric enzyme composed of two beta subunits (encoded by *HEXB*). It is found in lysosomes throughout the body and is involved in the breakdown of other GSLs besides GM2 ganglioside (Bateman et al., 2011).

Deficiencies in Hex A and Hex B can lead to the accumulation of specific GSLs in lysosomes, causing different LSDs (Abed Rabbo et al., 2021). Tay-Sachs disease is a severe form of GM2 gangliosidosis caused by a deficiency in Hex A activity. It is characterized by the accumulation of GM2 ganglioside in the lysosomes of nerve cells, which can cause progressive damage to the nervous system. Symptoms typically appear in infancy and can include muscle weakness, developmental delay, and blindness (Ramani and Parayil Sankaran, 2023). Moreover, Sandhoff disease is another type of GM2 gangliosidosis caused by a deficiency in both Hex A and Hex B activities. It is similar to Tay-Sachs disease in terms of symptoms and pathology, but it tends to be more severe and progresses more rapidly. Whereas GM1 gangliosidoses are a group of disorders caused by a deficiency in β-galactosidase, which is another lysosomal enzyme involved in the breakdown of GSLs. However, in some cases, GM1 gangliosidosis can also be caused by a deficiency in Hex B activity, which leads to the accumulation of GM1 ganglioside in the lysosomes of various tissues. Symptoms can vary widely depending on the type and severity of the disease and can include developmental delay, seizures, and muscle weakness (Regier et al., 2016; Rha et al., 2021). Deficiencies in Hex A and Hex B are not limited to these diseases, as they can also lead to the accumulation of other GSLs in lysosomes, which can cause other types of LSDs such as Krabbe disease, Fabry disease, and Niemann-Pick disease, among others (Patterson et al., 1993; Pavuluri et al., 2017; Lenders and Brand, 2021).

### 2.3 The salvage pathway

Cer is the central molecule of the sphingolipid family and it plays multifaceted roles in cellular response, growth, senescence, and differentiation (Saba et al., 1996; Bartke and Hannun, 2009). The salvage pathway of Cer (also known as the sphingolipid recycling pathway) is essentially a combination of different enzymes used in the other two pathways. It uses the CerS isozymes (from the *de novo* pathway) to *N*-acylate sphingosine directly producing Cer (Jin et al., 2008; Kitatani et al., 2008). Sphingosine is generated through the catabolism of complex sphingolipids such as GSLs and SM. Cer can be deacylated to sphingosine and fatty acid by ceramidases. Sphingosine can then be reacylated to produce Cer (Kitatani et al., 2008; Coant et al., 2017) using CerS.

#### 2.3.1 Ceramidase

Ceramidases are a family of hydrolytic enzymes that deacylate Cer to produce sphingosine and free fatty acid. Ceramidases are classified into five types based on the appropriate pH for their activity. Acid ceramidase (aCDase) is an enzyme that catalyzes the hydrolysis of unsaturated small and medium-chain ceramides (C_6_–C_18_). Neutral ceramidases (nCDase) use Cer and dhCer as substrates. They aid in the breakdown of dietary Cer molecules. nCDases are capable of catalyzing the hydrolysis of Cer species containing 16 and 18 carbons. Alkaline ceramidases (ACERs) are mainly found mainly in the Golgi apparatus and the ER, where they hydrolyze very long-chain Cer (C_20_–C_24_) (Coant et al., 2017; Quinville et al., 2021).

##### 2.3.1.1 Acid ceramidase

Acid ceramidase (aCDase) is a lysosomal enzyme that hydrolyzes small and medium-chain ceramides (C_6_–C_18_) through intralysosomal membrane turnover. The optimal pH for full activity is ∼4.5. AC is synthesized as inactive proenzymes and activated by autocleavage of an internal peptide bond preceding Cys 143. Saposin D facilitates aCDase access to Cer access by involving membrane disruption (Coant et al., 2017; Gebai et al., 2018). aCDase can also catalyze the reverse reaction and synthesize Cer from sphingosine and free fatty acids *in vitro* and *in situ* (Park and Schuchman, 2006).

Research on Farber disease has provided insights into the role of aCDase in human diseases. Farber disease is an LSD caused by a mutation in the *ASAH1* gene encoding aCDase, leading to decreased enzymatic activity and Cer accumulation in the tissues (Ehlert et al., 2007). Farber’s disease symptoms appear in children within the first few weeks of birth. It has a clinical triad of painful joint deformity, subcutaneous nodules near the joints, and hoarseness (Ekici et al., 2012). In addition, aCDase protein expression was found to be upregulated in Ulcerative Colitis tissues. Myeloid aCDase deficiency protects against tumor occurrence in colitis-associated cancer and limits the expansion of neutrophils and granulocytic myeloid-derived suppressor cells in the tumor microenvironment (Venable et al., 1994; Espaillat et al., 2018).

##### 2.3.1.2 Neutral ceramidase

Neutral ceramidase (nCDase), encoded by *ASAH2* gene, is a type II membrane protein involved in the metabolism of dietary sphingolipids and Cer molecules. nCDase is highly expressed in the large intestine and can hydrolyze Cer species containing 16 and 18 carbons (Garcia-Barros et al., 2016; Coant et al., 2018). The optimal pH required for its activity is around 7. It is localized to the plasma membrane, ER/Golgi complex, and mitochondria, particularly in the liver (Sakamoto et al., 2018). Inhibiting nCDase molecularly and pharmacologically in colon cancer cells increases Cer, which results in decreased cancer cell survival and increased apoptosis. nCDase inhibition also resulted in the loss of components of pathways involved in the development of colon cancer, including β-catenin and ERK inhibition (Garcia-Barros et al., 2016). nCDase knockout mice are resistant to acute kidney injury caused by cisplatin, a chemotherapeutic agent used to treat solid organ tumors. Cisplatin’s utility in treating certain cancers is limited due to its nephrotoxicity. Following cisplatin treatment, NC knockout mice have better kidney function, less injury and structural damage, lower rates of apoptosis, and less ER stress (Sears et al., 2022).

##### 2.3.1.3 Alkaline ceramidase

There exist three distinct subtypes of alkaline ceramidases (ACER1-3), which demonstrate optimal activity at pH ∼9. ACER1 is primarily located in the ER and selectively catalyzes the hydrolysis of Cer with unsaturated long acyl chains or very long saturated or unsaturated acyl chains. ACER2, on the other hand, is a ceramidase that predominantly resides in the Golgi apparatus and targets different Cer for hydrolysis. ACER3, however, is present in both the ER and Golgi complex and preferentially catalyzes the hydrolysis of phytoceramide, a Cer analog, as well as Cer with unsaturated long acyl chains (Xu et al., 2010). Using various biochemical and structural approaches the Hannun and Obeid groups found that ACER enzymes share a similar catalytic mechanism with other zinc-dependent amidases, such as carboxypeptidases and thermolysin. Specifically, the enzymes use a zinc ion to activate a water molecule, which then attacks the amide bond in the Cer substrate, leading to hydrolysis (Yi et al., 2022).

In terms of function, ACER1 regulates Cer metabolism, which is essential for maintaining epidermal and appendageal homeostasis. Studies in mice have shown that ACER1 deficiency leads to an increase in various Cer and sphingoid bases in the skin, eventually causing gradual hair loss due to an enlargement of the follicular infundibulum and a decrease in hair follicle stem cells (Lin et al., 2017).

ACER2, a transcriptional target of the p53 protein, regulates the expression of genes involved in cell cycle arrest, programmed cell death, and metabolism to facilitate the DNA damage response (Xu et al., 2018). Deficiency of the ACER2 gene in mice leads to reduced blood levels of sphingoid base 1-phosphates, such as S1P and dhS1P, by controlling the generation of sphingoid bases in hematopoietic cells (Li et al., 2018). ACER2 is also implicated in placental vascular integrity in mice. It is expressed in both maternal and fetal endothelial cells of the placenta and is essential for normal fetal growth and survival. Loss of ACER2 in either the mother or the fetus resulted in placental vascular defects, leading to fetal growth restriction, placental thrombosis, and fetal demise. ACER2 plays a critical role in maintaining sphingolipid homeostasis in the placenta, which is necessary for proper vascular function (Li et al., 2020). Moreover, ACER2 is upregulated in response to DNA damage and the loss of ACER2 or its bioactive product sphingosine impairs the DNA damage response and increases genomic instability (Xu et al., 2016).

ACER3, with its multiple transmembrane domains, is mainly located in the ER and Golgi membrane. ACER3 promotes the growth of hepatocellular carcinoma cells through the S1P/S1PR2/PI3K/AKT signaling pathway and may be a potential therapeutic target for the treatment of liver cancers (Yin et al., 2018). Knockdown of ACER3 has been shown to decrease cancer cell growth and increase apoptosis in hepatocellular carcinoma patients. In addition, ACER3 knockout in mice results in an accumulation of multiple Cer, as well as a lack of the age-related increase in sphingosine and S1P levels in the brain, leading to Purkinje cell degeneration and motor coordination deficits (Wang et al., 2015).

### 2.4 Inhibitors of sphingolipid metabolic enzymes

**FIGURE 3:**
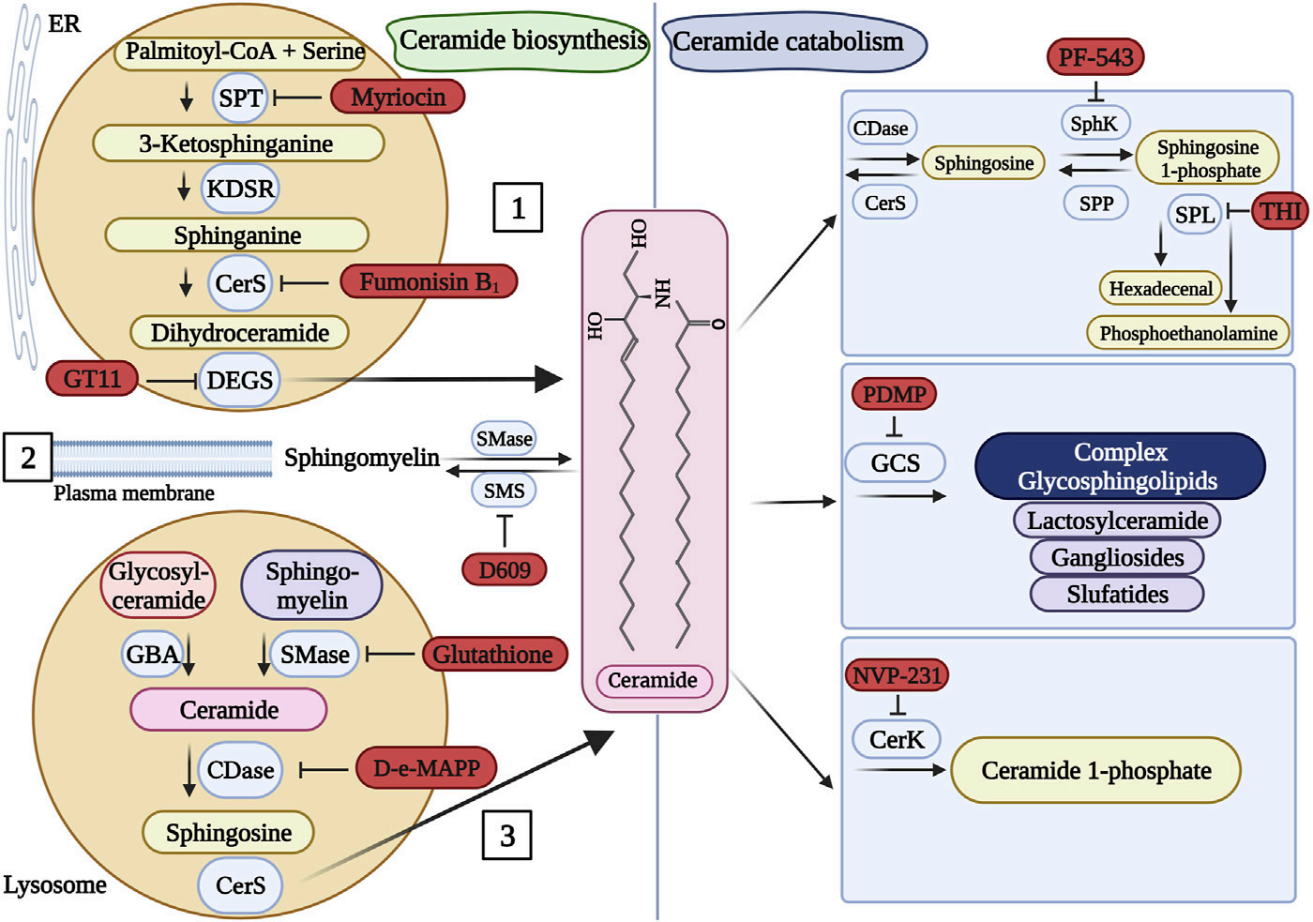
Pharmacological inhibitors of sphingolipid metabolism. Ceramide (the center molecule) is generated (left panel) via the *de novo* pathway [1], sphingomyelin hydrolysis [2], or the salvage pathway [3]. Ceramide can be catabolized (right panel) into several molecules. Known inhibitors of some enzymes of the pathways are presented in red. When more than one inhibitor is available for a particular enzyme, a random representative inhibitor was selected. SPT, serine palmitoyltransferase; KDSR, 3-ketosphinganine reductase; CerS, ceramide synthase; DEGS, dihydroceramide desaturase; SMase, sphingomyelinase; SMS, sphingomyelin synthase; GBA, glucocerebrosidase; CDase, ceramidase; SphK, sphingosine kinase; SPP, sphingosine 1-phosphate phosphatase; SPL, sphingosine 1-phosphate lyase; GCS, glucosylceramide synthase; CerK, ceramide kinase. This figure was created with Biorender.com using a paid student subscription.

As in other metabolic pathways, small, inhibitory molecules of sphingolipid enzymes are available (Millner and Atilla-Gokcumen, 2021; Skácel et al., 2021). These compounds selectively modulate the activity of specific enzymes involved in multiple sphingolipid metabolic pathways (Figure 3). The complex nature of sphingolipid pathways is also reflected in the variety of compounds that interact with, and inhibit, certain enzymes in the pathway. Table 2 below outlines some of the inhibitory molecules and their target enzymes. In the table, the molecules are classified as “inhibitors” if they exert direct effects on the enzyme they inhibit or “modulators” that affect the enzymes indirectly. These inhibitors, most of which are natural products, have been extensively used in basic research to study the complex intricate biology of sphingolipids and understand reaction mechanisms. More importantly, they hold significant promise for therapeutic interventions in a range of diseases, including cancer, LSDs, immune system dysfunctions, and neurodegenerative diseases (Bu et al., 2021; Afrin et al., 2023).

**TABLE 2:** Different inhibitors and modulators of sphingolipid metabolic enzymes.

Enzyme	Inhibitor/modulator	Classification	References
Serine palmitoyltransferase (SPT)	1. Myriocin	Inhibitor	Kluepfel et al. (1972), Osuchowski et al. (2004)
	2. Sphingofungin	Inhibitor	Hanada et al. (2000)
	3. Lipoxamycin	Inhibitor	Mandala et al. (1994), Harrison et al. (2018)
	4. _L_-cycloserine	Inhibitor	Lowther et al. (2010)
	5. β-chloro-_L_-alanine	Modulator	Ikushiro et al. (2004)
Ceramide synthase (CerS)	1. Fumonisin B1	Inhibitor	Harel and Futerman (1993), Merrill et al. (1993), Riley and Merrill (2019)
	2. Australifungin	Inhibitor	Mandala et al. (1995), Wells et al. (1998), Mandala and Harris (2000), Mullen et al. (2012)
	3. Fingolimod (FTY720)	Inhibitor	Lahiri et al. (2009)
Dihydroceramide desaturase (DEGS1)	1. GT11	Inhibitor	Triola et al. (2004), Skácel et al. (2021)
	2. XM462	Inhibitor	Camacho et al. (2012)
	3. Fenretinide	Inhibitor	Rahmaniyan et al. (2011), Bikman et al. (2012)
	4. 4-[4-(4-chloro-phenyl)-thiazol-2-ylamino]-phenol (SKI-II)	Inhibitor	Cingolani et al. (2014)
	5. Resveratrol	Modulator	Signorelli et al. (2009)
	6. Celecoxib	Inhibitor	Schiffmann et al. (2009), Siddique et al. (2015)
	7. Curcumin	Modulator	Fabrias et al. (2012), Casasampere et al. (2016)
	8. Δ^9^-tetrahydrocannabinol (THC)	Modulator	Hernández-Tiedra et al. (2016), Muñoz-Guardiola et al. (2021)
Ceramide kinase (CerK)	1. NVP-231	Inhibitor	Pastukhov et al. (2014)
Glucosylceramide synthase (GCS)	1. Eliglustat	Inhibitor	El Malki et al. (2023)
	2. Miglustat	Inhibitor	El Malki et al. (2023)
	3. T-036	Inhibitor	Fujii et al. (2021)
	4. 1-phenyl-2-decanoylamino-3-morpholino-1-propanol (PDMP)	Modulator	Inokuchi and Radin (1987)
	5. DL-threo-1-phenyl-2-palmitoylamino-3-morpholino-1-propanol (PPMP)	Modulator	Stefanić et al. (2010)
	6. *N*-butyldeoxynojirimycin (NBDNJ)	Inhibitor	Platt et al. (1994)
	7. *N*-butyldeoxygalactonojirimycin (NBDGJ)	Inhibitor	Andersson et al. (2000)
Ceramidase (CDase)	1. (*1S,2R*)-_D_-*erythro*-2-(*N*-myristoylamino)-1-phenyl-1-propanol (D-e-MAPP)	Modulator	Bielawska et al. (1996), Raisova et al. (2002)
	2. (*1R,2R*)-2-N-myristoylamino-1-(4-nitrophenyl)-1,3-propandiol (D-NMAPPD, and AD2646)	Inhibitor	Dagan et al. (2003), He et al. (2005)
	3. Ceranib-2	Inhibitor	Draper et al. (2011)
	4. SACLAC	Inhibitor	Pearson et al. (2020)
Sphingomyelinase (SMase)	1. Glutathione	Inhibitor	Liu and Hannun (1997)
	2. Phosphatidylinositol 3,5-bisphosphate (PIP_2_)	Inhibitor	Kolzer et al. (2003)
	3. Phosphatidylinositol 3,4,5-triphosphate (PIP_3_)	Inhibitor	Testai et al. (2004)
	4. Ceramide 1-phosphate (C1P)	Inhibitor	Gomez-Munoz et al. (2004)
	5. Sphingosine 1-phosphate (S1P)	Modulator	Gomez-Munoz et al. (2003)
	6. α-mangostin	Inhibitor	Okudaira et al. (2000)
	7. Cowanin	Inhibitor	Okudaira et al. (2000)
	8. Cowanol	Inhibitor	Okudaira et al. (2000)
	9. GW4869	Inhibitor	Luberto et al. (2002)
	10. SR33557	Inhibitor	Jaffrezou et al. (1991)
	11. Macquarimicin A	Selective inhibitor	Munakata et al. (2003)
	12. Undecylidene-aminoguanidine (C11AG)	Selective inhibitor	Amtmann et al. (2000)
	13. Scyphostatin	Inhibitor	Nara et al. (1999)
	14. Alutenusin	Inhibitor	Uchida et al. (1999)
	15. Chlorogentisylquinone	Specific irreversible inhibitor	Uchida et al. (2001)
	16. Manumycin A	Specific irreversible inhibitor	Arenz et al. (2001)
	17. Spiroepoxide	Specific irreversible inhibitor	Arenz and Giannis (2000)
	18. Sphingolactones	Selective irreversible inhibitors	Wascholowski and Giannis (2006)
Sphingosine kinase (SphK)	1. *N,N*-dimethylsphingosine (DMS)	Inhibitor	Nishiuma et al. (2008), Yura et al. (2020)
	2. _L_-*threo*-sphinganine (safingol)	Inhibitor	Buehrer and Bell (1992), Olivera et al. (1998)
	3. (*2R,3S,4E*)-*N*-methyl-5-(4′-pentylphenyl)-2-aminopent-4-ene-1,3-diol (SK1-I)	Specific inhibitor	Paugh et al. (2008), Kapitonov et al. (2009)
	4. 4-[4-(4-chloro-phenyl)-thiazol-2-ylamino]-phenol (SKI-II)	Inhibitor	French et al. (2006), Chiba et al. (2010)
	5. PF-543	Inhibitor	Schnute et al. (2012), Yi et al. (2023)
	6. Fingolimod (FTY720)	Inhibitor	Kahan (2004), Baumruker et al. (2007), Takabe et al. (2008), White et al. (2016)
	7. ABC294640 (Opaganib)	Inhibitor	French et al. (2010), Gao et al. (2012), Baker et al. (2013), Snider et al. (2013)
	8. MP-A08	Inhibitor	Pitman et al. (2015), Powell et al. (2017)
Sphingosine 1-phosphate lyase (SPL)	1. 2-acetyl-4-(tetrahydroxybutyl)imidazole (THI)	It is still unclear whether THI, LX2931, or LX2932 inhibit SPL directly or not	Ohtoyo et al. (2015)
	2. (E)-1-(4-((1R,2S,3R)-1,2,3,4-tetrahydroxybutyl)-1H-imidazol-2-yl)ethanone oxime (LX2931)		Bagdanoff et al. (2010)
	3. (1R,2S,3R)-1-(2-(isoxazol-3-yl)-1H-imidazol-4-yl)butane-1,2,3,4-tetraol (LX2932)		Bagdanoff et al. (2010)

While it is beyond the scope of this review to dissect all known inhibitors of sphingolipid metabolism, it is worthwhile to mention a few prominent examples. Myriocin, also known as thermozymocidin, is an inhibitor of SPT, the first and regulatory enzyme of the *de novo* pathway (Riley et al., 1999). Myriocin, originally isolated from the thermophilic fungus *Myriococcum albomyces*, is a naturally occurring non-proteinogenic amino acid (Kluepfel et al., 1972). Myriocin has been an invaluable pharmacological tool for exploring the role of *de novo* sphingolipid biosynthesis in myriad cellular functions. By inhibiting SPT, myriocin disrupts sphingolipid generation leading to varied sphingolipid profiles within cells. Myriocin, thus, has been used to investigate the impact of altered sphingolipid levels on cellular processes, including cellular signaling, immune cell function, apoptosis, autophagy, and membrane dynamics, to name a few (Scarlatti et al., 2003; Johnson et al., 2004; Rentz et al., 2005; Ordoñez et al., 2015; Guo et al., 2020; Yu et al., 2022). It has also been instrumental in understanding the roles of sphingolipids in various diseases, such as cancer, cardiovascular diseases, and neurodegenerative disorders (Park et al., 2008; Adachi et al., 2018; Mingione et al., 2021; Weiss et al., 2021). While there may be several potential clinical applications for the use of myriocin, particularly in the context of diseases characterized by sphingolipid metabolism dysregulation, no approved drug by the U.S. Food and Drug Administration (FDA) is available to date. Nevertheless, it was shown that the suppression of SPT via the administration of myriocin in mouse models reduced the replication of hepatitis C virus (HCV) (Umehara et al., 2006). Similarly, myriocin inhibited HCV replication in human hepatocytes (Katsume et al., 2013) suggesting it might serve as a novel drug in the treatment of HCV infection. Myriocin has also shown potential in managing nonalcoholic steatohepatitis (Yang et al., 2019).

CerS enzymes are responsible for the loading of the long-chain base with the fatty acid generating a two-tailed molecule, dhCer. CerS enzymes are regulated at multiple levels. Pharmacologically, a natural product has been used as a potent inhibitor of these enzymes. Fumonisin B1 is a mycotoxin primarily produced by several species of *Fusarium* molds (Rentz et al., 2005). Since it inhibits CerS, it prevents the generation of pro-apoptotic lipids and hence is considered a potent carcinogenic particularly causing esophageal cancer and hepatocellular carcinoma (Stockmann-Juvala and Savolainen, 2008). Fumonisin B1 has been used in basic research to study the intricate mechanistic details of the *de novo* synthesis pathway in several organisms (Zeng et al., 2020; Li et al., 2021b; Chen et al., 2021; Zhao et al., 2022).

At the other end of sphingolipid metabolism, inhibitors such as eliglustat and miglustat represent important pharmacological compounds that target LSDs (Desnick et al., 2013; Garbade et al., 2020). These disorders are resultant of rare genetic conditions whereby certain lysosomal enzymes are defective or lacking leading to the accumulation of undegraded lipids within lysosomes (Abed Rabbo et al., 2021). Both drugs are inhibitors of GlcCer synthase (GCS) which is responsible for the glycosylation of Cer in the Golgi apparatus (Bleicher and Cabot, 2002; Pothukuchi et al., 2021). These drugs are FDA-approved for the treatment of Gaucher disease, the major LSD (Futerman and Platt, 2017). Miglustat (brand name Zavesca^®^), is approved for the treatment of Gaucher disease type 1 resulting from a deficiency in glucocerebrosidase. Miglustat thus mitigates Gaucher debilitating symptoms by limiting the buildup of GlcCer, reducing the storage of this substrate (Ficicioglu, 2008). While miglustat has shown promise in treating other LSDs, its FDA approval specifically pertains to Gaucher disease. A more recently approved oral drug for Gaucher disease is eliglustat (Cerdelga^®^) which is another inhibitor of GCS. While both inhibitors target GCS and concomitantly lower GlcCer concentrations, they are not equivalent. Miglustat is a glucose analog whereas eliglustat, which is more potent and specific, is a Cer mimic (Mistry et al., 2023). Both drugs use substrate reduction therapy which may complement enzyme replacement therapy in the treatment of LSDs, offering hope for improved therapeutic outcomes and enhanced quality of life for patients affected by such ailments (Abed Rabbo et al., 2021).

Another branch of sphingolipid metabolism is the phosphorylation of sphingosine to S1P by the sphingosine kinases (SphK1 and SphK2). S1P is a potent cell survival signal (see below). While FDA-approved SphK inhibitors are still lacking, multiple research compounds and experimental drugs are currently being investigated (Table 2). These molecules modulate S1P levels and cellular signaling resulting in some therapeutic outcomes. PF-543, developed by Pfizer, is currently considered the most potent and selective SphK1 inhibitor. PF-543 assumes multifaceted roles with profound implications. In preclinical investigations, PF-543 has shown promise in suppressing cancer cell proliferation, migration, and invasion. It has also demonstrated potential in reducing inflammation and autoimmune responses. These collective observations suggest that PF-543, through the inhibition of SphK1 and subsequent modulation of S1P levels, may offer therapeutic promise in the treatment of malignancies and the management of immune-related disorders, presenting a compelling proposition for further exploration and translation into clinical applications (Yi et al., 2023). Another important modulator of SphK1 is fingolimod (FTY720, sold under the brand name Gilenya^®^). This prodrug is interesting as it has been approved as the first oral drug to treat multiple sclerosis through interaction with the S1P receptor (Chun et al., 2019). Although it primarily acts as an S1P receptor modulator, it does inhibit SphK1, indirectly affecting SphK/S1P signaling. FTY720 has been extensively used in basic and translational cancer research (White et al., 2016). It is worth noting that FTY720 was also able to inhibit CerS (Lahiri et al., 2009), although at much higher concentrations compared to Fumonisin.

## 3 Sphingolipids at the interface of multiple diseases

Sphingolipids have emerged as key players in various diseases, spanning cancer, neurodegenerative disorders, metabolic diseases, cardiovascular conditions, inflammatory disorders, and infectious diseases (Yu et al., 2012; Trayssac et al., 2018; Wang et al., 2021b; Hernández-Bello et al., 2023). In cancer, dysregulated sphingolipid metabolism contributes to cell proliferation, survival, angiogenesis, and metastasis (Ghandour et al., 2021). Neurodegenerative diseases are characterized by disruptions in sphingolipid homeostasis, impacting neuronal function and promoting inflammation (Chiurchiu et al., 2018; Belarbi et al., 2020). sphingolipid imbalances also play a role in metabolic disorders, impairing insulin signaling and promoting inflammation in conditions like obesity and insulin resistance (Sindhu et al., 2021). Additionally, sphingolipids influence the pathogenesis of cardiovascular diseases, inflammatory disorders, and infectious diseases, affecting immunity, inflammatory responses and disease progression (Lee et al., 2023). Understanding the intricate involvement of sphingolipids in these diseases offers opportunities for targeted therapeutic interventions.

### 3.1 Sphingosine, ceramides, and their phosphorylated derivatives

Sphingosine is a bioactive lipid molecule that plays a critical role in cellular signaling pathways. It is generated from the breakdown of SM, and it can be converted into a number of biologically active derivatives. It can act as a second messenger in various cellular processes, such as apoptosis, differentiation, and proliferation (Bartke and Hannun, 2009). Changes in sphingosine levels have been implicated in the pathogenesis of a number of neurodegenerative disorders, such as Alzheimer’s and Parkinson’s diseases, as well as in cancer, cardiovascular diseases, and inflammatory disorders (Alessenko and Albi, 2020). In terms of its bioactivity, sphingosine has been shown to activate sphingosine-activated protein kinase in cultured rat hippocampal neurons and astrocytes inducing apoptosis (Kanno and Nishizaki, 2011). Additionally, sphingosine inhibited protein kinase C (PKC) in airway smooth muscles resulting in cell death (Tolan et al., 1997). Mitogen-activated protein kinase pathway was inhibited by sphingosine and its methylated derivative dimethylsphingosine promoting cancer cell arrest and apoptosis (Sakakura et al., 1997). Clearly, sphingosine and its derivatives have differential effects on cells (Kim et al., 2008b). Sphingosine has also been shown to regulate the activity of ion channels in the plasma membrane (Rodriguez-Duran et al., 2019), such as the transient receptor potential melastatin channel (Qin et al., 2013). These channels are involved in various cellular processes, such as sensory perception, and sphingosine-mediated regulation of these channels can have important physiological consequences. Additionally, sphingosine has been implicated in the regulation of stress responses in eukaryotic cells. In yeast, for example, sphingosine levels increase in response to heat stress (Dickson, 2008; Cowart et al., 2010).

S1P exhibits greater diversity as a bioactive lipid mediator compared to its precursor, sphingosine, and is involved in regulating various cellular processes associated with both health and disease (Maceyka et al., 2012). S1P is generated through the phosphorylation of sphingosine by SphK1 and SphK2 (Hait and Maiti, 2017). *In vitro* experiments investigating skeletal muscle growth in mice have demonstrated the role of S1P in activating and differentiating muscle satellite cells, as well as facilitating their entry into the cell cycle. Notably, muscular injury induces changes in S1P signaling by upregulating the gene encoding SphK1, suggesting S1P’s involvement in the rapid response to injury (Tan-Chen et al., 2020).

Cer and its phosphorylated derivative, C1P, are prominent signaling molecules in inflammatory responses (Chalfant and Spiegel, 2005). Recently, C1P has been shown to possess an anti-inflammatory property in certain types of cells, although it had previously been found to be pro-inflammation (Gomez-Munoz et al., 2016). C1P is a key regulator of cancer cell migration, and survival and has mitogenic properties (Camacho et al., 2022). Cer and C1P have been extensively studied in terms of their biological functions.

Cer molecules are potent inducers of cell cycle arrest and apoptosis, whereas C1P promotes cell growth and survival (Gomez-Munoz et al., 2016; Hait and Maiti, 2017). Concerning inflammation, Cer and C1P stimulate Ca^+2^-dependent cytosolic phospholipase A2, which subsequently leads to arachidonic acid release and metabolism involving cyclooxygenase 2-mediated inflammation (Hait and Maiti, 2017). C1P enhances arachidonic acid-mediated prostaglandin E_2_ synthesis in wound healing process (Chalfant and Spiegel, 2005; Wijesinghe et al., 2014). Cer can stimulate the proinflammatory transcription factor NF-κB. NF-κB in mammalian cells induces the upregulation of other genes that are involved in inflammatory responses (Gomez-Munoz et al., 2016).

Cer has also been shown to cause a controlled permeabilization of the mitochondrial outer membrane via channel formation (Colombini, 2019). The outer membrane contains some of the enzymes that are responsible for synthesizing Cer (Birbes et al., 2002; Hernandez-Corbacho et al., 2017). However, most of the Cer is generated in the ER at the contact sites between ER and mitochondria, termed mitochondria-associated membranes. Cer generated in the ER can transfer seamlessly from ER to mitochondria without the requirement of assisting proteins (Stiban et al., 2008). Cer channels are dynamic, increasing in size with the addition of more monomers or disassembling with the removal of molecules into the membrane (Siskind et al., 2003). Following an apoptotic signal, the concentration of Cer in the mitochondrial outer membrane increases dramatically leading to the formation of these channels (Siskind, 2005). Targeting SMase to mitochondria and not to other cellular compartments led to Cer accumulation and apoptosis (Birbes et al., 2001). The regulation of the channel size depends on controlling the levels of Cer in the membrane, in addition to the presence of protein and lipid regulators of channel assembly and disassembly (Elrick et al., 2006; Siskind et al., 2006; Stiban et al., 2006; Ganesan and Colombini, 2010; Ganesan et al., 2010; Abou-Ghali and Stiban, 2015; Stiban and Perera, 2015).

### 3.2 Glycosphingolipids

In neurons, GSLs play a crucial role in regulating impulse transmission, as well as the development and differentiation of neuronal cells (Zhang et al., 2019). Additionally, GSLs control other cellular processes, such as apoptosis (Belarbi et al., 2020). As receptors for extracellular signals, GSLs located on the plasma membrane outer surface play a vital role in signal transduction.

Several toxins, including cholera toxin (CTx), heat-labile enterotoxin (LTx), and Shiga toxin (STx), use GSLs as receptors. The B-subunit of these toxins binds to gangliosides GM1 and Gb3 on epithelial membranes in the intestine. This interaction is crucial for providing a toxic effect, leading to immunomodulation in some cells and inducing apoptosis in others (Smith et al., 2004; Lingwood, 2011; Klokk et al., 2016). The interaction between toxins and GSL receptors stimulates signaling pathways that enhance the adaptive immune response, promoting the differentiation of B and T cells to combat the pathogen. Furthermore, CTx’s binding to the GSL at the cell surface of intestinal epithelial cells leads to upregulation of interleukins 6 and 1. This stimulation enhances T-cell co-stimulatory molecule expression and antigen-presenting cells that act as co-stimulatory molecules. Moreover, it may also stimulate T helper cell type 2 in adjuvant responses (Smith et al., 2004).

GSLs have also been implicated in the pathogenesis of several diseases, including neurodegenerative disorders, cancer, and cardiovascular disease (Dang et al., 2018; Belarbi et al., 2020; Ryckman et al., 2020; Zheng et al., 2021). Gangliosides, a type of GSL with sialic acid moieties, are highly abundant in the grey matter of the brain and modulate signaling pathways that alter neuronal activities (Lopez and Schnaar, 2009; Schengrund, 2015). Mutations in genes encoding ganglioside synthases have been shown to increase the risk of neurodegenerative diseases. For example, mutations in the *ST3Gal5* gene that encodes monosialoganglioside GM3 synthase have been linked to infant onset epilepsy, while mutations in the *ST3Gal5* and *B4GalNT1* genes, which encode GM2 synthase, increase the risk of developing multiple sclerosis (Schengrund, 2015).

Failure of GSL homeostasis is associated with Parkinson’s disease (Belarbi et al., 2020), which is characterized by the impairment of the substantia nigra responsible for the generation of dopamine (Beitz, 2014; Belarbi et al., 2020). Parkinson’s disease is associated with the impairment of several cellular processes, including mitochondrial function and protein folding, ER stress, failure in calcium homeostasis, alterations in blood-brain barrier permeability, and neuroinflammation (Belarbi et al., 2020). GSL defects have been linked to the neuroinflammation mechanism in Parkinson’s disease. The presence of mutations in the *GBA* gene, which encodes the enzyme glucocerebrosidase responsible for the degradation of GlcCer into Cer and glucose, is a common risk factor for Parkinson’s disease (Schneider, 2018). The mutation in the *GBA* gene results in the accumulation of GSL disrupting their homeostasis (Schneider, 2018). Another putative mechanism for the relationship between GSLs and Parkinson’s disease is the failure of calcium homeostasis (Schneider, 2018; Belarbi et al., 2020). Studies have shown a direct connection between the accumulation of GlcCer and calcium levels in neurons (Korkotian et al., 1999).

### 3.3 Sphingomyelins

In nervous tissues, SM has many structural and functional roles including myelination, neurogenesis, neuron differentiation, neuronal-glial connection, synaptogenesis, and synaptic transmission (Ong et al., 2015; Signorelli et al., 2021). Myelin sheaths are membranes composed of lipids-rich multilayers, surrounding the axons of the neurons in the peripheral and central nervous system. Myelin sheaths have an essential role in accelerating the impulses along the nerves (Signorelli et al., 2021). During cell development in infants, it has been shown that dietary SM is essential in cognitive development and neurodevelopmental processes such as myelination (Schneider et al., 2019). In the peripheral nervous system, myelin sheaths are formed by Schwann cells, whereas in the central nervous system, oligodendrocytes form these sheets (Simons and Nave, 2015). SM has a functional role in promoting cognitive development modulated by oligodendrocytes (Schneider et al., 2019).

Myelin sheaths are rich in long-chain SM and hydroxy SM (hydroxySM). hydroxySM has a special role in the long-term stabilization of the myelin sheath, and dysregulation of hydroxySMs can lead to early degradation, resulting in neurodegenerative disorders (Signorelli et al., 2021). Additionally, SM is required for the optimal functioning of myelin basic proteins (MBPs) (Signorelli et al., 2021). MBPs are required for the compaction of the multilayers of the myelin sheaths and in their adherence together (Boggs, 2006). SM and MBP interact so that the absence of SM leads to a defect in the incorporation of MBP in the membranes of myelin sheaths (Widder et al., 2020; Signorelli et al., 2021) leading to the partial or full disintegration of the sheath. Because of their function in myelin formation (compaction) and long-term stability, MBPs are regarded as risk factors for Multiple Sclerosis, which is a neurodegenerative disease characterized by myelin sheath degeneration (Boggs, 2006).

Similarly, the accumulation of SM causes different neurological disorders. SM buildup following SMase deficiency is associated with Parkinson’s disease (Pan et al., 2023). Reduced levels of aSMase prevent autophagy, whereas reduced levels of nSMase prevent stem cell differentiation; both mechanisms impair neurogenesis (Signorelli et al., 2021).

## 4 Conclusion

Sphingolipid are lipid molecules whose functions transcend mere membrane composition and structure. They are involved in cellular signaling and physiology. They are intricately made and utilized in several pathways all of which end with the generation of Cer. Cer species in cells are used to mediate myriad cellular functions. In a variety of diseases of the immune and the nervous systems irregularities of sphingolipid metabolism occupy center stage. It is paramount to identify pathways of sphingolipid action in health and in pathophysiology. Sphingolipid functions are multifaceted and very diverse and they affect different cells very differently. Hence, possible treatments for certain immunological and nervous ailments may reside in pinpointing which sphingolipid species is up or downregulated and whether their levels can be pharmacologically or genetically altered in patients. Dissecting sphingolipid metabolism mechanistically in relation to diseases is still in its infancy but offers some promise for future treatments. In the field of sphingolipid biochemistry, current and future trends focus on the identification of specific sphingolipid species that are dysregulated in different diseases. Understanding the intricate mechanisms governing sphingolipid metabolism in the context of diseases presents an exciting avenue for exploration. Pharmacological and genetic interventions aimed at modulating sphingolipid levels could potentially offer novel therapeutic strategies for these ailments. Moreover, studies on the elaborate mechanisms of sphingolipid signaling as bioactive lipids are and will be taking center stage. Investigating the cell-type-specific effects of sphingolipids will be crucial, as these molecules exhibit diverse and multifaceted functions that can vary across different cell types. Sphingolipid research presents an exciting and broad area of investigation for newcomers as well as established researchers.

## References

[B1] Aaltonen M. J. Alecu I. Konig T. Bennett S. A. Shoubridge E. A. (2022). Serine palmitoyltransferase assembles at ER-mitochondria contact sites. Life Sci. Alliance 5, e202101278. 10.26508/lsa.202101278 34785538 PMC8605320

[B2] Abbott S. K. Li H. Munoz S. S. Knoch B. Batterham M. Murphy K. E. (2014). Altered ceramide acyl chain length and ceramide synthase gene expression in Parkinson's disease. Mov. Disord. 29, 518–526. 10.1002/mds.25729 24822250

[B3] Abed Rabbo M. Khodour Y. Kaguni L. S. Stiban J. (2021). Sphingolipid lysosomal storage diseases: from bench to bedside. Lipids Health Dis. 20, 44. 10.1186/s12944-021-01466-0 33941173 PMC8094529

[B4] Abou-Ghali M. Stiban J. (2015). Regulation of ceramide channel formation and disassembly: insights on the initiation of apoptosis. Saudi J. Biol. Sci. 22, 760–772. 10.1016/j.sjbs.2015.03.005 26587005 PMC4625378

[B5] Adachi R. Asano Y. Ogawa K. Oonishi M. Tanaka Y. Kawamoto T. (2018). Pharmacological characterization of synthetic serine palmitoyltransferase inhibitors by biochemical and cellular analyses. Biochem. Biophys. Res. Commun. 497, 1171–1176. 10.1016/j.bbrc.2016.12.182 28042036

[B6] Adamy C. Mulder P. Khouzami L. Andrieu-abadie N. Defer N. Candiani G. (2007). Neutral sphingomyelinase inhibition participates to the benefits of N-acetylcysteine treatment in post-myocardial infarction failing heart rats. J. Mol. Cell Cardiol. 43, 344–353. 10.1016/j.yjmcc.2007.06.010 17707397

[B7] Afrin F. Mateen S. Oman J. Lai J. C. K. Barrott J. J. Pashikanti S. (2023). Natural products and small molecules targeting cellular ceramide metabolism to enhance apoptosis in cancer cells. Cancers (Basel) 15, 4645. 10.3390/cancers15184645 37760612 PMC10527029

[B8] Alessenko A. V. Albi E. (2020). Exploring sphingolipid implications in neurodegeneration. Front. Neurol. 11, 437. 10.3389/fneur.2020.00437 32528400 PMC7254877

[B9] Alexaki A. Clarke B. A. Gavrilova O. Ma Y. Zhu H. Ma X. (2017). *De novo* sphingolipid biosynthesis is required for adipocyte survival and metabolic homeostasis. J. Biol. Chem. 292, 3929–3939. 10.1074/jbc.M116.756460 28100772 PMC5339773

[B10] Alsanafi M. Brown R. D. R. Oh J. Adams D. R. Torta F. Pyne N. J. (2021). Dihydroceramide desaturase functions as an inducer and rectifier of apoptosis: effect of retinol derivatives, antioxidants and phenolic compounds. Cell Biochem. Biophys. 79, 461–475. 10.1007/s12013-021-00990-1 33991313 PMC8551130

[B11] Alyamani M. Kadivar M. Erjefält J. Johansson-Lindbom B. Duan R.-D. Nilsson Å. (2023). Alkaline sphingomyelinase (NPP7) impacts the homeostasis of intestinal T lymphocyte populations. Front. Immunol. 13, 1050625. 10.3389/fimmu.2022.1050625 36741374 PMC9894718

[B12] Amtmann E. Zöller M. Schilling G. (2000). Neutral sphingomyelinase-inhibiting guanidines prevent herpes simplex virus-1 replication. Drugs Exp. Clin. Res. 26 (2), 57–65. 10894556

[B13] Andersson U. Butters T. D. Dwek R. A. Platt F. M. (2000). N-butyldeoxygalactonojirimycin: a more selective inhibitor of glycosphingolipid biosynthesis than N-butyldeoxynojirimycin, *in vitro* and *in vivo* . Biochem. Pharmacol. 59, 821–829. 10.1016/s0006-2952(99)00384-6 10718340

[B14] Andrews N. W. (2019). Solving the secretory acid sphingomyelinase puzzle: insights from lysosome-mediated parasite invasion and plasma membrane repair. Cell Microbiol. 21, e13065. 10.1111/cmi.13065 31155842 PMC6842087

[B15] Anheuser S. Breiden B. Schwarzmann G. Sandhoff K. (2015). Membrane lipids regulate ganglioside GM2 catabolism and GM2 activator protein activity. J. Lipid Res. 56, 1747–1761. 10.1194/jlr.M061036 26175473 PMC4548779

[B16] Arenz C. Giannis A. (2000). Synthesis of the first selective irreversible inhibitor of neutral sphingomyelinase this work was supported by grants from the fonds der Chemischen industrie. C.A. Is grateful to the land of baden-wurttemberg for a scholarship from the landesgraduiertenforderung. Angew. Chem. Int. Ed. Engl. 39, 1440–1442. 10.1002/(sici)1521-3773(20000417)39:8<1440:aid-anie1440>3.0.co;2-r 10777634

[B17] Arenz C. Thutewohl M. Block O. Waldmann H. Altenbach H. J. Giannis A. (2001). Manumycin A and its analogues are irreversible inhibitors of neutral sphingomyelinase. Chembiochem 2, 141–143. 10.1002/1439-7633(20010202)2:2<141:AID-CBIC141>3.0.CO;2-P 11828438

[B18] Aronova S. Wedaman K. Aronov P. A. Fontes K. Ramos K. Hammock B. D. (2008). Regulation of ceramide biosynthesis by TOR complex 2. Cell Metab. 7, 148–158. 10.1016/j.cmet.2007.11.015 18249174 PMC3882310

[B19] Azzam R. Hariri F. El-Hachem N. Kamar A. Dbaibo G. Nemer G. (2013). Regulation of *de novo* ceramide synthesis: the role of dihydroceramide desaturase and transcriptional factors NFATC and Hand2 in the hypoxic mouse heart. DNA Cell Biol. 32, 310–319. 10.1089/dna.2013.1993 23672204 PMC3665309

[B20] Bagdanoff J. T. Donoviel M. S. Nouraldeen A. Carlsen M. Jessop T. C. Tarver J. (2010). Inhibition of sphingosine 1-phosphate lyase for the treatment of rheumatoid arthritis: discovery of (E)-1-(4-((1R,2S,3R)-1,2,3,4-tetrahydroxybutyl)-1H-imidazol-2-yl)ethanone oxime (LX2931) and (1R,2S,3R)-1-(2-(isoxazol-3-yl)-1H-imidazol-4-yl)butane-1,2,3,4-tetraol (LX2932). J. Med. Chem. 53, 8650–8662. 10.1021/jm101183p 21090716

[B21] Baker D. A. Eudaly J. Smith C. D. Obeid L. M. Gilkeson G. S. (2013). Impact of sphingosine kinase 2 deficiency on the development of TNF-alpha-induced inflammatory arthritis. Rheumatol. Int. 33, 2677–2681. 10.1007/s00296-012-2493-2 23011090 PMC3784643

[B22] Bariana T. K. Labarque V. Heremans J. Thys C. De Reys M. Greene D. (2019). Sphingolipid dysregulation due to lack of functional KDSR impairs proplatelet formation causing thrombocytopenia. Haematologica 104, 1036–1045. 10.3324/haematol.2018.204784 30467204 PMC6518879

[B23] Barklis E. Alfadhli A. Kyle J. E. Bramer L. M. Bloodsworth K. J. Barklis R. L. (2021). Ceramide synthase 2 deletion decreases the infectivity of HIV-1. J. Biol. Chem. 296, 100340. 10.1016/j.jbc.2021.100340 33515546 PMC7949126

[B24] Bartke N. Hannun Y. A. (2009). Bioactive sphingolipids: metabolism and function. J. Lipid Res. 50 (Suppl. l), S91–S96. 10.1194/jlr.R800080-JLR200 19017611 PMC2674734

[B25] Bateman K. S. Cherney M. M. Mahuran D. J. Tropak M. James M. N. (2011). Crystal structure of beta-hexosaminidase B in complex with pyrimethamine, a potential pharmacological chaperone. J. Med. Chem. 54, 1421–1429. 10.1021/jm101443u 21265544 PMC3201983

[B26] Baumruker T. Billich A. Brinkmann V. (2007). FTY720, an immunomodulatory sphingolipid mimetic: translation of a novel mechanism into clinical benefit in multiple sclerosis. Expert Opin. Investig. Drugs 16, 283–289. 10.1517/13543784.16.3.283 17302523

[B27] Beitz J. M. (2014). Parkinson's disease: a review. Front. Biosci. Sch. Ed. 6, 65–74. 10.2741/s415 24389262

[B28] Bejaoui K. Wu C. Scheffler M. D. Haan G. Ashby P. Wu L. (2001). SPTLC1 is mutated in hereditary sensory neuropathy, type 1. Nat. Genet. 27, 261–262. 10.1038/85817 11242106

[B29] Belarbi K. Cuvelier E. Bonte M. A. Desplanque M. Gressier B. Devos D. (2020). Glycosphingolipids and neuroinflammation in Parkinson's disease. Mol. Neurodegener. 15, 59. 10.1186/s13024-020-00408-1 33069254 PMC7568394

[B30] Bielawska A. Greenberg M. S. Perry D. Jayadev S. Shayman J. A. McKay C. (1996). (1S,2R)-D-erythro-2-(N-myristoylamino)-1-phenyl-1-propanol as an inhibitor of ceramidase. J. Biol. Chem. 271, 12646–12654. 10.1074/jbc.271.21.12646 8647877

[B31] Bienias K. Fiedorowicz A. Sadowska A. Prokopiuk S. Car H. (2016). Regulation of sphingomyelin metabolism. Pharmacol. Rep. 68, 570–581. 10.1016/j.pharep.2015.12.008 26940196

[B32] Bikman B. T. Guan Y. Shui G. Siddique M. M. Holland W. L. Kim J. Y. (2012). Fenretinide prevents lipid-induced insulin resistance by blocking ceramide biosynthesis. J. Biol. Chem. 287, 17426–17437. 10.1074/jbc.M112.359950 22474281 PMC3366851

[B33] Birbes H. El Bawab S. Hannun Y. A. Obeid L. M. (2001). Selective hydrolysis of a mitochondrial pool of sphingomyelin induces apoptosis. FASEB J. 15, 2669–2679. 10.1096/fj.01-0539com 11726543

[B34] Birbes H. El Bawab S. Obeid L. M. Hannun Y. A. (2002). Mitochondria and ceramide: intertwined roles in regulation of apoptosis. Adv. Enzyme Regul. 42, 113–129. 10.1016/s0065-2571(01)00026-7 12123710

[B35] Bleicher R. J. Cabot M. C. (2002). Glucosylceramide synthase and apoptosis. Biochim. Biophys. Acta 1585, 172–178. 10.1016/s1388-1981(02)00338-4 12531551

[B36] Blitzer J. T. Wang L. Summers S. A. (2020). DES1: a key driver of lipotoxicity in metabolic disease. DNA Cell Biol. 39, 733–737. 10.1089/dna.2020.5402 32181687 PMC7232701

[B37] Boggs J. M. (2006). Myelin basic protein: a multifunctional protein. Cell Mol. Life Sci. 63, 1945–1961. 10.1007/s00018-006-6094-7 16794783 PMC11136439

[B38] Bonzón-Kulichenko E. Schwudke D. Gallardo N. Moltó E. Fernández-Agulló T. Shevchenko A. (2009). Central leptin regulates total ceramide content and sterol regulatory element binding protein-1C proteolytic maturation in rat white adipose tissue. Endocrinology 150, 169–178. 10.1210/en.2008-0505 18801905

[B39] Boyd R. A. Majumder S. Stiban J. Mavodza G. Straus A. J. Kempelingaiah S. K. (2023). The heat shock protein Hsp27 controls mitochondrial function by modulating ceramide generation. Cell Rep. 42, 113081. 10.1016/j.celrep.2023.113081 37689067 PMC10591768

[B40] Boyden L. M. Vincent N. G. Zhou J. Hu R. Craiglow B. G. Bayliss S. J. (2017). Mutations in KDSR cause recessive progressive symmetric erythrokeratoderma. Am. J. Hum. Genet. 100, 978–984. 10.1016/j.ajhg.2017.05.003 28575652 PMC5473720

[B41] Brachtendorf S. El-Hindi K. Grösch S. (2019). Ceramide synthases in cancer therapy and chemoresistance. Prog. Lipid Res. 74, 160–185. 10.1016/j.plipres.2019.04.002 30953657

[B42] Breiden B. Sandhoff K. (2021). Acid sphingomyelinase, a lysosomal and secretory phospholipase C, is key for cellular phospholipid catabolism. Int. J. Mol. Sci. 22, 9001. 10.3390/ijms22169001 34445706 PMC8396676

[B43] Breimer M. E. Saljo K. Barone A. Teneberg S. (2017). Glycosphingolipids of human embryonic stem cells. Glycoconj J. 34, 713–723. 10.1007/s10719-016-9706-y 27325407 PMC5711972

[B44] Breslow D. K. Collins S. R. Bodenmiller B. Aebersold R. Simons K. Shevchenko A. (2010). Orm family proteins mediate sphingolipid homeostasis. Nature 463, 1048–1053. 10.1038/nature08787 20182505 PMC2877384

[B45] Breslow D. K. Weissman J. S. (2010). Membranes in balance: mechanisms of sphingolipid homeostasis. Mol. Cell 40, 267–279. 10.1016/j.molcel.2010.10.005 20965421 PMC2987644

[B46] Bu Y. Wu H. Deng R. Wang Y. (2021). Therapeutic potential of SphK1 inhibitors based on abnormal expression of SphK1 in inflammatory immune related-diseases. Front. Pharmacol. 12, 733387. 10.3389/fphar.2021.733387 34737701 PMC8560647

[B47] Buehrer B. M. Bell R. M. (1992). Inhibition of sphingosine kinase *in vitro* and in platelets. Implications for signal transduction pathways. J. Biol. Chem. 267, 3154–3159. 10.1016/s0021-9258(19)50708-6 1310683

[B48] Cai J. Liu Y. Li Q. Wen Z. Li Y. Chen X. (2022). Ceramide synthase 3 affects invasion and metastasis of hepatocellular carcinoma via the SMAD6 gene. Zhong Nan Da Xue Xue Bao Yi Xue Ban. 47, 588–599. 10.11817/j.issn.1672-7347.2022.210477 35753729 PMC10929919

[B49] Camacho L. Ouro A. Gomez-Larrauri A. Carracedo A. Gomez-Munoz A. (2022). Implication of ceramide kinase/C1P in cancer development and progression. Cancers (Basel) 14, 227. 10.3390/cancers14010227 35008391 PMC8750078

[B50] Camacho L. Simbari F. Garrido M. Abad J. L. Casas J. Delgado A. (2012). 3-Deoxy-3,4-dehydro analogs of XM462. Preparation and activity on sphingolipid metabolism and cell fate. Bioorg Med. Chem. 20, 3173–3179. 10.1016/j.bmc.2012.03.073 22537678

[B51] Cantalupo A. Sasset L. Gargiulo A. Rubinelli L. Del Gaudio I. Benvenuto D. (2020). Endothelial sphingolipid *de novo* synthesis controls blood pressure by regulating signal transduction and NO via ceramide. Hypertension 75, 1279–1288. 10.1161/HYPERTENSIONAHA.119.14507 32172624 PMC7145736

[B52] Casasampere M. Ordonez Y. F. Pou A. Casas J. (2016). Inhibitors of dihydroceramide desaturase 1: therapeutic agents and pharmacological tools to decipher the role of dihydroceramides in cell biology. Chem. Phys. Lipids 197, 33–44. 10.1016/j.chemphyslip.2015.07.025 26248324

[B53] Chalfant C. E. Spiegel S. (2005). Sphingosine 1-phosphate and ceramide 1-phosphate: expanding roles in cell signaling. J. Cell Sci. 118, 4605–4612. 10.1242/jcs.02637 16219683

[B54] Chatterjee S. (1999). Neutral sphingomyelinase: past, present and future. Chem. Phys. Lipids 102, 79–96. 10.1016/s0009-3084(99)00077-8 11001563

[B55] Chaurasia B. Kaddai V. A. Lancaster G. I. Henstridge D. C. Sriram S. Galam D. L. (2016). Adipocyte ceramides regulate subcutaneous adipose browning, inflammation, and metabolism. Cell Metab. 24, 820–834. 10.1016/j.cmet.2016.10.002 27818258

[B56] Chen J. Wei Z. Wang Y. Long M. Wu W. Kuca K. (2021). Fumonisin B1: mechanisms of toxicity and biological detoxification progress in animals. Food Chem. Toxicol. 149, 111977. 10.1016/j.fct.2021.111977 33428988

[B57] Chen Y. Zhang P. Xu S. C. Yang L. Voss U. Ekblad E. (2015). Enhanced colonic tumorigenesis in alkaline sphingomyelinase (NPP7) knockout mice. Mol. Cancer Ther. 14, 259–267. 10.1158/1535-7163.MCT-14-0468-T 25381265

[B58] Cheng Y. Wu J. Hertervig E. Lindgren S. Duan D. Nilsson A. (2007). Identification of aberrant forms of alkaline sphingomyelinase (NPP7) associated with human liver tumorigenesis. Br. J. Cancer 97, 1441–1448. 10.1038/sj.bjc.6604013 17923876 PMC2360232

[B59] Chiba Y. Takeuchi H. Sakai H. Misawa M. (2010). SKI-II, an inhibitor of sphingosine kinase, ameliorates antigen-induced bronchial smooth muscle hyperresponsiveness, but not airway inflammation, in mice. J. Pharmacol. Sci. 114, 304–310. 10.1254/jphs.10202fp 20948165

[B60] Chiurchiu V. Leuti A. Maccarrone M. (2018). Bioactive lipids and chronic inflammation: managing the fire within. Front. Immunol. 9, 38. 10.3389/fimmu.2018.00038 29434586 PMC5797284

[B61] Chun J. Kihara Y. Jonnalagadda D. Blaho V. A. (2019). Fingolimod: lessons learned and new opportunities for treating multiple sclerosis and other disorders. Annu. Rev. Pharmacol. Toxicol. 59, 149–170. 10.1146/annurev-pharmtox-010818-021358 30625282 PMC6392001

[B62] Chung N. Mao C. Heitman J. Hannun Y. A. Obeid L. M. (2001). Phytosphingosine as a specific inhibitor of growth and nutrient import in *Saccharomyces cerevisiae* . J. Biol. Chem. 276, 35614–35621. 10.1074/jbc.M105653200 11468289

[B63] Cingolani F. Casasampere M. Sanllehi P. Casas J. Bujons J. Fabrias G. (2014). Inhibition of dihydroceramide desaturase activity by the sphingosine kinase inhibitor SKI II. J. Lipid Res. 55, 1711–1720. 10.1194/jlr.M049759 24875537 PMC4109765

[B64] Clarke B. A. Majumder S. Zhu H. Lee Y. T. Kono M. Li C. (2019). The Ormdl genes regulate the sphingolipid synthesis pathway to ensure proper myelination and neurologic function in mice. Elife 8, e51067. 10.7554/eLife.51067 31880535 PMC6934382

[B65] Clarke C. J. Hannun Y. A. (2006). Neutral sphingomyelinases and nSMase2: bridging the gaps. Biochim. Biophys. Acta 1758, 1893–1901. 10.1016/j.bbamem.2006.06.025 16938269

[B66] Coant N. Garcia-Barros M. Zhang Q. Obeid L. M. Hannun Y. A. (2018). AKT as a key target for growth promoting functions of neutral ceramidase in colon cancer cells. Oncogene 37, 3852–3863. 10.1038/s41388-018-0236-x 29662189 PMC6041258

[B67] Coant N. Sakamoto W. Mao C. Hannun Y. A. (2017). Ceramidases, roles in sphingolipid metabolism and in health and disease. Adv. Biol. Regul. 63, 122–131. 10.1016/j.jbior.2016.10.002 27771292 PMC5330250

[B68] Colombini M. (2019). Ceramide channels. Adv. Exp. Med. Biol. 1159, 33–48. 10.1007/978-3-030-21162-2_3 31502198

[B69] Corcoran C. A. He Q. Ponnusamy S. Ogretmen B. Huang Y. Sheikh M. S. (2008). Neutral sphingomyelinase-3 is a DNA damage and nongenotoxic stress-regulated gene that is deregulated in human malignancies. Mol. Cancer Res. 6, 795–807. 10.1158/1541-7786.MCR-07-2097 18505924 PMC2642592

[B70] Costa-Pinheiro P. Heher A. Raymond M. H. Jividen K. Shaw J. J. Paschal B. M. (2020). Role of SPTSSB-regulated *de novo* sphingolipid synthesis in prostate cancer depends on androgen receptor signaling. iScience 23, 101855. 10.1016/j.isci.2020.101855 33313495 PMC7721643

[B71] Cowart L. A. Gandy J. L. Tholanikunnel B. Hannun Y. A. (2010). Sphingolipids mediate formation of mRNA processing bodies during the heat-stress response of *Saccharomyces cerevisiae* . Biochem. J. 431, 31–38. 10.1042/BJ20100307 20629639 PMC3804835

[B72] Dagan A. Wang C. Fibach E. Gatt S. (2003). Synthetic, non-natural sphingolipid analogs inhibit the biosynthesis of cellular sphingolipids, elevate ceramide and induce apoptotic cell death. Biochim. Biophys. Acta 1633, 161–169. 10.1016/s1388-1981(03)00122-7 14499735

[B73] Dang V. T. Zhong L. H. Huang A. Deng A. Werstuck G. H. (2018). Glycosphingolipids promote pro-atherogenic pathways in the pathogenesis of hyperglycemia-induced accelerated atherosclerosis. Metabolomics 14, 92. 10.1007/s11306-018-1392-2 30830446

[B74] DAngelo G. Moorthi S. Luberto C. (2018). Role and function of sphingomyelin biosynthesis in the development of cancer. Adv. Cancer Res. 140, 61–96. 10.1016/bs.acr.2018.04.009 30060817

[B75] Davis D. Kannan M. Wattenberg B. (2018). Orm/ORMDL proteins: gate guardians and master regulators. Adv. Biol. Regul. 70, 3–18. 10.1016/j.jbior.2018.08.002 30193828 PMC6251742

[B76] Davis D. L. Gable K. Suemitsu J. Dunn T. M. Wattenberg B. W. (2019). The ORMDL/Orm-serine palmitoyltransferase (SPT) complex is directly regulated by ceramide: reconstitution of SPT regulation in isolated membranes. J. Biol. Chem. 294, 5146–5156. 10.1074/jbc.RA118.007291 30700557 PMC6442065

[B77] Deng P. Hoffman J. B. Petriello M. C. Wang C. Y. Li X. S. Kraemer M. P. (2020). Dietary inulin decreases circulating ceramides by suppressing neutral sphingomyelinase expression and activity in mice. J. Lipid Res. 61, 45–53. 10.1194/jlr.RA119000346 31604806 PMC6939596

[B78] Dersh D. Iwamoto Y. Argon Y. (2016). Tay-Sachs disease mutations in HEXA target the alpha chain of hexosaminidase A to endoplasmic reticulum-associated degradation. Mol. Biol. Cell 27, 3813–3827. 10.1091/mbc.E16-01-0012 27682588 PMC5170605

[B79] Desnick R. J. Schuchman E. H. Astrin K. H. Cheng S. H. (2013). “Chapter 28 - therapies for lysosomal storage diseases,” in Emery and rimoin's principles and practice of medical genetics. Editors Rimoin D. Pyeritz R. Korf B. Sixth Edition (Oxford: Academic Press), 1–30.

[B80] Dickson R. C. (2008). Thematic review series: sphingolipids. New insights into sphingolipid metabolism and function in budding yeast. J. Lipid Res. 49, 909–921. 10.1194/jlr.R800003-JLR200 18296751 PMC2311445

[B81] DMello N P. Childress A. M. Franklin D. S. Kale S. P. Pinswasdi C. Jazwinski S. M. (1994). Cloning and characterization of LAG1, a longevity-assurance gene in yeast. J. Biol. Chem. 269, 15451–15459. 10.1016/s0021-9258(17)40700-9 8195187

[B82] Dogra N. Warburton C. McMaster W. R. (2007). Leishmania major abrogates gamma interferon-induced gene expression in human macrophages from a global perspective. Infect. Immun. 75, 3506–3515. 10.1128/IAI.00277-07 17470541 PMC1932916

[B83] Dolgin V. Straussberg R. Xu R. Mileva I. Yogev Y. Khoury R. (2019). DEGS1 variant causes neurological disorder. Eur. J. Hum. Genet. 27, 1668–1676. 10.1038/s41431-019-0444-z 31186544 PMC6871177

[B84] Draper J. M. Xia Z. Smith R. A. Zhuang Y. Wang W. Smith C. D. (2011). Discovery and evaluation of inhibitors of human ceramidase. Mol. Cancer Ther. 10, 2052–2061. 10.1158/1535-7163.MCT-11-0365 21885864 PMC3213284

[B85] Duan R. D. (2006). Alkaline sphingomyelinase: an old enzyme with novel implications. Biochim. Biophys. Acta 1761, 281–291. 10.1016/j.bbalip.2006.03.007 16631405

[B86] Duan R. D. (2018). Alkaline sphingomyelinase (NPP7) in hepatobiliary diseases: a field that needs to be closely studied. World J. Hepatol. 10, 246–253. 10.4254/wjh.v10.i2.246 29527260 PMC5838443

[B87] Dybkaer K. Iqbal J. Zhou G. Geng H. Xiao L. Schmitz A. (2007). Genome wide transcriptional analysis of resting and IL2 activated human natural killer cells: gene expression signatures indicative of novel molecular signaling pathways. BMC Genomics 8, 230. 10.1186/1471-2164-8-230 17623099 PMC1959522

[B88] Ebel P. Vom Dorp K. Petrasch-Parwez E. Zlomuzica A. Kinugawa K. Mariani J. (2013). Inactivation of ceramide synthase 6 in mice results in an altered sphingolipid metabolism and behavioral abnormalities. J. Biol. Chem. 288, 21433–21447. 10.1074/jbc.M113.479907 23760501 PMC3774410

[B89] Ehlert K. Frosch M. Fehse N. Zander A. Roth J. Vormoor J. (2007). Farber disease: clinical presentation, pathogenesis and a new approach to treatment. Pediatr. Rheumatol. Online J. 5, 15. 10.1186/1546-0096-5-15 17603888 PMC1920510

[B90] Ekici B. Kurkcu D. Caliskan M. (2012). Farber disease: a clinical diagnosis. J. Pediatr. Neurosci. 7, 154–155. 10.4103/1817-1745.102592 23248705 PMC3519083

[B91] El-Hindi K. Brachtendorf S. Hartel J. C. Oertel S. Birod K. Merz N. (2022). T-Cell-Specific CerS4 depletion prolonged inflammation and enhanced tumor burden in the AOM/DSS-Induced CAC model. Int. J. Mol. Sci. 23, 1866. 10.3390/ijms23031866 35163788 PMC8837088

[B92] El Malki K. Wehling P. Alt F. Sandhoff R. Zahnreich S. Ustjanzew A. (2023). Glucosylceramide synthase inhibitors induce ceramide accumulation and sensitize H3K27 mutant diffuse midline glioma to irradiation. Int. J. Mol. Sci. 24, 9905. 10.3390/ijms24129905 37373053 PMC10298524

[B93] Elrick M. J. Fluss S. Colombini M. (2006). Sphingosine, a product of ceramide hydrolysis, influences the formation of ceramide channels. Biophys. J. 91, 1749–1756. 10.1529/biophysj.106.088443 16782799 PMC1544278

[B94] Endo K. Akiyama T. Kobayashi S. Okada M. (1996). Degenerative spermatocyte, a novel gene encoding a transmembrane protein required for the initiation of meiosis in Drosophila spermatogenesis. Mol. Gen. Genet. 253, 157–165. 10.1007/s004380050308 9003299

[B95] Ernst D. Murphy S. M. Sathiyanadan K. Wei Y. Othman A. Laura M. (2015). Novel HSAN1 mutation in serine palmitoyltransferase resides at a putative phosphorylation site that is involved in regulating substrate specificity. Neuromolecular Med. 17, 47–57. 10.1007/s12017-014-8339-1 25567748 PMC4326654

[B96] Espaillat M. P. Snider A. J. Qiu Z. Channer B. Coant N. Schuchman E. H. (2018). Loss of acid ceramidase in myeloid cells suppresses intestinal neutrophil recruitment. FASEB J. 32, 2339–2353. 10.1096/fj.201700585R 29259036 PMC6207279

[B97] Fabrias G. Munoz-Olaya J. Cingolani F. Signorelli P. Casas J. Gagliostro V. (2012). Dihydroceramide desaturase and dihydrosphingolipids: debutant players in the sphingolipid arena. Prog. Lipid Res. 51, 82–94. 10.1016/j.plipres.2011.12.002 22200621

[B98] Ficicioglu C. (2008). Review of miglustat for clinical management in Gaucher disease type 1. Ther. Clin. Risk Manag. 4, 425–431. 10.2147/tcrm.s6865 18728838 PMC2504062

[B99] French K. J. Upson J. J. Keller S. N. Zhuang Y. Yun J. K. Smith C. D. (2006). Antitumor activity of sphingosine kinase inhibitors. J. Pharmacol. Exp. Ther. 318, 596–603. 10.1124/jpet.106.101345 16632640

[B100] French K. J. Zhuang Y. Maines L. W. Gao P. Wang W. Beljanski V. (2010). Pharmacology and antitumor activity of ABC294640, a selective inhibitor of sphingosine kinase-2. J. Pharmacol. Exp. Ther. 333, 129–139. 10.1124/jpet.109.163444 20061445 PMC2846016

[B101] Fresques T. Niles B. Aronova S. Mogri H. Rakhshandehroo T. Powers T. (2015). Regulation of ceramide synthase by casein kinase 2-dependent phosphorylation in *Saccharomyces cerevisiae* . J. Biol. Chem. 290, 1395–1403. 10.1074/jbc.M114.621086 25429105 PMC4340386

[B102] Fujii T. Tanaka Y. Oki H. Sato S. Shibata S. Maru T. (2021). A new brain-penetrant glucosylceramide synthase inhibitor as potential Therapeutics for Gaucher disease. J. Neurochem. 159, 543–553. 10.1111/jnc.15492 34398463 PMC9293090

[B103] Futerman A. H. Hannun Y. A. (2004). The complex life of simple sphingolipids. EMBO Rep. 5, 777–782. 10.1038/sj.embor.7400208 15289826 PMC1299119

[B104] Futerman A. H. Platt F. M. (2017). The metabolism of glucocerebrosides - from 1965 to the present. Mol. Genet. Metab. 120, 22–26. 10.1016/j.ymgme.2016.11.390 27955980

[B105] Ganesan V. Colombini M. (2010). Regulation of ceramide channels by Bcl-2 family proteins. FEBS Lett. 584, 2128–2134. 10.1016/j.febslet.2010.02.032 20159016

[B106] Ganesan V. Perera M. N. Colombini D. Datskovskiy D. Chadha K. Colombini M. (2010). Ceramide and activated Bax act synergistically to permeabilize the mitochondrial outer membrane. Apoptosis 15, 553–562. 10.1007/s10495-009-0449-0 20101465

[B107] Gao K. F. Zhao Y. F. Liao W. J. Xu G. L. Zhang J. D. (2022). CERS6-AS1 promotes cell proliferation and represses cell apoptosis in pancreatic cancer via miR-195-5p/WIPI2 axis. Kaohsiung J. Med. Sci. 38, 542–553. 10.1002/kjm2.12522 35199935 PMC11896295

[B108] Gao P. Peterson Y. K. Smith R. A. Smith C. D. (2012). Characterization of isoenzyme-selective inhibitors of human sphingosine kinases. PLoS One 7, e44543. 10.1371/journal.pone.0044543 22970244 PMC3438171

[B109] Garbade S. F. Zielonka M. Mechler K. Kölker S. Hoffmann G. F. Staufner C. (2020). FDA orphan drug designations for lysosomal storage disorders - a cross-sectional analysis. PLoS One 15, e0230898. 10.1371/journal.pone.0230898 32267884 PMC7141691

[B110] Garcia-Barros M. Coant N. Kawamori T. Wada M. Snider A. J. Truman J. P. (2016). Role of neutral ceramidase in colon cancer. FASEB J. 30, 4159–4171. 10.1096/fj.201600611R 27609772 PMC5102116

[B111] Gault C. R. Obeid L. M. Hannun Y. A. (2010). An overview of sphingolipid metabolism: from synthesis to breakdown. Adv. Exp. Med. Biol. 688, 1–23. 10.1007/978-1-4419-6741-1_1 20919643 PMC3069696

[B112] Gebai A. Gorelik A. Li Z. Illes K. Nagar B. (2018). Structural basis for the activation of acid ceramidase. Nat. Commun. 9, 1621. 10.1038/s41467-018-03844-2 29692406 PMC5915598

[B113] Geeraert L. Mannaerts G. P. van Veldhoven P. P. (1997). Conversion of dihydroceramide into ceramide: involvement of a desaturase. Biochem. J. 327 (Pt 1), 125–132. 10.1042/bj3270125 9355743 PMC1218771

[B114] Ghandour B. Dbaibo G. Darwiche N. (2021). The unfolding role of ceramide in coordinating retinoid-based cancer therapy. Biochem. J. 478, 3621–3642. 10.1042/BCJ20210368 34648006

[B115] Ginkel C. Hartmann D. vom Dorp K. Zlomuzica A. Farwanah H. Eckhardt M. (2012). Ablation of neuronal ceramide synthase 1 in mice decreases ganglioside levels and expression of myelin-associated glycoprotein in oligodendrocytes. J. Biol. Chem. 287, 41888–41902. 10.1074/jbc.M112.413500 23074226 PMC3516736

[B116] Gomez-Munoz A. Kong J. Salh B. Steinbrecher U. P. (2003). Sphingosine-1-phosphate inhibits acid sphingomyelinase and blocks apoptosis in macrophages. FEBS Lett. 539, 56–60. 10.1016/s0014-5793(03)00197-2 12650926

[B117] Gomez-Munoz A. Kong J. Y. Salh B. Steinbrecher U. P. (2004). Ceramide-1-phosphate blocks apoptosis through inhibition of acid sphingomyelinase in macrophages. J. Lipid Res. 45, 99–105. 10.1194/jlr.M300158-JLR200 14523050

[B118] Gomez-Munoz A. Presa N. Gomez-Larrauri A. Rivera I. G. Trueba M. Ordonez M. (2016). Control of inflammatory responses by ceramide, sphingosine 1-phosphate and ceramide 1-phosphate. Prog. Lipid Res. 61, 51–62. 10.1016/j.plipres.2015.09.002 26703189

[B119] Gorelik A. Liu F. Illes K. Nagar B. (2017). Crystal structure of the human alkaline sphingomyelinase provides insights into substrate recognition. J. Biol. Chem. 292, 7087–7094. 10.1074/jbc.M116.769273 28292932 PMC5409475

[B120] Green C. D. Weigel C. Oyeniran C. James B. N. Davis D. Mahawar U. (2021). CRISPR/Cas9 deletion of ORMDLs reveals complexity in sphingolipid metabolism. J. Lipid Res. 62, 100082. 10.1016/j.jlr.2021.100082 33939982 PMC8167824

[B121] Gulbins E. Petrache I. (2013). Sphingolipids: basic science and drug development. Vienna: Springer.

[B122] Guo Q. Zhang T. Meng N. Duan Y. Meng Y. Sun D. (2020). Sphingolipids are required for exocyst polarity and exocytic secretion in *Saccharomyces cerevisiae* . Cell Biosci. 10, 53. 10.1186/s13578-020-00406-2 32257111 PMC7106735

[B123] Gupta S. D. Gable K. Han G. Borovitskaya A. Selby L. Dunn T. M. (2009). Tsc10p and FVT1: topologically distinct short-chain reductases required for long-chain base synthesis in yeast and mammals. J. Lipid Res. 50, 1630–1640. 10.1194/jlr.M800580-JLR200 19141869 PMC2724050

[B124] Hait N. C. Maiti A. (2017). The role of sphingosine-1-phosphate and ceramide-1-phosphate in inflammation and cancer. Mediat. Inflamm. 2017, 4806541. 10.1155/2017/4806541 PMC5705877 29269995

[B125] Hakomori S. (2003). Structure, organization, and function of glycosphingolipids in membrane. Curr. Opin. Hematol. 10, 16–24. 10.1097/00062752-200301000-00004 12483107

[B126] Hammad S. M. Hunt K. J. Baker N. L. Klein R. L. Lopes-Virella M. F. (2022). Diabetes and kidney dysfunction markedly alter the content of sphingolipids carried by circulating lipoproteins. J. Clin. Lipidol. 16, 173–183. 10.1016/j.jacl.2021.12.004 35148982 PMC12882810

[B127] Hammad S. M. Lopes-Virella M. F. (2023). Circulating sphingolipids in insulin resistance, diabetes and associated complications. Int. J. Mol. Sci. 24, 14015. 10.3390/ijms241814015 37762318 PMC10531201

[B128] Han G. Gupta S. D. Gable K. Niranjanakumari S. Moitra P. Eichler F. (2009). Identification of small subunits of mammalian serine palmitoyltransferase that confer distinct acyl-CoA substrate specificities. Proc. Natl. Acad. Sci. U. S. A. 106, 8186–8191. 10.1073/pnas.0811269106 19416851 PMC2688822

[B129] Hanada K. (2003). Serine palmitoyltransferase, a key enzyme of sphingolipid metabolism. Biochim. Biophys. Acta 1632, 16–30. 10.1016/s1388-1981(03)00059-3 12782147

[B130] Hanada K. Nishijima M. Fujita T. Kobayashi S. (2000). Specificity of inhibitors of serine palmitoyltransferase (SPT), a key enzyme in sphingolipid biosynthesis, in intact cells. A novel evaluation system using an SPT-defective mammalian cell mutant. Biochem. Pharmacol. 59, 1211–1216. 10.1016/s0006-2952(00)00251-3 10736421

[B131] Hannun Y. A. Bell R. M. (1989). Functions of sphingolipids and sphingolipid breakdown products in cellular regulation. Science 243, 500–507. 10.1126/science.2643164 2643164

[B132] Harel R. Futerman A. H. (1993). Inhibition of sphingolipid synthesis affects axonal outgrowth in cultured hippocampal neurons. J. Biol. Chem. 268, 14476–14481. 10.1016/s0021-9258(19)85263-8 8314804

[B133] Harrison P. J. Dunn T. M. Campopiano D. J. (2018). Sphingolipid biosynthesis in man and microbes. Nat. Prod. Rep. 35, 921–954. 10.1039/c8np00019k 29863195 PMC6148460

[B134] He X. Dagan A. Gatt S. Schuchman E. H. (2005). Simultaneous quantitative analysis of ceramide and sphingosine in mouse blood by naphthalene-2,3-dicarboxyaldehyde derivatization after hydrolysis with ceramidase. Anal. Biochem. 340, 113–122. 10.1016/j.ab.2005.01.058 15802137

[B135] Henry B. Ziobro R. Becker K. A. Kolesnick R. Gulbins E. (2013). Acid sphingomyelinase. Handb. Exp. Pharmacol., 77–88. 10.1007/978-3-7091-1368-4_4 23579450

[B136] Hernández-Bello F. Franco M. Pérez-Méndez Ó. Donis-Maturano L. Zarco-Olvera G. Bautista-Pérez R. (2023). Sphingolipid metabolism and its relationship with cardiovascular, renal and metabolic diseases. Arch. Cardiol. Mex. 93, 88–95. 10.24875/ACM.21000333 36757794 PMC10161840

[B137] Hernandez-Corbacho M. J. Salama M. F. Canals D. Senkal C. E. Obeid L. M. (2017). Sphingolipids in mitochondria. Biochim. Biophys. Acta Mol. Cell Biol. Lipids 1862, 56–68. 10.1016/j.bbalip.2016.09.019 27697478 PMC5125891

[B138] Hernández-Tiedra S. Fabriàs G. Dávila D. Salanueva Í J. Casas J. Montes L. R. (2016). Dihydroceramide accumulation mediates cytotoxic autophagy of cancer cells via autolysosome destabilization. Autophagy 12, 2213–2229. 10.1080/15548627.2016.1213927 27635674 PMC5103338

[B139] Heung L. J. Luberto C. Del Poeta M. (2006). Role of sphingolipids in microbial pathogenesis. Infect. Immun. 74, 28–39. 10.1128/IAI.74.1.28-39.2006 16368954 PMC1346627

[B140] Holland W. L. Brozinick J. T. Wang L. P. Hawkins E. D. Sargent K. M. Liu Y. (2007). Inhibition of ceramide synthesis ameliorates glucocorticoid-saturated-fat-and obesity-induced insulin resistance. Cell Metab. 5, 167–179. 10.1016/j.cmet.2007.01.002 17339025

[B141] Hornemann T. Richard S. Rutti M. F. Wei Y. von Eckardstein A. (2006). Cloning and initial characterization of a new subunit for mammalian serine-palmitoyltransferase. J. Biol. Chem. 281, 37275–37281. 10.1074/jbc.M608066200 17023427

[B142] Hultberg B. Isaksson A. Lindgren A. Israelsson B. Brattstrom L. (1996). beta-Hexosaminidase isoenzymes A and B in middle-aged and elderly subjects: determinants of plasma levels and relation to vascular disease. Ann. Clin. Biochem. 33 (Pt 5), 432–437. 10.1177/000456329603300506 8888976

[B143] Idkowiak-Baldys J. Apraiz A. Li L. Rahmaniyan M. Clarke C. J. Kraveka J. M. (2010). Dihydroceramide desaturase activity is modulated by oxidative stress. Biochem. J. 427, 265–274. 10.1042/BJ20091589 20105137 PMC3086801

[B144] Ikushiro H. Hayashi H. Kagamiyama H. (2003). Bacterial serine palmitoyltransferase: a water-soluble homodimeric prototype of the eukaryotic enzyme. Biochim. Biophys. Acta 1647, 116–120. 10.1016/s1570-9639(03)00074-8 12686119

[B145] Ikushiro H. Hayashi H. Kagamiyama H. (2004). Reactions of serine palmitoyltransferase with serine and molecular mechanisms of the actions of serine derivatives as inhibitors. Biochemistry 43, 1082–1092. 10.1021/bi035706v 14744154

[B146] Inokuchi J. Radin N. S. (1987). Preparation of the active isomer of 1-phenyl-2-decanoylamino-3-morpholino-1-propanol, inhibitor of murine glucocerebroside synthetase. J. Lipid Res. 28, 565–571. 10.1016/s0022-2275(20)38673-9 2955067

[B147] Insausti-Urkia N. Solsona-Vilarrasa E. Garcia-Ruiz C. Fernandez-Checa J. C. (2020). Sphingomyelinases and liver diseases. Biomolecules 10, 1497. 10.3390/biom10111497 33143193 PMC7692672

[B148] Ishino Y. Komatsu N. Sakata K. T. Yoshikawa D. Tani M. Maeda T. (2022). Regulation of sphingolipid biosynthesis in the endoplasmic reticulum via signals from the plasma membrane in budding yeast. Febs J. 289, 457–472. 10.1111/febs.16189 34492164

[B149] Issleny B. M. Jamjoum R. Majumder S. Stiban J. (2023). “Sphingolipids: from structural components to signaling hubs,” in The enzymes (United States: Academic Press). 10.1016/bs.enz.2023.07.003 37945171

[B150] Jaffrezou J. P. Herbert J. M. Levade T. Gau M. N. Chatelain P. Laurent G. (1991). Reversal of multidrug resistance by calcium channel blocker SR33557 without photoaffinity labeling of P-glycoprotein. J. Biol. Chem. 266, 19858–19864. 10.1016/s0021-9258(18)55070-5 1918089

[B151] Jagle S. Hsu H. H. Juratli H. A. Zimmer A. D. Prieschl A. Alter S. (2023). Pathogenic variants in the SPTLC1 gene cause hyperkeratosis lenticularis perstans. Br. J. Dermatol 188, 94–99. 10.1093/bjd/ljac019 36689507

[B152] Jenkins R. W. Canals D. Hannun Y. A. (2009). Roles and regulation of secretory and lysosomal acid sphingomyelinase. Cell Signal 21, 836–846. 10.1016/j.cellsig.2009.01.026 19385042 PMC3488588

[B153] Jin J. Hou Q. Mullen T. D. Zeidan Y. H. Bielawski J. Kraveka J. M. (2008). Ceramide generated by sphingomyelin hydrolysis and the salvage pathway is involved in hypoxia/reoxygenation-induced Bax redistribution to mitochondria in NT-2 cells. J. Biol. Chem. 283, 26509–26517. 10.1074/jbc.M801597200 18676372 PMC2546549

[B154] Jin J. Mullen T. D. Hou Q. Bielawski J. Bielawska A. Zhang X. (2009). AMPK inhibitor Compound C stimulates ceramide production and promotes Bax redistribution and apoptosis in MCF7 breast carcinoma cells. J. Lipid Res. 50, 2389–2397. 10.1194/jlr.M900119-JLR200 19528633 PMC2781311

[B155] Johnson E. L. Heaver S. L. Waters J. L. Kim B. I. Bretin A. Goodman A. L. (2020). Sphingolipids produced by gut bacteria enter host metabolic pathways impacting ceramide levels. Nat. Commun. 11, 2471. 10.1038/s41467-020-16274-w 32424203 PMC7235224

[B156] Johnson V. J. He Q. Osuchowski M. F. Sharma R. P. (2004). Disruption of sphingolipid homeostasis by myriocin, a mycotoxin, reduces thymic and splenic T-lymphocyte populations. Toxicology 201, 67–75. 10.1016/j.tox.2004.04.019 15297021

[B157] Kahan B. D. (2004). FTY720: from bench to bedside. Transpl. Proc. 36, 531S–543s. 10.1016/j.transproceed.2004.01.092 15041402

[B158] Kanno T. Nishizaki T. (2011). Sphingosine induces apoptosis in hippocampal neurons and astrocytes by activating caspase-3/-9 via a mitochondrial pathway linked to SDK/14-3-3 protein/Bax/cytochrome c. J. Cell Physiol. 226, 2329–2337. 10.1002/jcp.22571 21660956

[B159] Kapitonov D. Allegood J. C. Mitchell C. Hait N. C. Almenara J. A. Adams J. K. (2009). Targeting sphingosine kinase 1 inhibits Akt signaling, induces apoptosis, and suppresses growth of human glioblastoma cells and xenografts. Cancer Res. 69, 6915–6923. 10.1158/0008-5472.CAN-09-0664 19723667 PMC2752891

[B160] Katsume A. Tokunaga Y. Hirata Y. Munakata T. Saito M. Hayashi H. (2013). A serine palmitoyltransferase inhibitor blocks hepatitis C virus replication in human hepatocytes. Gastroenterology 145, 865–873. 10.1053/j.gastro.2013.06.012 23791700

[B161] Khan A. Sergi C. (2018). Sialidosis: a review of morphology and molecular biology of a rare pediatric disorder. Diagn. (Basel) 8, 29. 10.3390/diagnostics8020029 PMC6023449 29693572

[B162] Kihara A. Igarashi Y. (2004). FVT-1 is a mammalian 3-ketodihydrosphingosine reductase with an active site that faces the cytosolic side of the endoplasmic reticulum membrane. J. Biol. Chem. 279, 49243–49250. 10.1074/jbc.M405915200 15328338

[B163] Kim G. T. Devi S. Sharma A. Cho K. H. Kim S. J. Kim B. R. (2022a). Upregulation of the serine palmitoyltransferase subunit SPTLC2 by endoplasmic reticulum stress inhibits the hepatic insulin response. Exp. Mol. Med. 54, 573–584. 10.1038/s12276-022-00766-4 35513574 PMC9166747

[B164] Kim H.-L. Han M. Im D.-S. (2008b). Differential signaling of sphingosine derivatives in U937 human monocytes depends on the degree of N-methylation. Prostagl. Other Lipid Mediat. 86, 68–72. 10.1016/j.prostaglandins.2008.03.003 18467142

[B165] Kim J. L. Mestre B. Malitsky S. Itkin M. Kupervaser M. Futerman A. H. (2022b). Fatty acid transport protein 2 interacts with ceramide synthase 2 to promote ceramide synthesis. J. Biol. Chem. 298, 101735. 10.1016/j.jbc.2022.101735 35181339 PMC8931434

[B166] Kim J. L. Mestre B. Shin S. H. Futerman A. H. (2021). Ceramide synthases: reflections on the impact of dr. Lina M. Obeid. Cell Signal 82, 109958. 10.1016/j.cellsig.2021.109958 33607256

[B167] Kim W. J. Okimoto R. A. Purton L. E. Goodwin M. Haserlat S. M. Dayyani F. (2008a). Mutations in the neutral sphingomyelinase gene SMPD3 implicate the ceramide pathway in human leukemias. Blood 111, 4716–4722. 10.1182/blood-2007-10-113068 18299447 PMC2343601

[B168] Kim Y. R. Volpert G. Shin K. O. Kim S. Y. Shin S. H. Lee Y. (2017). Ablation of ceramide synthase 2 exacerbates dextran sodium sulphate-induced colitis in mice due to increased intestinal permeability. J. Cell Mol. Med. 21, 3565–3578. 10.1111/jcmm.13267 28699686 PMC5706577

[B169] Kimberlin A. N. Majumder S. Han G. Chen M. Cahoon R. E. Stone J. M. (2013). Arabidopsis 56-amino acid serine palmitoyltransferase-interacting proteins stimulate sphingolipid synthesis, are essential, and affect mycotoxin sensitivity. Plant Cell 25, 4627–4639. 10.1105/tpc.113.116145 24214397 PMC3875740

[B170] Kitatani K. Idkowiak-Baldys J. Hannun Y. A. (2008). The sphingolipid salvage pathway in ceramide metabolism and signaling. Cell Signal 20, 1010–1018. 10.1016/j.cellsig.2007.12.006 18191382 PMC2422835

[B171] Klokk T. I. Kavaliauskiene S. Sandvig K. (2016). Cross-linking of glycosphingolipids at the plasma membrane: consequences for intracellular signaling and traffic. Cell Mol. Life Sci. 73, 1301–1316. 10.1007/s00018-015-2049-1 26407609 PMC11108300

[B172] Kluepfel D. Bagli J. Baker H. Charest M. P. Kudelski A. (1972). Myriocin, a new antifungal antibiotic from Myriococcum albomyces. J. Antibiot. (Tokyo) 25, 109–115. 10.7164/antibiotics.25.109 5034807

[B173] Kolter T. Sandhoff K. (2005). Principles of lysosomal membrane digestion: stimulation of sphingolipid degradation by sphingolipid activator proteins and anionic lysosomal lipids. Annu. Rev. Cell Dev. Biol. 21, 81–103. 10.1146/annurev.cellbio.21.122303.120013 16212488

[B174] Kolter T. Sandhoff K. (2010). Lysosomal degradation of membrane lipids. FEBS Lett. 584, 1700–1712. 10.1016/j.febslet.2009.10.021 19836391

[B175] Kolzer M. Arenz C. Ferlinz K. Werth N. Schulze H. Klingenstein R. (2003). Phosphatidylinositol-3,5-Bisphosphate is a potent and selective inhibitor of acid sphingomyelinase. Biol. Chem. 384, 1293–1298. 10.1515/BC.2003.144 14515991

[B176] Komatsuya K. Kaneko K. Kasahara K. (2020). Function of platelet glycosphingolipid microdomains/lipid rafts. Int. J. Mol. Sci. 21, 5539. 10.3390/ijms21155539 32748854 PMC7432685

[B177] Korkotian E. Schwarz A. Pelled D. Schwarzmann G. Segal M. Futerman A. H. (1999). Elevation of intracellular glucosylceramide levels results in an increase in endoplasmic reticulum density and in functional calcium stores in cultured neurons. J. Biol. Chem. 274, 21673–21678. 10.1074/jbc.274.31.21673 10419477

[B178] Kornhuber J. Hoertel N. Gulbins E. (2022). The acid sphingomyelinase/ceramide system in COVID-19. Mol. Psychiatry 27, 307–314. 10.1038/s41380-021-01309-5 34608263 PMC8488928

[B179] Kraveka J. M. Li L. Szulc Z. M. Bielawski J. Ogretmen B. Hannun Y. A. (2007). Involvement of dihydroceramide desaturase in cell cycle progression in human neuroblastoma cells. J. Biol. Chem. 282, 16718–16728. 10.1074/jbc.M700647200 17283068 PMC2084375

[B180] Krebs S. Medugorac I. Rother S. Strasser K. Forster M. (2007). A missense mutation in the 3-ketodihydrosphingosine reductase FVT1 as candidate causal mutation for bovine spinal muscular atrophy. Proc. Natl. Acad. Sci. U. S. A. 104, 6746–6751. 10.1073/pnas.0607721104 17420465 PMC1868895

[B181] Kumar A. Byun H. S. Bittman R. Saba J. D. (2011). The sphingolipid degradation product trans-2-hexadecenal induces cytoskeletal reorganization and apoptosis in a JNK-dependent manner. Cell Signal 23, 1144–1152. 10.1016/j.cellsig.2011.02.009 21385609 PMC3086202

[B182] Kuo A. Checa A. Niaudet C. Jung B. Fu Z. Wheelock C. E. (2022). Murine endothelial serine palmitoyltransferase 1 (SPTLC1) is required for vascular development and systemic sphingolipid homeostasis. Elife 11, e78861. 10.7554/eLife.78861 36197001 PMC9578713

[B183] Lahiri S. Futerman A. H. (2005). LASS5 is a *bona fide* dihydroceramide synthase that selectively utilizes palmitoyl-CoA as acyl donor. J. Biol. Chem. 280, 33735–33738. 10.1074/jbc.M506485200 16100120

[B184] Lahiri S. Park H. Laviad E. L. Lu X. Bittman R. Futerman A. H. (2009). Ceramide synthesis is modulated by the sphingosine analog FTY720 via a mixture of uncompetitive and noncompetitive inhibition in an Acyl-CoA chain length-dependent manner. J. Biol. Chem. 284, 16090–16098. 10.1074/jbc.M807438200 19357080 PMC2713526

[B185] Laviad E. L. Albee L. Pankova-Kholmyansky I. Epstein S. Park H. Merrill A. H. Jr. (2008). Characterization of ceramide synthase 2: tissue distribution, substrate specificity, and inhibition by sphingosine 1-phosphate. J. Biol. Chem. 283, 5677–5684. 10.1074/jbc.M707386200 18165233

[B186] Laviad E. L. Kelly S. Merrill A. H. Jr. Futerman A. H. (2012). Modulation of ceramide synthase activity via dimerization. J. Biol. Chem. 287, 21025–21033. 10.1074/jbc.M112.363580 22539345 PMC3375526

[B187] Lee M. Lee S. Y. Bae Y. S. (2023). Functional roles of sphingolipids in immunity and their implication in disease. Exp. Mol. Med. 55, 1110–1130. 10.1038/s12276-023-01018-9 37258585 PMC10318102

[B188] Lemieux M. J. Mark B. L. Cherney M. M. Withers S. G. Mahuran D. J. James M. N. (2006). Crystallographic structure of human beta-hexosaminidase A: interpretation of Tay-Sachs mutations and loss of GM2 ganglioside hydrolysis. J. Mol. Biol. 359, 913–929. 10.1016/j.jmb.2006.04.004 16698036 PMC2910082

[B189] Lenders M. Brand E. (2021). Fabry disease: the current treatment landscape. Drugs 81, 635–645. 10.1007/s40265-021-01486-1 33721270 PMC8102455

[B190] Levy M. Futerman A. H. (2010). Mammalian ceramide synthases. IUBMB Life 62, 347–356. 10.1002/iub.319 20222015 PMC2858252

[B191] Li F. Xu R. Lin C. L. Low B. E. Cai L. Li S. (2020). Maternal and fetal alkaline ceramidase 2 is required for placental vascular integrity in mice. FASEB J. 34, 15252–15268. 10.1096/fj.202001104R 32959379 PMC8296754

[B192] Li F. Xu R. Low B. E. Lin C. L. Garcia-Barros M. Schrandt J. (2018). Alkaline ceramidase 2 is essential for the homeostasis of plasma sphingoid bases and their phosphates. FASEB J. 32, 3058–3069. 10.1096/fj.201700445RR 29401619 PMC5956249

[B193] Li Q. Yuan Q. Wang T. Zhan Y. Yang L. Fan Y. (2021b). Fumonisin B(1) inhibits cell proliferation and decreases barrier function of swine umbilical vein endothelial cells. Toxins (Basel) 13, 863. 10.3390/toxins13120863 34941701 PMC8704807

[B194] Li S. Xie T. Liu P. Wang L. Gong X. (2021a). Structural insights into the assembly and substrate selectivity of human SPT-ORMDL3 complex. Nat. Struct. Mol. Biol. 28, 249–257. 10.1038/s41594-020-00553-7 33558762

[B195] Li Y. Cao H. Dong T. Wang X. Ma L. Li K. (2023). Phosphorylation of the LCB1 subunit of Arabidopsis serine palmitoyltransferase stimulates its activity and modulates sphingolipid biosynthesis. J. Integr. Plant Biol. 65, 1585–1601. 10.1111/jipb.13461 36738228

[B196] Lin C. L. Xu R. Yi J. K. Li F. Chen J. Jones E. C. (2017). Alkaline ceramidase 1 protects mice from premature hair loss by maintaining the homeostasis of hair follicle stem cells. Stem Cell Rep. 9, 1488–1500. 10.1016/j.stemcr.2017.09.015 PMC5829345 29056331

[B197] Lingwood C. A. (2011). Glycosphingolipid functions. Cold Spring Harb. Perspect. Biol. 3, a004788. 10.1101/cshperspect.a004788 21555406 PMC3119914

[B198] Linke T. Wilkening G. Lansmann S. Moczall H. Bartelsen O. Weisgerber J. (2001). Stimulation of acid sphingomyelinase activity by lysosomal lipids and sphingolipid activator proteins. Biol. Chem. 382, 283–290. 10.1515/BC.2001.035 11308026

[B199] Liu B. Hannun Y. A. (1997). Inhibition of the neutral magnesium-dependent sphingomyelinase by glutathione. J. Biol. Chem. 272, 16281–16287. 10.1074/jbc.272.26.16281 9195931

[B200] Liu B. Obeid L. M. Hannun Y. A. (1997). Sphingomyelinases in cell regulation. Semin. Cell Dev. Biol. 8, 311–322. 10.1006/scdb.1997.0153 10024495

[B201] Liu C. Chen X. Wu W. Zhu X. (2020). A homozygotic mutation in KDSR may cause keratinization disorders and thrombocytopenia: a case report. Chin. Med. Sci. J. 35, 278–282. 10.24920/003656 32972506

[B202] Lone M. A. Aaltonen M. J. Zidell A. Pedro H. F. Morales Saute J. A. Mathew S. (2022). SPTLC1 variants associated with ALS produce distinct sphingolipid signatures through impaired interaction with ORMDL proteins. J. Clin. Invest. 132, e161908. 10.1172/JCI161908 35900868 PMC9479574

[B203] Lopez P. H. Schnaar R. L. (2009). Gangliosides in cell recognition and membrane protein regulation. Curr. Opin. Struct. Biol. 19, 549–557. 10.1016/j.sbi.2009.06.001 19608407 PMC2763983

[B204] Lowther J. Yard B. A. Johnson K. A. Carter L. G. Bhat V. T. Raman M. C. (2010). Inhibition of the PLP-dependent enzyme serine palmitoyltransferase by cycloserine: evidence for a novel decarboxylative mechanism of inactivation. Mol. Biosyst. 6, 1682–1693. 10.1039/c003743e 20445930 PMC3670083

[B205] Luberto C. Hannun Y. A. (1998). Sphingomyelin synthase, a potential regulator of intracellular levels of ceramide and diacylglycerol during SV40 transformation. Does sphingomyelin synthase account for the putative phosphatidylcholine-specific phospholipase C? J. Biol. Chem. 273, 14550–14559. 10.1074/jbc.273.23.14550 9603970

[B206] Luberto C. Hassler D. F. Signorelli P. Okamoto Y. Sawai H. Boros E. (2002). Inhibition of tumor necrosis factor-induced cell death in MCF7 by a novel inhibitor of neutral sphingomyelinase. J. Biol. Chem. 277, 41128–41139. 10.1074/jbc.M206747200 12154098

[B207] Maceyka M. Harikumar K. B. Milstien S. Spiegel S. (2012). Sphingosine-1-phosphate signaling and its role in disease. Trends Cell Biol. 22, 50–60. 10.1016/j.tcb.2011.09.003 22001186 PMC3253987

[B208] Mandala S. M. Frommer B. R. Thornton R. A. Kurtz M. B. Young N. M. Cabello M. A. (1994). Inhibition of serine palmitoyl-transferase activity by lipoxamycin. J. Antibiot. (Tokyo) 47, 376–379. 10.7164/antibiotics.47.376 8175492

[B209] Mandala S. M. Harris G. H. (2000). Isolation and characterization of novel inhibitors of sphingolipid synthesis: australifungin, viridiofungins, rustmicin, and khafrefungin. Methods Enzymol. 311, 335–348. 10.1016/s0076-6879(00)11094-8 10563338

[B210] Mandala S. M. Thornton R. A. Frommer B. R. Curotto J. E. Rozdilsky W. Kurtz M. B. (1995). The discovery of australifungin, a novel inhibitor of sphinganine N-acyltransferase from Sporormiella australis. Producing organism, fermentation, isolation, and biological activity. J. Antibiot. (Tokyo) 48, 349–356. 10.7164/antibiotics.48.349 7797434

[B211] Marchesini N. Hannun Y. A. (2004). Acid and neutral sphingomyelinases: roles and mechanisms of regulation. Biochem. Cell Biol. 82, 27–44. 10.1139/o03-091 15052326

[B212] Mashima R. Okuyama T. Ohira M. (2019). Biosynthesis of long chain base in sphingolipids in animals, plants and fungi. Future Sci. OA 6, Fso434. 10.2144/fsoa-2019-0094 31915535 PMC6920741

[B213] McGovern M. M. Dionisi-Vici C. Giugliani R. Hwu P. Lidove O. Lukacs Z. (2017). Consensus recommendation for a diagnostic guideline for acid sphingomyelinase deficiency. Genet. Med. 19, 967–974. 10.1038/gim.2017.7 28406489 PMC5589980

[B214] Mei M. Liu M. Mei Y. Zhao J. Li Y. (2023). Sphingolipid metabolism in brain insulin resistance and neurological diseases. Front. Endocrinol. (Lausanne) 14, 1243132. 10.3389/fendo.2023.1243132 37867511 PMC10587683

[B215] Mendelson K. Evans T. Hla T. (2014). Sphingosine 1-phosphate signalling. Development 141, 5–9. 10.1242/dev.094805 24346695 PMC3865745

[B216] Merrill A. H. Jr. van Echten G. Wang E. Sandhoff K. (1993). Fumonisin B1 inhibits sphingosine (sphinganine) N-acyltransferase and *de novo* sphingolipid biosynthesis in cultured neurons *in situ* . J. Biol. Chem. 268, 27299–27306. 10.1016/s0021-9258(19)74249-5 8262970

[B217] Mesika A. Ben-Dor S. Laviad E. L. Futerman A. H. (2007). A new functional motif in Hox domain-containing ceramide synthases: identification of a novel region flanking the Hox and TLC domains essential for activity. J. Biol. Chem. 282, 27366–27373. 10.1074/jbc.M703487200 17609214

[B218] Meyers-Needham M. Ponnusamy S. Gencer S. Jiang W. Thomas R. J. Senkal C. E. (2012). Concerted functions of HDAC1 and microRNA-574-5p repress alternatively spliced ceramide synthase 1 expression in human cancer cells. EMBO Mol. Med. 4, 78–92. 10.1002/emmm.201100189 22180294 PMC3376837

[B219] Michel C. van Echten-Deckert G. Rother J. Sandhoff K. Wang E. Merrill A. H. Jr. (1997). Characterization of ceramide synthesis. A dihydroceramide desaturase introduces the 4,5-trans-double bond of sphingosine at the level of dihydroceramide. J. Biol. Chem. 272, 22432–22437. 10.1074/jbc.272.36.22432 9312549

[B220] Millner A. Atilla-Gokcumen G. E. (2021). Solving the enigma: mass spectrometry and small molecule probes to study sphingolipid function. Curr. Opin. Chem. Biol. 65, 49–56. 10.1016/j.cbpa.2021.05.001 34175552

[B221] Mingione A. Pivari F. Plotegher N. Dei Cas M. Zulueta A. Bocci T. (2021). Inhibition of ceramide synthesis reduces α-synuclein proteinopathy in a cellular model of Parkinson's disease. Int. J. Mol. Sci. 22, 6469. 10.3390/ijms22126469 34208778 PMC8234676

[B222] Mistry P. K. Kishnani P. S. Balwani M. Charrow J. M. Hull J. Weinreb N. J. (2023). The Two Substrate Reduction Therapies for Type 1 Gaucher Disease Are Not Equivalent. Comment on Hughes et al. Switching between Enzyme Replacement Therapies and Substrate Reduction Therapies in Patients with Gaucher Disease: data from the Gaucher Outcome Survey (GOS). J. Clin. Med. 2022, 11, 5158. J. Clin. Med. 12, 3269. 10.3390/jcm12093269 37176709 PMC10179580

[B223] Moffatt M. F. Kabesch M. Liang L. Dixon A. L. Strachan D. Heath S. (2007). Genetic variants regulating ORMDL3 expression contribute to the risk of childhood asthma. Nature 448, 470–473. 10.1038/nature06014 17611496

[B224] Mohassel P. Donkervoort S. Lone M. A. Nalls M. Gable K. Gupta S. D. (2021). Childhood amyotrophic lateral sclerosis caused by excess sphingolipid synthesis. Nat. Med. 27, 1197–1204. 10.1038/s41591-021-01346-1 34059824 PMC9309980

[B225] Moll T. Marshall J. N. G. Soni N. Zhang S. Cooper-Knock J. Shaw P. J. (2021). Membrane lipid raft homeostasis is directly linked to neurodegeneration. Essays Biochem. 65, 999–1011. 10.1042/EBC20210026 34623437 PMC8709890

[B226] Muir A. Ramachandran S. Roelants F. M. Timmons G. Thorner J. (2014). TORC2-dependent protein kinase Ypk1 phosphorylates ceramide synthase to stimulate synthesis of complex sphingolipids. Elife 3, e03779. 10.7554/eLife.03779 25279700 PMC4217029

[B227] Mullen T. D. Hannun Y. A. Obeid L. M. (2012). Ceramide synthases at the centre of sphingolipid metabolism and biology. Biochem. J. 441, 789–802. 10.1042/BJ20111626 22248339 PMC3689921

[B228] Munakata R. Katakai H. Ueki T. Kurosaka J. Takao K. Tadano K. (2003). Total synthesis of (+)-macquarimicin A. J. Am. Chem. Soc. 125, 14722–14723. 10.1021/ja038732p 14640644

[B229] Muñoz-Guardiola P. Casas J. Megías-Roda E. Solé S. Perez-Montoyo H. Yeste-Velasco M. (2021). The anti-cancer drug ABTL0812 induces ER stress-mediated cytotoxic autophagy by increasing dihydroceramide levels in cancer cells. Autophagy 17, 1349–1366. 10.1080/15548627.2020.1761651 32397857 PMC8204958

[B230] Nădăban A. Gooris G. S. Beddoes C. M. Dalgliesh R. M. Bouwstra J. A. (2022). Phytosphingosine ceramide mainly localizes in the central layer of the unique lamellar phase of skin lipid model systems. J. Lipid Res. 63, 100258. 10.1016/j.jlr.2022.100258 35931203 PMC9421324

[B231] Nara F. Tanaka M. Hosoya T. Suzuki-Konagai K. Ogita T. (1999). Scyphostatin, a neutral sphingomyelinase inhibitor from a discomycete, Trichopeziza mollissima: taxonomy of the producing organism, fermentation, isolation, and physico-chemical properties. J. Antibiot. (Tokyo) 52, 525–530. 10.7164/antibiotics.52.525 10470675

[B232] Nishiuma T. Nishimura Y. Okada T. Kuramoto E. Kotani Y. Jahangeer S. (2008). Inhalation of sphingosine kinase inhibitor attenuates airway inflammation in asthmatic mouse model. Am. J. Physiol. Lung Cell Mol. Physiol. 294, L1085–L1093. 10.1152/ajplung.00445.2007 18359884

[B233] Ohno Y. Suto S. Yamanaka M. Mizutani Y. Mitsutake S. Igarashi Y. (2010). ELOVL1 production of C24 acyl-CoAs is linked to C24 sphingolipid synthesis. Proc. Natl. Acad. Sci. U. S. A. 107, 18439–18444. 10.1073/pnas.1005572107 20937905 PMC2973002

[B234] Ohtoyo M. Tamura M. Machinaga N. Muro F. Hashimoto R. (2015). Sphingosine 1-phosphate lyase inhibition by 2-acetyl-4-(tetrahydroxybutyl)imidazole (THI) under conditions of vitamin B6 deficiency. Mol. Cell Biochem. 400, 125–133. 10.1007/s11010-014-2268-z 25381637

[B235] Okudaira C. Ikeda Y. Kondo S. Furuya S. Hirabayashi Y. Koyano T. (2000). Inhibition of acidic sphingomyelinase by xanthone compounds isolated from Garcinia speciosa. J. Enzyme Inhib. 15, 129–138. 10.1080/14756360009030346 10938539

[B236] Olivera A. Kohama T. Tu Z. Milstien S. Spiegel S. (1998). Purification and characterization of rat kidney sphingosine kinase. J. Biol. Chem. 273, 12576–12583. 10.1074/jbc.273.20.12576 9575218

[B237] Omae F. Miyazaki M. Enomoto A. Suzuki A. (2004). Identification of an essential sequence for dihydroceramide C-4 hydroxylase activity of mouse DES2. FEBS Lett. 576, 63–67. 10.1016/j.febslet.2004.08.060 15474011

[B238] Ong W. Y. Herr D. R. Farooqui T. Ling E. A. Farooqui A. A. (2015). Role of sphingomyelinases in neurological disorders. Expert Opin. Ther. Targets 19, 1725–1742. 10.1517/14728222.2015.1071794 26243307

[B239] Ordoñez R. Fernández A. Prieto-Domínguez N. Martínez L. García-Ruiz C. Fernández-Checa J. C. (2015). Ceramide metabolism regulates autophagy and apoptotic cell death induced by melatonin in liver cancer cells. J. Pineal Res. 59, 178–189. 10.1111/jpi.12249 25975536 PMC4523438

[B240] Ordóñez-Gutiérrez L. Benito-Cuesta I. Abad J. L. Casas J. Fábrias G. Wandosell F. (2018). Dihydroceramide desaturase 1 inhibitors reduce amyloid-β levels in primary neurons from an Alzheimer's disease transgenic model. Pharm. Res. 35, 49. 10.1007/s11095-017-2312-2 29411122

[B241] Osuchowski M. F. Johnson V. J. He Q. Sharma R. P. (2004). Myriocin, a serine palmitoyltransferase inhibitor, alters regional brain neurotransmitter levels without concurrent inhibition of the brain sphingolipid biosynthesis in mice. Toxicol. Lett. 147, 87–94. 10.1016/j.toxlet.2003.10.016 14700532

[B242] Pan W. Chen K. J. Huang Y. C. (2022). Ceramide synthase 6 antisense RNA 1 contributes to the progression of breast cancer by sponging miR-16-5p to upregulate ubiquitin-conjugating enzyme E2C. Anticancer Drugs 33, 913–922. 10.1097/CAD.0000000000001381 36136991

[B243] Pan X. Dutta D. Lu S. Bellen H. J. Wei Z. Y. Jin Y. Y. (2023). Epitranscriptomic investigation of myopia-associated RNA editing in the retina. Front. Neurosci. 17, 1220114. 10.3389/fnins.2023.1220114 37449273 PMC10336353

[B244] Pani T. Rajput K. Kar A. Sharma H. Basak R. Medatwal N. (2021). Alternative splicing of ceramide synthase 2 alters levels of specific ceramides and modulates cancer cell proliferation and migration in Luminal B breast cancer subtype. Cell Death Dis. 12, 171. 10.1038/s41419-021-03436-x 33568634 PMC7876150

[B245] Panjarian S. Kozhaya L. Arayssi S. Yehia M. Bielawski J. Bielawska A. (2008). *De novo* N-palmitoylsphingosine synthesis is the major biochemical mechanism of ceramide accumulation following p53 up-regulation. Prostagl. Other Lipid Mediat 86, 41–48. 10.1016/j.prostaglandins.2008.02.004 18400537

[B246] Park J. H. Schuchman E. H. (2006). Acid ceramidase and human disease. Biochim. Biophys. Acta 1758, 2133–2138. 10.1016/j.bbamem.2006.08.019 17064658

[B247] Park K. H. Ye Z. W. Zhang J. Hammad S. M. Townsend D. M. Rockey D. C. (2019). 3-ketodihydrosphingosine reductase mutation induces steatosis and hepatic injury in zebrafish. Sci. Rep. 9, 1138. 10.1038/s41598-018-37946-0 30718751 PMC6361991

[B248] Park T. S. Rosebury W. Kindt E. K. Kowala M. C. Panek R. L. (2008). Serine palmitoyltransferase inhibitor myriocin induces the regression of atherosclerotic plaques in hyperlipidemic ApoE-deficient mice. Pharmacol. Res. 58, 45–51. 10.1016/j.phrs.2008.06.005 18611440

[B249] Pastukhov O. Schwalm S. Zangemeister-Wittke U. Fabbro D. Bornancin F. Japtok L. (2014). The ceramide kinase inhibitor NVP-231 inhibits breast and lung cancer cell proliferation by inducing M phase arrest and subsequent cell death. Br. J. Pharmacol. 171, 5829–5844. 10.1111/bph.12886 25134723 PMC4290720

[B250] Patterson M. (1993). in Niemann-pick disease type C. Editors GeneReviews ((R. Adam M. P. Mirzaa G. M. Pagon R. A. Wallace S. E. Bean L. J. H. (Seattle (WA): Treasure Island). 20301473

[B251] Paugh S. W. Paugh B. S. Rahmani M. Kapitonov D. Almenara J. A. Kordula T. (2008). A selective sphingosine kinase 1 inhibitor integrates multiple molecular therapeutic targets in human leukemia. Blood 112, 1382–1391. 10.1182/blood-2008-02-138958 18511810 PMC2515133

[B252] Pavuluri P. Vadakedath S. Gundu R. Uppulety S. Kandi V. (2017). Krabbe disease: report of a rare lipid storage and neurodegenerative disorder. Cureus 9, e949. 10.7759/cureus.949 28168127 PMC5289898

[B253] Pearson J. M. Tan S. F. Sharma A. Annageldiyev C. Fox T. E. Abad J. L. (2020). Ceramide analogue SACLAC modulates sphingolipid levels and MCL-1 splicing to induce apoptosis in acute myeloid leukemia. Mol. Cancer Res. 18, 352–363. 10.1158/1541-7786.MCR-19-0619 31744877 PMC7056541

[B254] Peters F. Tellkamp F. Brodesser S. Wachsmuth E. Tosetti B. Karow U. (2020). Murine epidermal ceramide synthase 4 is a key regulator of skin barrier homeostasis. J. Invest. Dermatol 140, 1927–1937. 10.1016/j.jid.2020.02.006 32092351

[B255] Pewzner-Jung Y. Ben-Dor S. Futerman A. H. (2006). When do Lasses (longevity assurance genes) become CerS (ceramide synthases)? insights into the regulation of ceramide synthesis. J. Biol. Chem. 281, 25001–25005. 10.1074/jbc.R600010200 16793762

[B256] Pewzner-Jung Y. Park H. Laviad E. L. Silva L. C. Lahiri S. Stiban J. (2010). A critical role for ceramide synthase 2 in liver homeostasis: I. alterations in lipid metabolic pathways. J. Biol. Chem. 285, 10902–10910. 10.1074/jbc.M109.077594 20110363 PMC2856296

[B257] Pilz R. Opálka L. Majcher A. Grimm E. Van Maldergem L. Mihalceanu S. (2022). Formation of keto-type ceramides in palmoplantar keratoderma based on biallelic KDSR mutations in patients. Hum. Mol. Genet. 31, 1105–1114. 10.1093/hmg/ddab309 34686882

[B258] Pinto C. Sousa D. Ghilas V. Dardis A. Scarpa M. Macedo M. F. (2021). Acid sphingomyelinase deficiency: a clinical and immunological perspective. Int. J. Mol. Sci. 22, 12870. 10.3390/ijms222312870 34884674 PMC8657623

[B259] Pitman M. R. Powell J. A. Coolen C. Moretti P. A. Zebol J. R. Pham D. H. (2015). A selective ATP-competitive sphingosine kinase inhibitor demonstrates anti-cancer properties. Oncotarget 6, 7065–7083. 10.18632/oncotarget.3178 25788259 PMC4466670

[B260] Platt F. M. (2014). Sphingolipid lysosomal storage disorders. Nature 510, 68–75. 10.1038/nature13476 24899306

[B261] Platt F. M. Neises G. R. Dwek R. A. Butters T. D. (1994). N-butyldeoxynojirimycin is a novel inhibitor of glycolipid biosynthesis. J. Biol. Chem. 269, 8362–8365. 10.1016/s0021-9258(17)37202-2 8132559

[B262] Pothukuchi P. Agliarulo I. Pirozzi M. Rizzo R. Russo D. Turacchio G. (2021). GRASP55 regulates intra-Golgi localization of glycosylation enzymes to control glycosphingolipid biosynthesis. EMBO J. 40, e107766. 10.15252/embj.2021107766 34516001 PMC8521277

[B263] Powell J. A. Lewis A. C. Zhu W. Toubia J. Pitman M. R. Wallington-Beddoe C. T. (2017). Targeting sphingosine kinase 1 induces MCL1-dependent cell death in acute myeloid leukemia. Blood 129, 771–782. 10.1182/blood-2016-06-720433 27956387 PMC7484978

[B264] Pralhada Rao R. Vaidyanathan N. Rengasamy M. Mammen Oommen A. Somaiya N. Jagannath M. R. (2013) Sphingolipid metabolic pathway: an overview of major roles played in human diseases. J. Lipids 2013, 178910. 10.1155/2013/178910 23984075 PMC3747619

[B265] Pruett S. T. Bushnev A. Hagedorn K. Adiga M. Haynes C. A. Sullards M. C. (2008). Biodiversity of sphingoid bases ("sphingosines") and related amino alcohols. J. Lipid Res. 49, 1621–1639. 10.1194/jlr.R800012-JLR200 18499644 PMC2444003

[B266] Qin X. Yue Z. Sun B. Yang W. Xie J. Ni E. (2013). Sphingosine and FTY720 are potent inhibitors of the transient receptor potential melastatin 7 (TRPM7) channels. Br. J. Pharmacol. 168, 1294–1312. 10.1111/bph.12012 23145923 PMC3596637

[B267] Quinville B. M. Deschenes N. M. Ryckman A. E. Walia J. S. (2021). A comprehensive review: sphingolipid metabolism and implications of disruption in sphingolipid homeostasis. Int. J. Mol. Sci. 22, 5793. 10.3390/ijms22115793 34071409 PMC8198874

[B268] Rabionet M. van der Spoel A. C. Chuang C. C. von Tumpling-Radosta B. Litjens M. Bouwmeester D. (2008). Male germ cells require polyenoic sphingolipids with complex glycosylation for completion of meiosis: a link to ceramide synthase-3. J. Biol. Chem. 283, 13357–13369. 10.1074/jbc.M800870200 18308723 PMC2442322

[B269] Rahmaniyan M. Curley R. W. Jr. Obeid L. M. Hannun Y. A. Kraveka J. M. (2011). Identification of dihydroceramide desaturase as a direct *in vitro* target for fenretinide. J. Biol. Chem. 286, 24754–24764. 10.1074/jbc.M111.250779 21543327 PMC3137051

[B270] Raichur S. Brunner B. Bielohuby M. Hansen G. Pfenninger A. Wang B. (2019). The role of C16:0 ceramide in the development of obesity and type 2 diabetes: CerS6 inhibition as a novel therapeutic approach. Mol. Metab. 21, 36–50. 10.1016/j.molmet.2018.12.008 30655217 PMC6407366

[B271] Raichur S. Wang S. T. Chan P. W. Li Y. Ching J. Chaurasia B. (2014). CerS2 haploinsufficiency inhibits beta-oxidation and confers susceptibility to diet-induced steatohepatitis and insulin resistance. Cell Metab. 20, 687–695. 10.1016/j.cmet.2014.09.015 25295789

[B272] Raisova M. Goltz G. Bektas M. Bielawska A. Riebeling C. Hossini A. M. (2002). Bcl-2 overexpression prevents apoptosis induced by ceramidase inhibitors in malignant melanoma and HaCaT keratinocytes. FEBS Lett. 516, 47–52. 10.1016/s0014-5793(02)02472-9 11959101

[B273] Rajagopalan V. Canals D. Luberto C. Snider J. Voelkel-Johnson C. Obeid L. M. (2015). Critical determinants of mitochondria-associated neutral sphingomyelinase (MA-nSMase) for mitochondrial localization. Biochim. Biophys. Acta 1850, 628–639. 10.1016/j.bbagen.2014.11.019 25484313 PMC4435939

[B274] Ramani P. K. Parayil Sankaran B. (2023). “Tay-sachs disease,” in StatPearls (Florida: Treasure Island). 33232090

[B275] Rappocciolo E. Stiban J. (2019). Prokaryotic and mitochondrial lipids: a survey of evolutionary origins. Adv. Exp. Med. Biol. 1159, 5–31. 10.1007/978-3-030-21162-2_2 31502197

[B276] Regier D. S. Proia R. L. D'Azzo A. Tifft C. J. (2016). The GM1 and GM2 gangliosidoses: natural history and progress toward therapy. Pediatr. Endocrinol. Rev. 13 (Suppl 1), 663–673. 27491214 PMC8186028

[B277] Rentz S. S. Showker J. L. Meredith F. I. Riley R. T. (2005). Inhibition of sphingolipid biosynthesis decreases phosphorylated ERK2 in LLC-PK1 cells. Food Chem. Toxicol. 43, 123–131. 10.1016/j.fct.2004.09.001 15582204

[B278] Rha A. K. Maguire A. S. Martin D. R. (2021). GM1 gangliosidosis: mechanisms and management. Appl. Clin. Genet. 14, 209–233. 10.2147/TACG.S206076 33859490 PMC8044076

[B279] Riley R. T. Merrill A. H. Jr. (2019). Ceramide synthase inhibition by fumonisins: a perfect storm of perturbed sphingolipid metabolism, signaling, and disease. J. Lipid Res. 60, 1183–1189. 10.1194/jlr.S093815 31048407 PMC6602133

[B280] Riley R. T. Voss K. A. Norred W. P. Bacon C. W. Meredith F. I. Sharma R. P. (1999). Serine palmitoyltransferase inhibition reverses anti-proliferative effects of ceramide synthase inhibition in cultured renal cells and suppresses free sphingoid base accumulation in kidney of BALBc mice. Environ. Toxicol. Pharmacol. 7, 109–118. 10.1016/s1382-6689(98)00047-7 21781915

[B281] Robert J. (2004). MS-209 schering. Curr. Opin. Investig. Drugs 5 (12), 1340–1347. 15648956

[B282] Rodriguez-Cuenca S. Barbarroja N. Vidal-Puig A. (2015). Dihydroceramide desaturase 1, the gatekeeper of ceramide induced lipotoxicity. Biochim. Biophys. Acta 1851, 40–50. 10.1016/j.bbalip.2014.09.021 25283058

[B283] Rodriguez-Duran J. Pinto-Martinez A. Castillo C. Benaim G. (2019). Identification and electrophysiological properties of a sphingosine-dependent plasma membrane Ca(2+) channel in Trypanosoma cruzi. Febs J. 286, 3909–3925. 10.1111/febs.14947 31162791

[B284] Ryckman A. E. Brockhausen I. Walia J. S. (2020). Metabolism of glycosphingolipids and their role in the pathophysiology of lysosomal storage disorders. Int. J. Mol. Sci. 21, 6881. 10.3390/ijms21186881 32961778 PMC7555265

[B285] Saba J. D. Obeid L. M. Hannun Y. A. (1996). Ceramide: an intracellular mediator of apoptosis and growth suppression. Philos. Trans. R. Soc. Lond B Biol. Sci. 351, 233–240. ; discussion 240-231. 10.1098/rstb.1996.0021 8650271

[B286] Sakakura C. Sweeney E. Shirahama T. Ruan F. Solca F. Kohno M. (1997). Inhibition of MAP kinase by sphingosine and its methylated derivative, N,N-dimethylsphingosine. Int. J. Oncol. 11, 31–39. 10.3892/ijo.11.1.31 21528177

[B287] Sakamoto W. Coant N. Canals D. Obeid L. M. Hannun Y. A. (2018). Functions of neutral ceramidase in the Golgi apparatus. J. Lipid Res. 59, 2116–2125. 10.1194/jlr.M088187 30154232 PMC6210901

[B288] Samad F. Hester K. D. Yang G. Hannun Y. A. Bielawski J. (2006). Altered adipose and plasma sphingolipid metabolism in obesity: a potential mechanism for cardiovascular and metabolic risk. Diabetes 55, 2579–2587. 10.2337/db06-0330 16936207

[B289] Santos T. C. B. Dingjan T. Futerman A. H. (2022). The sphingolipid anteome: implications for evolution of the sphingolipid metabolic pathway. FEBS Lett. 596, 2345–2363. 10.1002/1873-3468.14457 35899376

[B290] Sassa T. Hirayama T. Kihara A. (2016). Enzyme activities of the ceramide synthases CERS2-6 are regulated by phosphorylation in the C-terminal region. J. Biol. Chem. 291, 7477–7487. 10.1074/jbc.M115.695858 26887952 PMC4817178

[B291] Sasset L. Chowdhury K. H. Manzo O. L. Rubinelli L. Konrad C. Maschek J. A. (2023). Sphingosine-1-phosphate controls endothelial sphingolipid homeostasis via ORMDL. EMBO Rep. 24, e54689. 10.15252/embr.202254689 36408842 PMC9827560

[B292] Savira F. Kompa A. R. Kelly D. J. Magaye R. Xiong X. Huang L. (2021). The effect of dihydroceramide desaturase 1 inhibition on endothelial impairment induced by indoxyl sulfate. Vasc. Pharmacol. 141, 106923. 10.1016/j.vph.2021.106923 34600152

[B293] Scarlatti F. Sala G. Somenzi G. Signorelli P. Sacchi N. Ghidoni R. (2003). Resveratrol induces growth inhibition and apoptosis in metastatic breast cancer cells via *de novo* ceramide signaling. Faseb J. 17, 2339–2341. 10.1096/fj.03-0292fje 14563682

[B294] Schengrund C. L. (2015). Gangliosides: glycosphingolipids essential for normal neural development and function. Trends Biochem. Sci. 40, 397–406. 10.1016/j.tibs.2015.03.007 25941169

[B295] Schiffmann S. Sandner J. Schmidt R. Birod K. Wobst I. Schmidt H. (2009). The selective COX-2 inhibitor celecoxib modulates sphingolipid synthesis. J. Lipid Res. 50, 32–40. 10.1194/jlr.M800122-JLR200 18711209

[B296] Schneider J. S. (2018). Altered expression of genes involved in ganglioside biosynthesis in substantia nigra neurons in Parkinson's disease. PLoS One 13, e0199189. 10.1371/journal.pone.0199189 29902255 PMC6002063

[B297] Schneider N. Hauser J. Oliveira M. Cazaubon E. Mottaz S. C. O'Neill B. V. (2019). Sphingomyelin in brain and cognitive development: preliminary data. eNeuro 6, ENEURO.0421–18.2019. 10.1523/ENEURO.0421-18.2019 31324675 PMC6709232

[B298] Schnute M. E. McReynolds M. D. Kasten T. Yates M. Jerome G. Rains J. W. (2012). Modulation of cellular S1P levels with a novel, potent and specific inhibitor of sphingosine kinase-1. Biochem. J. 444, 79–88. 10.1042/BJ20111929 22397330

[B299] Schorling S. Vallée B. Barz W. P. Riezman H. Oesterhelt D. (2001). Lag1p and Lac1p are essential for the Acyl-CoA-dependent ceramide synthase reaction in Saccharomyces cerevisae. Mol. Biol. Cell 12, 3417–3427. 10.1091/mbc.12.11.3417 11694577 PMC60264

[B300] Schulze H. Sandhoff K. (2011). Lysosomal lipid storage diseases. Cold Spring Harb. Perspect. Biol. 3, a004804. 10.1101/cshperspect.a004804 21502308 PMC3098676

[B301] Schumacher F. Neuber C. Finke H. Nieschalke K. Baesler J. Gulbins E. (2017). The sphingosine 1-phosphate breakdown product, (2E)-hexadecenal, forms protein adducts and glutathione conjugates *in vitro* . J. Lipid Res. 58, 1648–1660. 10.1194/jlr.M076562 28588048 PMC5538286

[B302] Schwartz N. U. Linzer R. W. Truman J. P. Gurevich M. Hannun Y. A. Senkal C. E. (2018). Decreased ceramide underlies mitochondrial dysfunction in Charcot-Marie-Tooth 2F. FASEB J. 32, 1716–1728. 10.1096/fj.201701067R 29133339 PMC5892732

[B303] Sears S. M. Dupre T. V. Shah P. P. Davis D. L. Doll M. A. Sharp C. N. (2022). Neutral ceramidase deficiency protects against cisplatin-induced acute kidney injury. J. Lipid Res. 63, 100179. 10.1016/j.jlr.2022.100179 35151662 PMC8953688

[B304] Shamseddine A. A. Airola M. V. Hannun Y. A. (2015). Roles and regulation of neutral sphingomyelinase-2 in cellular and pathological processes. Adv. Biol. Regul. 57, 24–41. 10.1016/j.jbior.2014.10.002 25465297 PMC4684640

[B305] Shin K. O. Lim C. J. Park H. Y. Kim S. Kim B. Lee Y. (2020). Activation of SIRT1 enhances epidermal permeability barrier formation through ceramide synthase 2- and 3-dependent mechanisms. J. Invest. Dermatol 140, 1435–1438. 10.1016/j.jid.2019.12.021 31958434

[B306] Shin S. H. Cho K. A. Yoon H. S. Kim S. Y. Kim H. Y. Pewzner-Jung Y. (2021). Ceramide synthase 2 null mice are protected from ovalbumin-induced asthma with higher T cell receptor signal strength in CD4+ T cells. Int. J. Mol. Sci. 22, 2713. 10.3390/ijms22052713 33800208 PMC7962461

[B307] Siddique M. M. Bikman B. T. Wang L. Ying L. Reinhardt E. Shui G. (2012). Ablation of dihydroceramide desaturase confers resistance to etoposide-induced apoptosis *in vitro* . PLoS One 7, e44042. 10.1371/journal.pone.0044042 22984457 PMC3439484

[B308] Siddique M. M. Li Y. Chaurasia B. Kaddai V. A. Summers S. A. (2015). Dihydroceramides: from bit players to lead actors. J. Biol. Chem. 290, 15371–15379. 10.1074/jbc.R115.653204 25947377 PMC4505450

[B309] Signorelli P. Conte C. Albi E. (2021). The multiple roles of sphingomyelin in Parkinson's disease. Biomolecules 11, 1311. 10.3390/biom11091311 34572524 PMC8469734

[B310] Signorelli P. Munoz-Olaya J. M. Gagliostro V. Casas J. Ghidoni R. Fabrias G. (2009). Dihydroceramide intracellular increase in response to resveratrol treatment mediates autophagy in gastric cancer cells. Cancer Lett. 282, 238–243. 10.1016/j.canlet.2009.03.020 19394759

[B311] Silva L. C. Ben David O. Pewzner-Jung Y. Laviad E. L. Stiban J. Bandyopadhyay S. (2012). Ablation of ceramide synthase 2 strongly affects biophysical properties of membranes. J. Lipid Res. 53, 430–436. 10.1194/jlr.M022715 22231783 PMC3276466

[B312] Simons M. Nave K. A. (2015). Oligodendrocytes: myelination and axonal support. Cold Spring Harb. Perspect. Biol. 8, a020479. 10.1101/cshperspect.a020479 26101081 PMC4691794

[B313] Sindhu S. Leung Y. H. Arefanian H. Madiraju S. R. M. Al-Mulla F. Ahmad R. (2021). Neutral sphingomyelinase-2 and cardiometabolic diseases. Obes. Rev. 22, e13248. 10.1111/obr.13248 33738905 PMC8365731

[B314] Siskind L. J. (2005). Mitochondrial ceramide and the induction of apoptosis. J. Bioenerg. Biomembr. 37, 143–153. 10.1007/s10863-005-6567-7 16167171 PMC2246044

[B315] Siskind L. J. Davoody A. Lewin N. Marshall S. Colombini M. (2003). Enlargement and contracture of C2-ceramide channels. Biophys. J. 85, 1560–1575. 10.1016/S0006-3495(03)74588-3 12944273 PMC1303332

[B316] Siskind L. J. Kolesnick R. N. Colombini M. (2006). Ceramide forms channels in mitochondrial outer membranes at physiologically relevant concentrations. Mitochondrion 6, 118–125. 10.1016/j.mito.2006.03.002 16713754 PMC2246045

[B317] Skácel J. Slusher B. S. Tsukamoto T. (2021). Small molecule inhibitors targeting biosynthesis of ceramide, the central hub of the sphingolipid network. J. Med. Chem. 64, 279–297. 10.1021/acs.jmedchem.0c01664 33395289 PMC8023021

[B318] Smith D. C. Lord J. M. Roberts L. M. Johannes L. (2004). Glycosphingolipids as toxin receptors. Semin. Cell Dev. Biol. 15, 397–408. 10.1016/j.semcdb.2004.03.005 15207830

[B319] Snider A. J. Ruiz P. Obeid L. M. Oates J. C. (2013). Inhibition of sphingosine kinase-2 in a murine model of lupus nephritis. PLoS One 8, e53521. 10.1371/journal.pone.0053521 23301082 PMC3536755

[B320] Sofi M. H. Tian L. Schutt S. Khan I. Choi H. J. Wu Y. (2022). Ceramide synthase 6 impacts T-cell allogeneic response and graft-versus-host disease through regulating N-RAS/ERK pathway. Leukemia 36, 1907–1915. 10.1038/s41375-022-01581-6 35513703 PMC9256768

[B321] Spiegel S. Milstien S. (2003). Sphingosine-1-phosphate: an enigmatic signalling lipid. Nat. Rev. Mol. Cell Biol. 4, 397–407. 10.1038/nrm1103 12728273

[B322] Sridevi P. Alexander H. Laviad E. L. Min J. Mesika A. Hannink M. (2010). Stress-induced ER to Golgi translocation of ceramide synthase 1 is dependent on proteasomal processing. Exp. Cell Res. 316, 78–91. 10.1016/j.yexcr.2009.09.027 19800881 PMC2791511

[B323] Sridevi P. Alexander H. Laviad E. L. Pewzner-Jung Y. Hannink M. Futerman A. H. (2009). Ceramide synthase 1 is regulated by proteasomal mediated turnover. Biochim. Biophys. Acta 1793, 1218–1227. 10.1016/j.bbamcr.2009.04.006 19393694 PMC2724657

[B324] Srivastava S. Shaked H. M. Gable K. Gupta S. D. Pan X. Somashekarappa N. (2023). SPTSSA variants alter sphingolipid synthesis and cause a complex hereditary spastic paraplegia. Brain 146, 1420–1435. 10.1093/brain/awac460 36718090 PMC10319774

[B325] Stankeviciute G. Tang P. Ashley B. Chamberlain J. D. Hansen M. E. B. Coleman A. (2022). Convergent evolution of bacterial ceramide synthesis. Nat. Chem. Biol. 18, 305–312. 10.1038/s41589-021-00948-7 34969973 PMC8891067

[B326] Stefanić S. Spycher C. Morf L. Fabriàs G. Casas J. Schraner E. (2010). Glucosylceramide synthesis inhibition affects cell cycle progression, membrane trafficking, and stage differentiation in Giardia lamblia. J. Lipid Res. 51, 2527–2545. 10.1194/jlr.M003392 20335568 PMC2918437

[B327] Stiban J. (2019). Introduction: enigmas of sphingolipids. Adv. Exp. Med. Biol. 1159, 1–3. 10.1007/978-3-030-21162-2_1 31502196

[B328] Stiban J. Caputo L. Colombini M. (2008). Ceramide synthesis in the endoplasmic reticulum can permeabilize mitochondria to proapoptotic proteins. J. Lipid Res. 49, 625–634. 10.1194/jlr.M700480-JLR200 18073406

[B329] Stiban J. Fistere D. Colombini M. (2006). Dihydroceramide hinders ceramide channel formation: implications on apoptosis. Apoptosis 11, 773–780. 10.1007/s10495-006-5882-8 16532372

[B330] Stiban J. Perera M. (2015). Very long chain ceramides interfere with C16-ceramide-induced channel formation: a plausible mechanism for regulating the initiation of intrinsic apoptosis. Biochim. Biophys. Acta 1848, 561–567. 10.1016/j.bbamem.2014.11.018 25462172

[B331] Stiban J. Tidhar R. Futerman A. H. (2010). Ceramide synthases: roles in cell physiology and signaling. Adv. Exp. Med. Biol. 688, 60–71. 10.1007/978-1-4419-6741-1_4 20919646

[B332] Stockmann-Juvala H. Savolainen K. (2008). A review of the toxic effects and mechanisms of action of fumonisin B1. Hum. Exp. Toxicol. 27, 799–809. 10.1177/0960327108099525 19244287

[B333] Tabas I. (1999). Secretory sphingomyelinase. Chem. Phys. Lipids 102, 123–130. 10.1016/s0009-3084(99)00080-8 11001566

[B334] Takabe K. Paugh S. W. Milstien S. Spiegel S. (2008). Inside-out" signaling of sphingosine-1-phosphate: therapeutic targets. Pharmacol. Rev. 60, 181–195. 10.1124/pr.107.07113 18552276 PMC2695666

[B335] Tan-Chen S. Guitton J. Bourron O. Le Stunff H. Hajduch E. (2020). Sphingolipid metabolism and signaling in skeletal muscle: from physiology to physiopathology. Front. Endocrinol. (Lausanne) 11, 491. 10.3389/fendo.2020.00491 32849282 PMC7426366

[B336] Tancini B. Magini A. Bortot B. Polchi A. Urbanelli L. Sonnino S. (2012). Β-hexosaminidase over-expression affects lysosomal glycohydrolases expression and glycosphingolipid metabolism in mammalian cells. Mol. Cell Biochem. 363, 109–118. 10.1007/s11010-011-1163-0 22147196

[B337] Tani M. Hannun Y. A. (2007). Neutral sphingomyelinase 2 is palmitoylated on multiple cysteine residues. Role of palmitoylation in subcellular localization. J. Biol. Chem. 282, 10047–10056. 10.1074/jbc.M611249200 17272284

[B338] Taniguchi M. Okazaki T. (2021). Role of ceramide/sphingomyelin (SM) balance regulated through "SM cycle" in cancer. Cell Signal 87, 110119. 10.1016/j.cellsig.2021.110119 34418535

[B339] Taouji S. Higa A. Delom F. Palcy S. Mahon F. X. Pasquet J. M. (2013). Phosphorylation of serine palmitoyltransferase long chain-1 (SPTLC1) on tyrosine 164 inhibits its activity and promotes cell survival. J. Biol. Chem. 288, 17190–17201. 10.1074/jbc.M112.409185 23629659 PMC3682524

[B340] Ternes P. Franke S. Zahringer U. Sperling P. Heinz E. (2002). Identification and characterization of a sphingolipid delta 4-desaturase family. J. Biol. Chem. 277, 25512–25518. 10.1074/jbc.M202947200 11937514

[B341] Testai F. D. Landek M. A. Goswami R. Ahmed M. Dawson G. (2004). Acid sphingomyelinase and inhibition by phosphate ion: role of inhibition by phosphatidyl-myo-inositol 3,4,5-triphosphate in oligodendrocyte cell signaling. J. Neurochem. 89, 636–644. 10.1046/j.1471-4159.2004.02374.x 15086520

[B342] Tolan D. Conway A. M. Pyne N. J. Pyne S. (1997). Sphingosine prevents diacylglycerol signaling to mitogen-activated protein kinase in airway smooth muscle. Am. J. Physiol. 273, C928–C936. 10.1152/ajpcell.1997.273.3.C928 9316414

[B343] Tomasello D. L. Kim J. L. Khodour Y. McCammon J. M. Mitalipova M. Jaenisch R. (2022). 16pdel lipid changes in iPSC-derived neurons and function of FAM57B in lipid metabolism and synaptogenesis. iScience 25, 103551. 10.1016/j.isci.2021.103551 34984324 PMC8693007

[B344] Tonnetti L. Veri M. C. Bonvini E. D'Adamio L. (1999). A role for neutral sphingomyelinase-mediated ceramide production in T cell receptor-induced apoptosis and mitogen-activated protein kinase-mediated signal transduction. J. Exp. Med. 189, 1581–1589. 10.1084/jem.189.10.1581 10330437 PMC2193632

[B345] Tosetti B. Brodesser S. Brunn A. Deckert M. Bluher M. Doehner W. (2020). A tissue-specific screen of ceramide expression in aged mice identifies ceramide synthase-1 and ceramide synthase-5 as potential regulators of fiber size and strength in skeletal muscle. Aging Cell 19, e13049. 10.1111/acel.13049 31692231 PMC6974707

[B346] Trayssac M. Hannun Y. A. Obeid L. M. (2018). Role of sphingolipids in senescence: implication in aging and age-related diseases. J. Clin. Invest. 128, 2702–2712. 10.1172/JCI97949 30108193 PMC6025964

[B347] Triola G. Fabrias G. Dragusin M. Niederhausen L. Broere R. Llebaria A. (2004). Specificity of the dihydroceramide desaturase inhibitor N-[(1R,2S)-2-hydroxy-1-hydroxymethyl-2-(2-tridecyl-1-cyclopropenyl)ethyl]octanamide (GT11) in primary cultured cerebellar neurons. Mol. Pharmacol. 66, 1671–1678. 10.1124/mol.104.003681 15371559

[B348] Tzou F. Y. Su T. Y. Lin W. S. Kuo H. C. Yu Y. L. Yeh Y. H. (2021). Dihydroceramide desaturase regulates the compartmentalization of Rac1 for neuronal oxidative stress. Cell Rep. 35, 108972. 10.1016/j.celrep.2021.108972 33852856

[B349] Uchida R. Tomoda H. Arai M. Omura S. (2001). Chlorogentisylquinone, a new neutral sphingomyelinase inhibitor, produced by a marine fungus. J. Antibiot. (Tokyo) 54, 882–889. 10.7164/antibiotics.54.882 11827029

[B350] Uchida R. Tomoda H. Dong Y. Omura S. (1999). Alutenusin, a specific neutral sphingomyelinase inhibitor, produced by Penicillium sp. FO-7436. J. Antibiot. (Tokyo) 52, 572–574. 10.7164/antibiotics.52.572 10470682

[B351] Umehara T. Sudoh M. Yasui F. Matsuda C. Hayashi Y. Chayama K. (2006). Serine palmitoyltransferase inhibitor suppresses HCV replication in a mouse model. Biochem. Biophys. Res. Commun. 346, 67–73. 10.1016/j.bbrc.2006.05.085 16750511

[B352] Upadhyaya P. Kumar A. Byun H. S. Bittman R. Saba J. D. Hecht S. S. (2012). The sphingolipid degradation product trans-2-hexadecenal forms adducts with DNA. Biochem. Biophys. Res. Commun. 424, 18–21. 10.1016/j.bbrc.2012.06.012 22727907 PMC3402648

[B353] Vallee B. Riezman H. (2005). Lip1p: a novel subunit of acyl-CoA ceramide synthase. EMBO J. 24, 730–741. 10.1038/sj.emboj.7600562 15692566 PMC549621

[B354] Venable M. E. Blobe G. C. Obeid L. M. (1994). Identification of a defect in the phospholipase D/diacylglycerol pathway in cellular senescence. J. Biol. Chem. 269, 26040–26044. 10.1016/s0021-9258(18)47156-6 7929315

[B355] Vitner E. B. Futerman A. H. (2013). Neuronal forms of Gaucher disease. Handb. Exp. Pharmacol., 405–419. 10.1007/978-3-7091-1511-4_20 23563668

[B356] Vitner E. B. Vardi A. Cox T. M. Futerman A. H. (2015). Emerging therapeutic targets for Gaucher disease. Expert Opin. Ther. Targets 19, 321–334. 10.1517/14728222.2014.981530 25416676

[B357] Wang J. Chen Y. L. Li Y. K. Chen D. K. He J. F. Yao N. (2021b). Functions of sphingolipids in pathogenesis during host-pathogen interactions. Front. Microbiol. 12, 701041. 10.3389/fmicb.2021.701041 34408731 PMC8366399

[B358] Wang K. Xu R. Schrandt J. Shah P. Gong Y. Z. Preston C. (2015). Alkaline ceramidase 3 deficiency results in Purkinje cell degeneration and cerebellar ataxia due to dyshomeostasis of sphingolipids in the brain. PLoS Genet. 11, e1005591. 10.1371/journal.pgen.1005591 26474409 PMC4608763

[B359] Wang Y. Niu Y. Zhang Z. Gable K. Gupta S. D. Somashekarappa N. (2021a). Structural insights into the regulation of human serine palmitoyltransferase complexes. Nat. Struct. Mol. Biol. 28, 240–248. 10.1038/s41594-020-00551-9 33558761 PMC9812531

[B360] Wascholowski V. Giannis A. (2006). Sphingolactones: selective and irreversible inhibitors of neutral sphingomyelinase. Angew. Chem. Int. Ed. Engl. 45, 827–830. 10.1002/anie.200501983 16365835

[B361] Wattenberg B. W. (2021). Kicking off sphingolipid biosynthesis: structures of the serine palmitoyltransferase complex. Nat. Struct. Mol. Biol. 28, 229–231. 10.1038/s41594-021-00562-0 33558763

[B362] Weiss L. Jung K. M. Nalbandian A. Llewellyn K. Yu H. Ta L. (2021). Ceramide contributes to pathogenesis and may be targeted for therapy in VCP inclusion body myopathy. Hum. Mol. Genet. 29, 3945–3953. 10.1093/hmg/ddaa248 33410456 PMC8485215

[B363] Wells G. B. Dickson R. C. Lester R. L. (1998). Heat-induced elevation of ceramide in *Saccharomyces cerevisiae* via *de novo* synthesis. J. Biol. Chem. 273, 7235–7243. 10.1074/jbc.273.13.7235 9516416

[B364] Wells G. B. Lester R. L. (1983). The isolation and characterization of a mutant strain of *Saccharomyces cerevisiae* that requires a long chain base for growth and for synthesis of phosphosphingolipids. J. Biol. Chem. 258, 10200–10203. 10.1016/s0021-9258(17)44439-5 6350287

[B365] White C. Alshaker H. Cooper C. Winkler M. Pchejetski D. (2016). The emerging role of FTY720 (Fingolimod) in cancer treatment. Oncotarget 7, 23106–23127. 10.18632/oncotarget.7145 27036015 PMC5029614

[B366] Widder K. Harauz G. Hinderberger D. (2020). Myelin basic protein (MBP) charge variants show different sphingomyelin-mediated interactions with myelin-like lipid monolayers. Biochim. Biophys. Acta Biomembr. 1862, 183077. 10.1016/j.bbamem.2019.183077 31805269

[B367] Wijesinghe D. S. Brentnall M. Mietla J. A. Hoeferlin L. A. Diegelmann R. F. Boise L. H. (2014). Ceramide kinase is required for a normal eicosanoid response and the subsequent orderly migration of fibroblasts. J. Lipid Res. 55, 1298–1309. 10.1194/jlr.M048207 24823941 PMC4076082

[B368] Wu L. Zhang Y. Zi J. Yan Y. Yu L. Lin D. (2022). Case report: compound heterozygous mutations in the KDSR gene cause progressive keratodermia and thrombocytopenia. Front. Pediatr. 10, 940618. 10.3389/fped.2022.940618 35958175 PMC9360485

[B369] Xu R. Garcia-Barros M. Wen S. Li F. Lin C. L. Hannun Y. A. (2018). Tumor suppressor p53 links ceramide metabolism to DNA damage response through alkaline ceramidase 2. Cell Death Differ. 25, 841–856. 10.1038/s41418-017-0018-y 29229990 PMC5943524

[B370] Xu R. Sun W. Jin J. Obeid L. M. Mao C. (2010). Role of alkaline ceramidases in the generation of sphingosine and its phosphate in erythrocytes. FASEB J. 24, 2507–2515. 10.1096/fj.09-153635 20207939 PMC2887272

[B371] Xu R. Wang K. Mileva I. Hannun Y. A. Obeid L. M. Mao C. (2016). Alkaline ceramidase 2 and its bioactive product sphingosine are novel regulators of the DNA damage response. Oncotarget 7, 18440–18457. 10.18632/oncotarget.7825 26943039 PMC4951300

[B372] Yang R.-X. Pan Q. Liu X.-L. Zhou D. Xin F.-Z. Zhao Z.-H. (2019). Therapeutic effect and autophagy regulation of myriocin in nonalcoholic steatohepatitis. Lipids Health Dis. 18, 179. 10.1186/s12944-019-1118-0 31639005 PMC6805575

[B373] Yard B. A. Carter L. G. Johnson K. A. Overton I. M. Dorward M. Liu H. (2007). The structure of serine palmitoyltransferase; gateway to sphingolipid biosynthesis. J. Mol. Biol. 370, 870–886. 10.1016/j.jmb.2007.04.086 17559874

[B374] Yatsu F. M. (1971). Sphingolipidoses. Calif. Med. 114, 1–6. PMC1501855 5551302

[B375] Yi J. K. Xu R. Obeid L. M. Hannun Y. A. Airola M. V. Mao C. (2022). Alkaline ceramidase catalyzes the hydrolysis of ceramides via a catalytic mechanism shared by Zn2+-dependent amidases. PLoS One 17, e0271540. 10.1371/journal.pone.0271540 36048828 PMC9436119

[B376] Yi X. Tang X. Li T. Chen L. He H. Wu X. (2023). Therapeutic potential of the sphingosine kinase 1 inhibitor, PF-543. Biomed. Pharmacother. 163, 114401. 10.1016/j.biopha.2023.114401 37167721

[B377] Yin Y. Xu M. Gao J. Li M. (2018). Alkaline ceramidase 3 promotes growth of hepatocellular carcinoma cells via regulating S1P/S1PR2/PI3K/AKT signaling. Pathol. Res. Pract. 214, 1381–1387. 10.1016/j.prp.2018.07.029 30097213

[B378] Yu L. Jiang Y. Xu L. Jin J. Pei Z. Zhu J. (2022). Theoretical study of myriocin-binding mechanism targeting serine palmitoyltransferase. Chem. Biol. Drug Des. 99, 373–381. 10.1111/cbdd.13991 34862732

[B379] Yu R. K. Tsai Y. T. Ariga T. (2012). Functional roles of gangliosides in neurodevelopment: an overview of recent advances. Neurochem. Res. 37, 1230–1244. 10.1007/s11064-012-0744-y 22410735 PMC3895947

[B380] Yura Y. Masui A. Hamada M. (2020). Inhibitors of ceramide- and sphingosine-metabolizing enzymes as sensitizers in radiotherapy and chemotherapy for head and neck squamous cell carcinoma. Cancers (Basel) 12, 2062. 10.3390/cancers12082062 32722626 PMC7463798

[B381] Zarghooni M. Bukovac S. Tropak M. Callahan J. Mahuran D. (2004). An alpha-subunit loop structure is required for GM2 activator protein binding by beta-hexosaminidase A. Biochem. Biophys. Res. Commun. 324, 1048–1052. 10.1016/j.bbrc.2004.09.159 15485660 PMC2918538

[B382] Zelnik I. D. Rozman B. Rosenfeld-Gur E. Ben-Dor S. Futerman A. H. (2019). A stroll down the CerS lane. Adv. Exp. Med. Biol. 1159, 49–63. 10.1007/978-3-030-21162-2_4 31502199

[B383] Zeng H. Y. Li C. Y. Yao N. (2020). Fumonisin B1: a tool for exploring the multiple functions of sphingolipids in plants. Front. Plant Sci. 11, 600458. 10.3389/fpls.2020.600458 33193556 PMC7652989

[B384] Zhang K. Wu R. Mei F. Zhou Y. He L. Liu Y. (2021a). Phosphorylated LASS2 inhibits prostate carcinogenesis via negative regulation of Wnt/β-catenin signaling. J. Cell Biochem. 122, 1048–1061. 10.1002/jcb.29926 33852174

[B385] Zhang T. de Waard A. A. Wuhrer M. Spaapen R. M. (2019). The role of glycosphingolipids in immune cell functions. Front. Immunol. 10, 90. 10.3389/fimmu.2019.00090 30761148 PMC6361815

[B386] Zhang X. Sakamoto W. Canals D. Ishibashi M. Matsuda M. Nishida K. (2021b). Ceramide synthase 2-C(24:1) -ceramide axis limits the metastatic potential of ovarian cancer cells. Faseb J. 35, e21287. 10.1096/fj.202001504RR 33423335 PMC8237407

[B387] Zhao L. Spassieva S. Gable K. Gupta S. D. Shi L. Y. Wang J. (2015). Elevation of 20-carbon long chain bases due to a mutation in serine palmitoyltransferase small subunit b results in neurodegeneration. Proc. Natl. Acad. Sci. U. S. A. 112, 12962–12967. 10.1073/pnas.1516733112 26438849 PMC4620873

[B388] Zhao Y. Liu Z. Wang L. Liu H. (2022). Fumonisin B1 as a tool to explore sphingolipid roles in arabidopsis primary root development. Int. J. Mol. Sci. 23, 12925. 10.3390/ijms232112925 36361715 PMC9654530

[B389] Zheng C. Terreni M. Sollogoub M. Zhang Y. (2021). Functional role of glycosphingolipids in cancer. Curr. Med. Chem. 28, 3913–3924. 10.2174/0929867327666200831132200 32867632

[B390] Zhu W. K. Xu W. H. Wang J. Huang Y. Q. Abudurexiti M. Qu Y. Y. (2020). Decreased SPTLC1 expression predicts worse outcomes in ccRCC patients. J. Cell Biochem. 121, 1552–1562. 10.1002/jcb.29390 31512789

